# BioMaster: Multi-agent system for automated bioinformatics analysis workflow

**DOI:** 10.1016/j.patter.2026.101611

**Published:** 2026-07-07

**Authors:** Houcheng Su, Junning Feng, Yawen Lu, Yucheng Xu, Jinming Yang, Haojie Lu, Jixin Yang, Xu Yang, Sirui Xie, Weicai Long, Chengrui Wang, Yusen Hou, Tingyu Zhu, Yanlin Zhang

**Affiliations:** 1 Data Science and Analytics Thrust, Hong Kong University of Science and Technology (Guangzhou), Guangzhou, Guangdong, China; 2 Guangdong Hospital of Traditional Chinese Medicine, Guangzhou, Guangdong, China

**Keywords:** bioinformatics agent, multi-agent system, retrieval-augmented generation, omics data analysis, pipeline, large language model

## Abstract

The growing volume and complexity of biological data have made bioinformatics workflows increasingly labor-intensive, error-prone, and difficult to scale. Large language model-based agents offer potential for automation but often fail in complex, multi-step analyses because of limited robustness. We present BioMaster, a multi-agent framework that integrates workflow planning, execution, error recovery, and output validation. BioMaster incorporates a dual retrieval-augmented design to leverage domain knowledge for tool selection, parameterization, and adaptation across tasks. A dedicated debug agent supports real-time error detection and correction, while memory optimization enables long, multi-stage workflows. In benchmarking across 49 bioinformatics tasks spanning 102 tools, BioMaster completed substantially more workflows than did existing automated systems, particularly in complex, interdependent pipelines. BioMaster supports both proprietary and open-source language models, enabling flexible deployment across different computational settings.

## Highlights

• BioMaster is a multi-agent system for automated bioinformatics workflows
• BioMaster integrates a dual RAG design for planning and execution knowledge
• BioMaster is evaluated on a wide range of bioinformatics tasks spanning 102 tools

## The bigger picture

Bioinformatics has become central to modern life sciences, yet many analyses still depend on complex, multi-step workflows that integrate diverse software tools and heterogeneous data formats. Despite advances in workflow managers, researchers are often required to manually select methods, configure parameters, and resolve software dependencies. This process is time-consuming, error-prone, and difficult to reproduce, creating a persistent barrier for researchers without strong computational backgrounds and limiting the scalability of biological data analysis. Recent advances in large language models (LLMs) have created new opportunities for automating scientific workflows through natural language interaction. This work introduces a knowledge-guided, multi-agent framework that leverages LLMs to automate the construction and execution of bioinformatics workflows from high-level task descriptions. By combining structured workflow planning with retrieval of tool-specific knowledge, the system reduces the need for manual pipeline engineering while maintaining flexibility across diverse analysis tasks.

## Teaser

BioMaster is a knowledge-guided multi-agent framework that automates bioinformatics workflow planning, execution, debugging, and validation. Across 49 tasks and 102 tools, it improves workflow completion over existing automated systems and supports both proprietary and open-source language models.

## Introduction

Advances in high-throughput technologies such as whole-genome sequencing (WGS),^1^ single-cell transcriptomics,^2^ and 3D genome mapping^3^ have dramatically expanded the volume and diversity of biological data.^4^^,^^5^^,^^6^ Extracting biological insight from these data requires multi-step computational workflows that integrate numerous software tools, from standard preprocessing—such as read alignment and quality control—to specialized downstream analyses including chromatin loop detection, topologically associating domain annotation,^7^^,^^8^^,^^9^ spatial domain segmentation, and microbial community profiling. While standardized solutions exist for certain early-stage tasks, downstream analyses remain fragmented, often lacking consensus “gold standard” tools. Researchers must therefore manually identify and evaluate alternative methods, resolve software dependencies, and interpret heterogeneous outputs. These processes are labor-intensive, error prone, and sensitive to computational environment inconsistencies,^10^^,^^11^ posing significant barriers for researchers without advanced computational expertise and limiting scalability and reproducibility.

Workflow management systems such as Snakemake^12^ and Nextflow^10^^,^^11^ provide robust frameworks for executing bioinformatics pipelines but require substantial scripting and configuration skills. Web-based platforms including Galaxy,^13^ iDEP,^14^ and ICARUS^15^ offer more accessible interfaces but often at the expense of flexibility, limiting their applicability to highly customized analyses and raising potential data privacy concerns. Crucially, both categories still depend on user expertise for tool selection, parameter tuning, and troubleshooting, leaving a significant automation gap.

Recent advances in AI, particularly large language models (LLMs),^16^^,^^17^ have created opportunities to automate scientific workflows via intelligent agent-based systems. Domain-specific agents have demonstrated promising results in fields such as chemistry and molecular design, exemplified by ChemCrow^18^ and MultiMol,^19^ which autonomously propose synthetic routes, conduct virtual experiments, and optimize molecular structures. Knowledge-graph-driven systems such as SciToolAgent show that graph-based retrieval can intelligently orchestrate hundreds of tools with built-in safety checks across biology, chemistry, and materials science.^20^ In bioinformatics, AutoBA^21^ showcased LLM-driven automation for multi-omics workflow planning. However, as a single-agent system, AutoBA focuses on pipeline design and struggles with executing complex, interdependent tasks, robust error detection, and recovery. Multi-agent architectures offer a pathway to overcome these limitations by delegating specialized responsibilities—such as planning, execution, and validation—to dedicated agents. Tools such as CellAgent^22^ and other single-cell-focused systems^23^ illustrate the potential of role-specific agents, but most existing frameworks target narrow application domains and lack the versatility to manage diverse modalities such as epigenomics, proteomics, and population genetics. CRISPR-GPT^24^ demonstrates fully AI-guided CRISPR design and data analysis with experimental validation across knockout and epigenetic activation assays. Technical barriers also persist: long and complex workflows challenge LLMs’ memory management^16^; reliance on static, manually curated knowledge limits adaptability in rapidly evolving fields, and the absence of robust, automated error recovery mechanisms risks error propagation and undermines analytical integrity. In addition, many existing systems rely heavily on proprietary models such as GPT-4, raising concerns regarding transparency, reproducibility, accessibility, and long-term sustainability.

Here, we present BioMaster, a knowledge-enabled multi-agent framework for fully automated biological data analysis. BioMaster integrates four specialized agents within a decision-making system: a plan agent for workflow design, a task agent for command generation and execution, a debug agent for real-time error detection and correction, and a check agent for rigorous output validation. Central to BioMaster’s adaptability is a dual retrieval-augmented generation (RAG) system that dynamically queries continuously updated, domain-specific knowledge repositories containing curated workflows, detailed tool documentation, and methodological best practices. We evaluated BioMaster across 49 tasks spanning multiple omics modalities—including transcriptomics, epigenomics, metagenomics, and 3D genome architecture—covering 102 bioinformatics tools. BioMaster autonomously executed multi-step workflows, effectively recovered from execution errors, and outperformed existing automated systems in versatility, robustness, and reproducibility. By unifying planning, execution, error correction, and validation, BioMaster reduces manual workload, promotes methodological consistency, and enables scalable, accessible bioinformatics analyses across a broad spectrum of research applications.

## Results

### BioMaster is a crew of agents for biological data analysis

**Figure 1:**
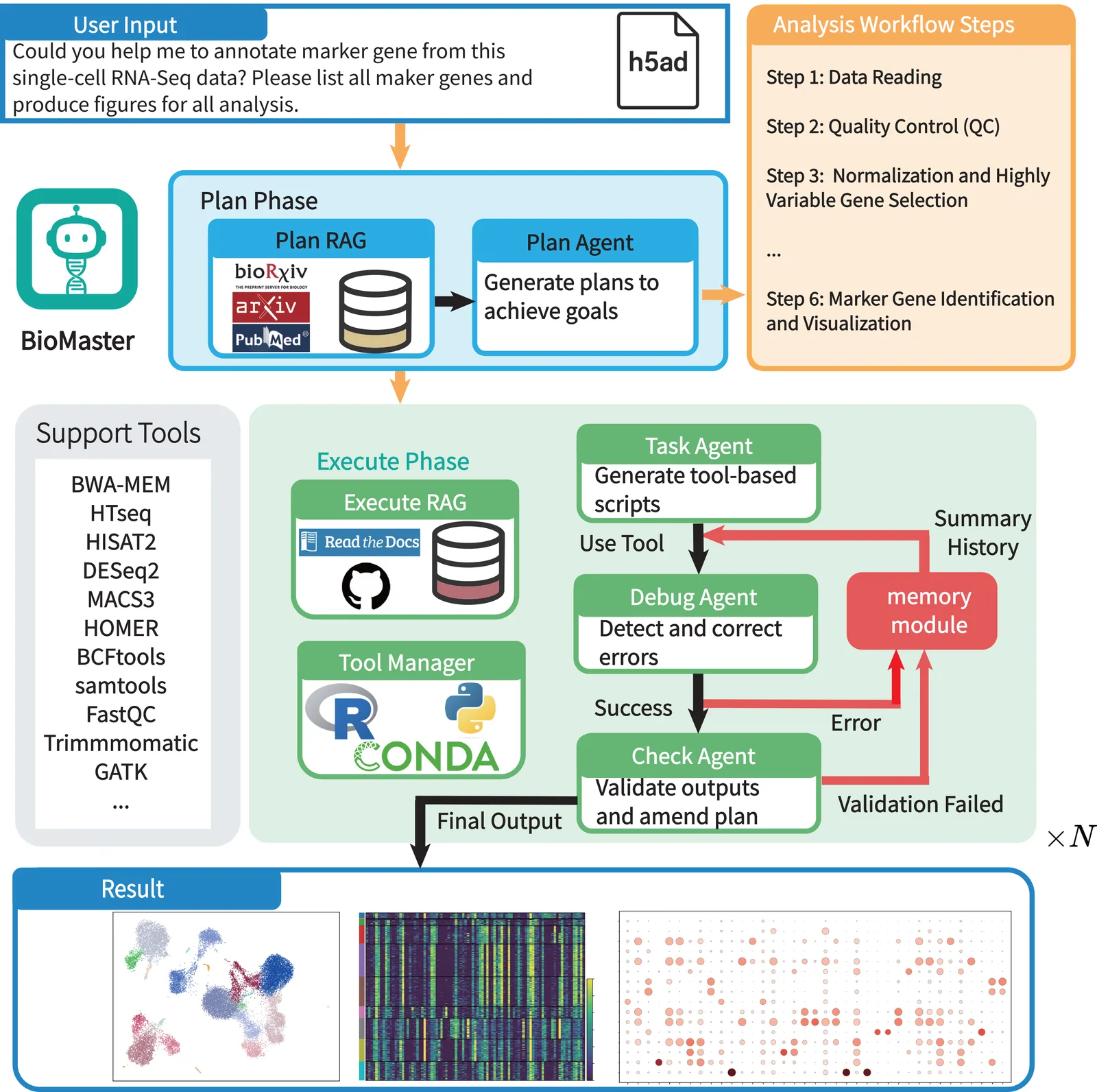
Overview of BioMaster The system receives a user-defined analysis goal (example shown for single-cell marker gene identification and visualization) and generates an executable workflow. In the planning phase, the plan agent queries the plan RAG knowledge base to retrieve relevant methodological workflows and outputs a structured, stepwise plan. In the execution phase, the task agent uses execute RAG to generate and run tool-specific scripts, while the debug agent monitors execution, resolves errors using retrieved troubleshooting information, and maintains a condensed summary of diagnostic history. The check agent validates outputs at each step, amends the plan if discrepancies are detected, and triggers re-execution when necessary. This cycle repeats until all steps are successfully completed.

We developed BioMaster, a multi-agent framework that autonomously translates natural language task descriptions into executable bioinformatics workflows (Figure 1). It comprises four specialized agents—plan, task, debug, and check—that operate in an iterative loop to plan, execute, monitor, and validate each step of the analysis. Each agent is role based, with its functionality guided by a dedicated prompt. This process is supported by a dual RAG architecture that dynamically injects expert knowledge at key decision points. The plan RAG module provides high-level methodological knowledge, such as common analysis strategies and workflow templates, which the plan agent uses to generate a step-by-step analysis plan. Each step specifies the required inputs and outputs and the appropriate software tools. The execute RAG, in contrast, supplies low-level technical details—including installation commands, configuration parameters, and command-line examples—that inform the task and debug agents during execution and error handling. While the dual RAG design improves performance on complex bioinformatics workflows, it requires an upfront knowledge curation step. This process is knowledge based rather than code based, involving the provision of documentation, example commands, and expected input-output specifications. In our study, adding a new tool typically requires less than 10 min when complete documentation and straightforward installation are available or ∼30 min for more complex setups; the longest case did not exceed 1 h. This one-time effort amortizes quickly across repeated analyses and users.

Workflow execution begins with the plan agent, which formulates a structured analysis plan based on the user’s objective and retrieved domain knowledge. The task agent then converts each planned step into executable scripts by incorporating detailed usage instructions from execute RAG. These scripts are executed within a Conda-managed environment or a Docker container (supplemental methods: “Docker validation”). The debug agent continuously monitors for runtime failures. When errors occur, it parses diagnostic messages and queries execute RAG for targeted troubleshooting guidance, such as resolving missing dependencies or correcting invalid arguments. To manage context efficiently, the debug agent maintains compact summaries of previous diagnostic attempts, preserving essential information while minimizing token usage. After each step is executed, the check agent verifies the outputs against the planned specifications—ensuring completeness and format conformity. If discrepancies are found, corrective actions are triggered before moving on to the next step. For tools or tasks absent from the curated knowledge base (KB; cold-start scenarios), BioMaster can proceed using the backbone model’s general knowledge; however, this mode is less reliable.

To support coherent inter-agent reasoning, BioMaster performs summarization after every step, recording the task outcome, relevant feedback, and any workflow adjustments. These summaries streamline downstream decisions by providing context in a compact form.

These agents operate in a coordinated loop—planning, executing, validating, and adjusting—until the full workflow is completed. By combining specialized agents with modular retrieval and context management, BioMaster enables robust execution of complex bioinformatics workflows directly from natural language instructions.

To assess real-world usability and mitigate developer-prompt bias, we recruited five participants without bioinformatics expertise (four undergraduates and one master’s student) for a user study. Each participant followed only the tutorial materials referenced in the data and code availability (installation, configuration, and quick-start usage) and was asked to complete two single-cell RNA sequencing (scRNA-seq) tasks—clustering and differential expression—by iteratively formulating natural language goals. scRNA-seq was selected because it yields interpretable visualizations that enable objective evaluation without specialized domain knowledge. We also collected structured user feedback on usability and clarity (supplemental methods: “user case”). Outputs were compared against results produced by an expert. Clustering achieved a median concordance of approximately 0.75, as measured by the adjusted Rand index (ARI) and normalized mutual information (NMI), with uniform manifold approximation and projection (UMAP) structure broadly consistent with the expert solution; differential expression reached roughly 0.85 overlap with expert-selected top genes. Discrepancies arose mainly from parameter choices (e.g., filtering thresholds and clustering resolution) rather than execution failures, indicating that BioMaster can automate pipelines and reduce user effort by shifting attention from workflow execution to parameter refinement.

### BioMaster is a versatile agent for analyzing a wide spectrum of bioinformatics tasks

To assess the versatility of BioMaster, we benchmarked its performance across 49 tasks (Table S2). These workflows encompassed both routine and advanced analyses across genomics, transcriptomics, epigenomics, spatial omics, metagenomics, and proteomics. We compared BioMaster to two baselines: ChatGPT, a general-purpose LLM, and AutoBA, a single-agent pipeline executor. Given the scale of the benchmark, systematic comparison with expert-curated results is impractical. As BioMaster and alternative tools are all designed to automate bioinformatics workflows at the level of tool execution rather than assess biological validity, we evaluate performance by workflow execution success.

**Figure 2:**
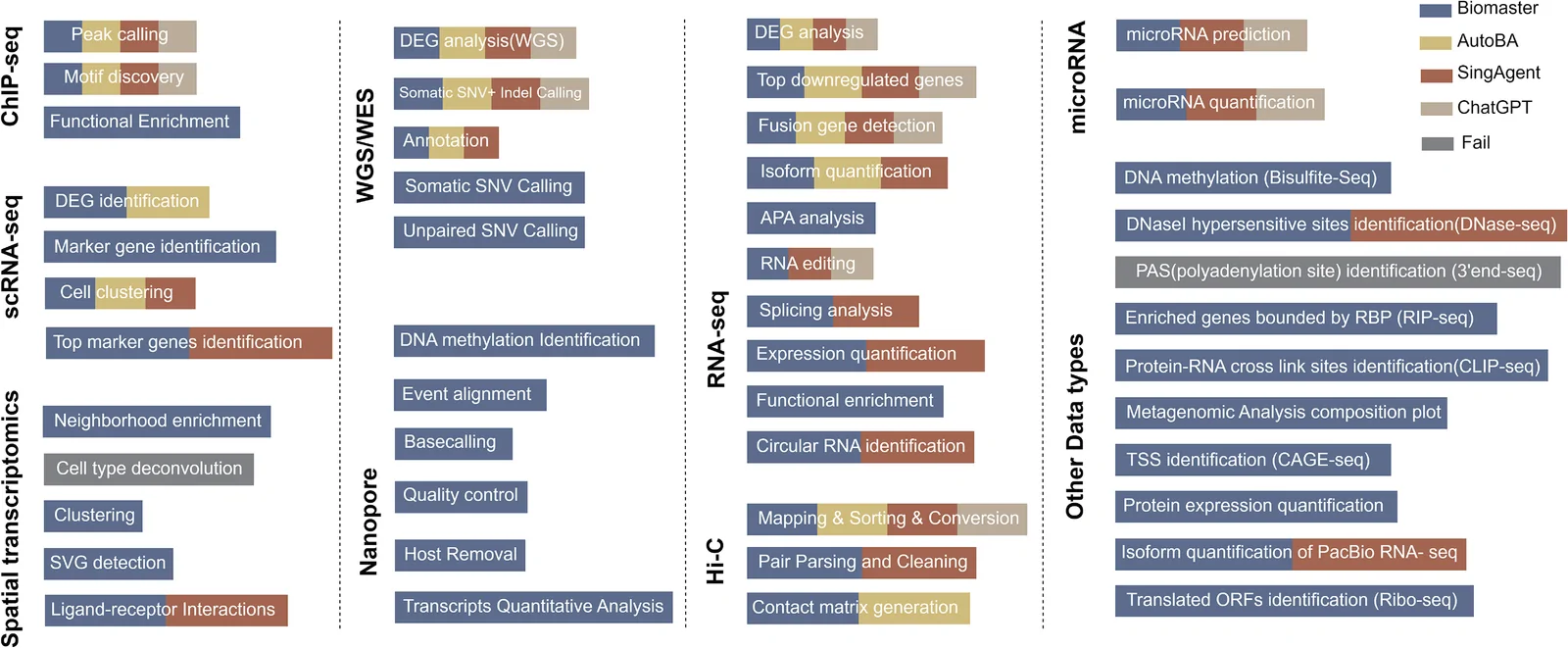
Task coverage of BioMaster, AutoBA, and ChatGPT across bioinformatics workflows Each rectangle represents an analytical task by data type, with colors indicating which system completed it end to end without human intervention. BioMaster shows substantially broader coverage across modalities than AutoBA and ChatGPT.

BioMaster successfully completed 47 out of 49 tasks (95.9%), compared with 24 (49.0%) for SingleAgent, 13 (26.5%) for AutoBA, and 12 (24.5%) for ChatGPT. We configured AutoBA to use the same tool-level execute KB as BioMaster, ensuring consistent access to tool knowledge across methods. The SingleAgent baseline received a single merged KB (plan+execute) to test whether access to the same information—without multi-agent orchestration—closes the gap; ChatGPT ran without an external KB (methods). The SingleAgent baseline improved over the no-KB (without any KB) baseline but remained substantially below BioMaster, with failures concentrated in later, interdependent stages where merged retrieval often surfaced high-level workflow text when fine-grained tool parameters were required and where the absence of specialized debug/check agents limited recovery. Execution logs indicated that 40 workflows invoked the plan RAG module for task-level planning, and 183 of 275 analytical steps accessed the execute RAG module for tool-specific knowledge retrieval. All systems required manual environment setup for R-based packages due to unresolved dependency conflicts—a common challenge in bioinformatics. Despite this shared limitation, BioMaster demonstrated greater robustness by autonomously handling additional technical issues that caused failure in the baseline systems. Figure 2 summarizes completion status across systems, highlighting BioMaster’s consistency across diverse tasks; Table 1 offers a qualitative side-by-side comparison of automation, versatility, error correction, robustness, and token efficiency. To quantify computational overhead, we further analyzed five representative tasks that all systems could complete (single-cell marker identification, clustering, differential expression, chromatin immunoprecipitation [ChIP]-seq peak calling, and motif discovery). We measured total input tokens, output tokens, working time (wall-clock execution), and thinking time (LLM API latency). The detailed results can be found in Figures S6 and S7.

**Table 1:** A comparison of bioinformatics workflow execution methods

Methods	Automation	Versatility	Error correction	Robustness	Token efficiency
BioMaster	✓ ✓ ✓ ✓	✓ ✓ ✓ ✓	✓ ✓ ✓ ✓	✓ ✓ ✓ ✓	✓ ✓ ✓
AutoBA	✓ ✓ ✓	✓ ✓ ✓	✓ ✓ ✓	✓ ✓ ✓	✓
Conventional tools	✓	✓	✓	✓ ✓	–
Online webserver	✓ ✓	✓	✓	✓ ✓	–
ChatGPT	✓ ✓	✓ ✓	✓ ✓	✓ ✓	✓ ✓
Open-source LLMs	✓ ✓	✓	✓	✓	✓ ✓

Each method was evaluated on degree of automation, versatility, error correction, robustness, and token-saving based on qualitative observation of their capabilities.

In standard tasks, such as Hi-C data alignment (Table S6), ChIP-seq peak calling (Table S9), and WGS preprocessing (Tables S22 and S23), all three systems performed comparably. However, as task complexity increased, baseline systems showed a substantial drop in completion rates. For example, in bulk RNA-seq analysis, while all systems handled differential expression (Table S16), only BioMaster consistently completed advanced tasks including alternative polyadenylation analysis (Tables S14 and S15), transcript quantification (Table S32), functional enrichment (Table S33), circular RNA detection (Table S34), and RNA splicing (Table S38). Among these, the alternative polyadenylation workflow highlighted a clear performance gap: BioMaster successfully resolved all dependencies and handled intermediate files correctly, whereas AutoBA failed due to missing annotations, and ChatGPT produced invalid scripts. A similar pattern emerged in splicing analysis: BioMaster generated an executable pipeline end to end, while ChatGPT failed midway through execution, and AutoBA failed to produce essential outputs.

In single-cell transcriptomics, BioMaster was the only system to complete end-to-end workflows, encompassing quality control, clustering, marker gene identification, and differential expression (Tables S50–S59). AutoBA completed clustering but failed in subsequent stages, while ChatGPT consistently failed during preprocessing and initial quality assessment. A similar trend was observed in spatial transcriptomics: BioMaster successfully executed neighborhood enrichment, spatial clustering, and ligand-receptor interaction analysis. In contrast, AutoBA encountered parsing errors (e.g., “key missing” during clustering), and ChatGPT was unable to load spatial data for neighborhood analysis (Tables S43–S49).

In epigenomics, BioMaster completed full Hi-C workflows—from raw read alignment through pair parsing, filtering, and contact matrix generation. AutoBA and ChatGPT completed only the initial mapping and sorting steps, failing in downstream stages that require interdependent tool usage and format-specific handling (Tables S4–S8). These results underscore the strength of BioMaster’s multi-agent system in coordinating complex, tool-specific processes that conventional systems struggle to manage. BioMaster also demonstrated strong performance in long-read sequencing. For Nanopore DNA data, it completed workflows involving base calling, quality control, methylation detection, and event alignment. AutoBA encountered file-handling errors during base calling, whereas ChatGPT generated incorrect methylation detection outputs. Similarly, for long-read transcriptomics from PacBio and Nanopore platforms, BioMaster completed isoform quantification pipelines, whereas AutoBA applied incorrect tools, and ChatGPT failed to parse the input formats (Tables S60–S67).

In specialized RNA-seq modalities such as crosslinking-immunoprecipitation sequencing (CLIP-seq), RNA immunoprecipitation sequencing (RIP-seq), ribosome profiling sequencing (Ribo-seq), genome architecture and gene expression sequencing (GAGE-seq), and cap analysis of gene expression sequencing (CAGE-seq), BioMaster outperformed both baselines. In CLIP-seq, it correctly identified protein-RNA cross-linking sites through a multi-step process, while AutoBA returned an “invalid response” error, and ChatGPT failed to properly use CLIPper (Table S35). In CAGE-seq, BioMaster accurately identified transcription start sites, while both ChatGPT and AutoBA misused inappropriate tools (Table S41). BioMaster also succeeded in complex metagenomic and proteomic analyses. In metagenomics, it performed full microbial community profiling, while both baselines failed to complete the compositional analysis (Table S72). In proteomics, BioMaster executed mass spectrometry-based workflows for protein quantification, overcoming configuration and dependency issues that caused AutoBA and ChatGPT to fail (Table S42).

To further analyze workflow behavior beyond end-to-end completion rates, we examined the procedural quality of the generated plans and scripts. Given the scale of the benchmark and the heterogeneity of tasks, manually inspecting execution artifacts across all workflows would require substantial expert effort and is not feasible in practice. We therefore adopt an automated assessment in which LLMs are used as independent raters to review execution-related properties of workflows, following recent practice in large-scale system evaluation.^25^ Specifically, this assessment was applied to the subset of tasks that all systems could complete (12 tasks; five runs per system). Five LLMs (deepseek_v3_1_250821_thinking, gpt_5_2025_08_07, gemini_2_5_pro_nothinking, gpt_5_2_2025_12_11, and qwen3_max) were used as raters to score the generated plans and scripts along several execution-oriented dimensions, such as plan completeness, script quality, error handling, etc. Scores were aggregated across runs and raters to obtain system-level summaries. As shown in Figure 4A, BioMaster achieves higher scores than alternative systems across these dimensions. The full assessment prompts and per-rater results are provided in Figures S1–S5 and Listing S1. To assess agreement among these raters, we quantified inter-rater reliability using the intraclass correlation coefficient (ICC). Across the seven scoring dimensions, evaluator-averaged reliability was moderate to high, with ICC(3,5) values ranging from 0.70 to 0.92 and higher agreement for more concrete criteria such as task completion (supplemental methods: “multi-rater LLM evaluation protocol”).

In summary, BioMaster demonstrated broad versatility and robustness across a wide range of bioinformatics tasks and data types. Its superior performance stems from its multi-agent architecture, modular task decomposition, and integration of retrieval-augmented domain knowledge—establishing it as a reliable and generalizable framework for automated biological data analysis.

### BioMaster excels across established and emerging bioinformatics tools

Existing methods, including AutoBA and ChatGPT, perform reliably with widely used, well-documented software but often fail when workflows require complex, under-documented, or newly released tools. To assess performance across this spectrum, we benchmarked BioMaster, AutoBA, and ChatGPT on 102 bioinformatics tools spanning established utilities, specialized domain software, and recently developed packages.

For widely used tools with standardized interfaces and comprehensive documentation—such as FastQC,^26^ Trimmomatic,^27^ HISAT2,^28^ Samtools,^29^ and GATK^30^—all three systems generated syntactically valid commands, reflecting the accessibility of well-supported tools to both language model- and template-based systems.

Performance diverged markedly for tools requiring complex parameterization, multi-stage execution, or environment-specific configurations. ChatGPT, although often producing syntactically correct commands, frequently failed due to incomplete arguments, unresolved dependencies, or misinterpreted usage requirements, as with pairtools and cooler for Hi-C processing, phyloseq^31^ for metagenomic classification, and BEDTools^32^ for genomic interval operations. AutoBA performed better for tools with explicit, well-documented workflows but struggled with environment customization or cross-tool integration. Failures included assay for transposase-accessible chromatin using sequencing (ATAC-seq) workflows using BEDTools (incorrect input formatting); genome assembly with minimap2^33^ and dorado (unresolved dependencies); and analyses using bowtie2,^34^ miRDeep2,^35^ or BWA^36^ (Conda installation conflicts). AutoBA also misapplied Trim Galore^37^ in polyadenylation site identification, indicating poor tool-task alignment.

In contrast, BioMaster maintained high success rates across both standard and complex tools. Leveraging its dual RAG framework, it retrieved and parsed official documentation, GitHub repositories, and user-provided resources to generate executable, context-specific commands. In scRNA-seq, it ran pipelines involving Pandas,^38^ Scanpy,^39^ and Cell Ranger,^40^ correctly configuring inputs, clustering parameters, and visualization outputs. In Hi-C analysis, it executed complete pairtools-based workflows with appropriate resolution and normalization, while both baselines failed. In proteomics, it applied Numpy^41^ and Scipy^42^ for mass spectrometry data normalization, avoiding the misapplications seen in AutoBA and ChatGPT. For Nanopore sequencing, it successfully completed the Nanopolish workflow, managing installation, indexing, and event alignment—tasks where both baselines failed due to command-line interface (CLI) errors or dependency mismanagement.

We further tested adaptability to recently released, minimally documented tools: GeneFEAST,^43^ MAFin,^44^ Rnalib,^45^ nichePCA,^46^ and WGATools.^47^ Selected for methodological diversity, recency, and absence from pre-training corpora, these tools challenge static, template-based approaches. BioMaster successfully executed all five by retrieving and parsing source documentation, configuring environments, and generating correct outputs. AutoBA completed two (GeneFEAST and WGATools), while ChatGPT succeeded only on GeneFEAST. Baseline failures stemmed from syntax errors, incorrect parameters, or unresolved dependencies—for example, AutoBA’s inability to configure Rnalib (specialized Conda packages) and failure to run MAFin (incompatible CLI syntax). BioMaster configured Rnalib directly from repository instructions and executed MAFin with multithreaded motif search and correct output formatting.

Collectively, these results demonstrate BioMaster’s ability to operate across the full spectrum of bioinformatics tools. By integrating retrieval-augmented reasoning with dynamic environment configuration, it bridges the gap between static LLM capabilities and the rapidly evolving bioinformatics software ecosystem, enabling autonomous, reproducible analysis across both established and emerging tools.

### BioMaster outperforms alternative systems in multi-step biological data analysis tasks

**Figure 3:**
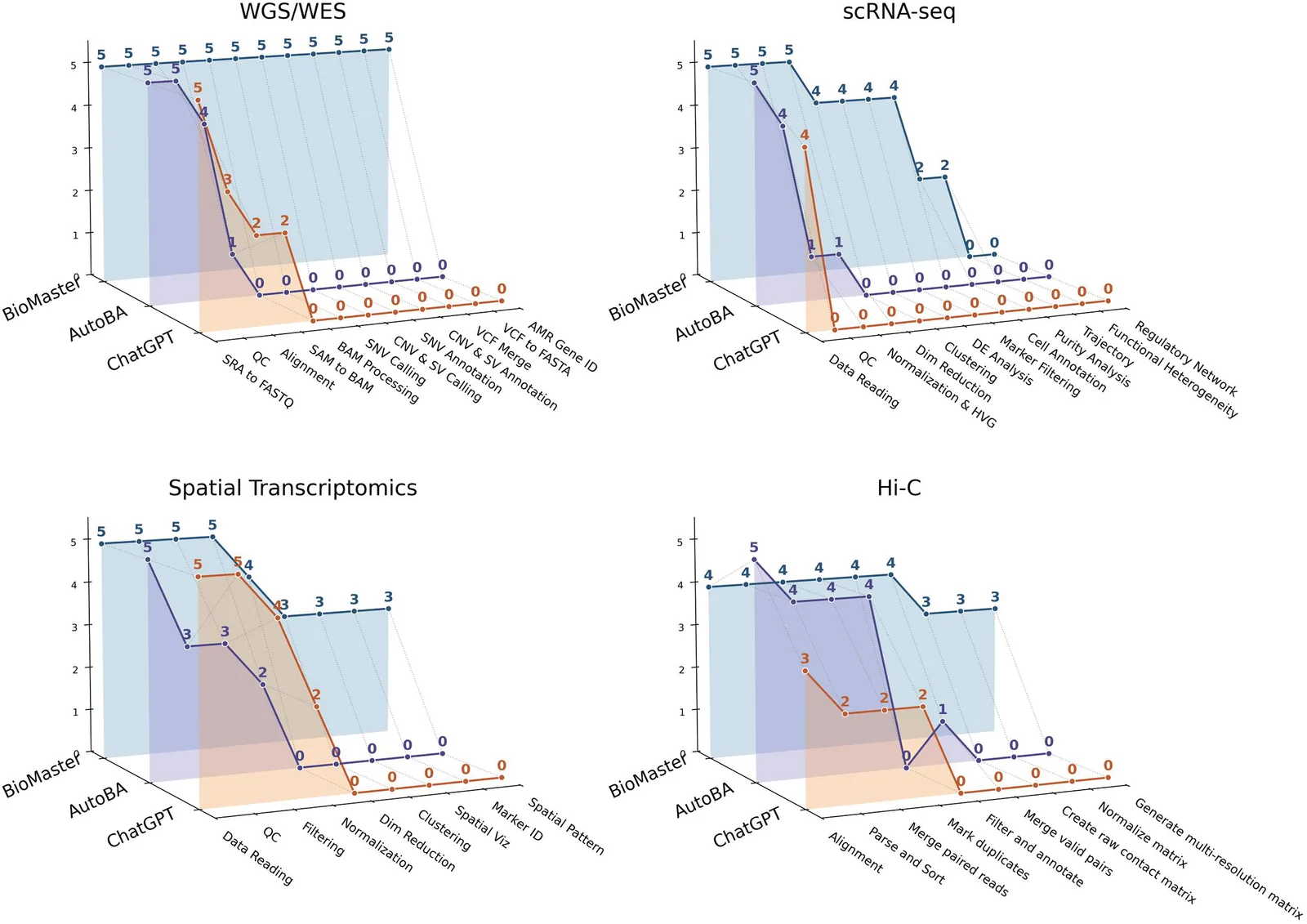
Execution success across multi-step bioinformatics workflows Execution success rates (0–5, across five trials) are shown for BioMaster (blue), AutoBA (purple), and ChatGPT (red) on four representative workflows: Hi-C, single-cell RNA–seq, spatial transcriptomics, and WGS/WES. Each plot tracks the number of successful runs per step, highlighting BioMaster’s higher completion rates across full workflows compared to the alternatives.

We benchmarked BioMaster, AutoBA, and ChatGPT on four computationally intensive workflows—Hi-C chromatin conformation (9 steps), scRNA-seq (10 steps), spatial transcriptomics (9 steps), and WGS/whole-exome sequencing (WES; 12 steps)—each requiring sequential execution of heterogeneous tools, complex file transformations, and dynamic parameter tuning. Each workflow was executed five times per system on identical datasets, with stage-level success (0–5 completions) recorded to assess both performance and reproducibility (Figure 3).

BioMaster achieved the highest completion and reproducibility rates across all workflows, particularly in later stages where tool interoperability and parameter optimization were critical. In Hi-C analysis, it completed all stages in all runs, whereas AutoBA stalled after initial file operations, and ChatGPT failed during matrix construction. In scRNA-seq, BioMaster maintained perfect preprocessing success (5/5) and ≥4/5 in advanced analyses; AutoBA matched early-stage performance but dropped sharply downstream, and ChatGPT failed after quality control. In spatial transcriptomics, only BioMaster consistently progressed through clustering (4/5), spatial visualization (3/5), and spatial expression pattern analysis (3/5), with both baselines failing in these tasks. In WGS/WES, BioMaster completed all 12 stages in all runs, whereas AutoBA and ChatGPT succeeded only in early processing before failing in variant annotation and interpretation.

BioMaster also supports user intervention at any stage of execution without restarting workflows, allowing parameters or tools to be modified and execution to resume from the same point. This flexibility, absent in alternatives, reduces redundant computation and makes BioMaster well suited for iterative and exploratory analyses.

### BioMaster enables autonomous error correction in complex bioinformatics workflows

**Figure 4:**
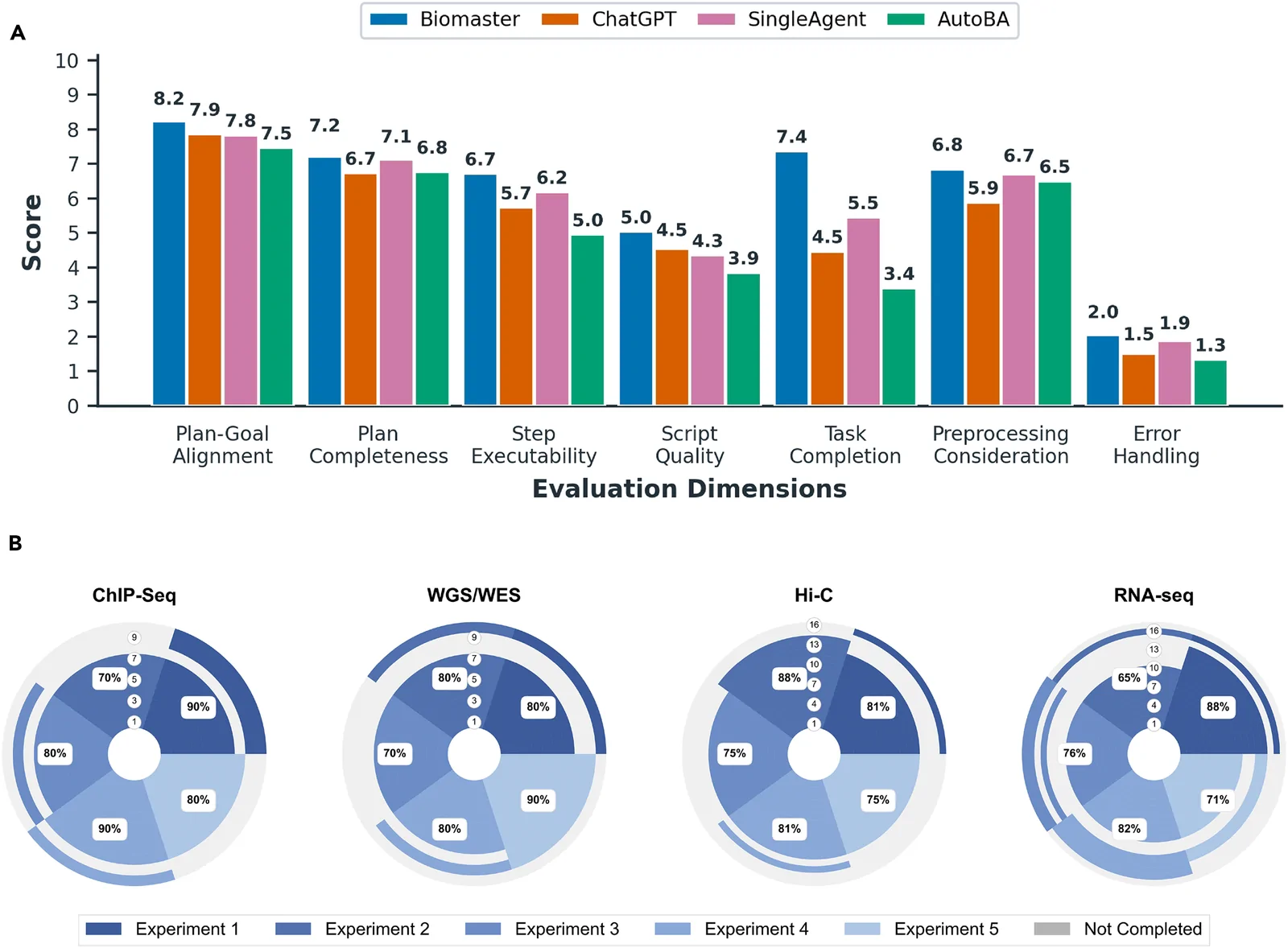
Evaluation and ablation analysis of agentic bioinformatics systems (A) Comparison of four agentic systems (BioMaster, ChatGPT, SingleAgent, and AutoBA) under a unified evaluation protocol. From the pool of tasks that all four systems can successfully execute end to end, we uniformly sampled 12 tasks. Five mainstream LLMs served as independent raters and scored each system’s outputs on seven dimensions (plan-goal alignment, plan completeness, step executability, script quality, task completion, preprocessing considerations, and error handling) on a 1–10 scale. For each system and dimension, scores were first averaged over the sampled tasks for each rater and then averaged across the five raters to obtain the final mean shown here. (B) Ablation analysis of BioMaster’s debug agent across four bioinformatics workflows. Each pie chart represents a distinct workflow type, with segments showing the proportion of successful completions for each experiment (darker blue indicates earlier experiments; gray denotes incomplete steps). Numbers on the ring indicate the number of analytical steps in each workflow. Percentages within segments reflect completion rates for individual experiments.

We evaluated the impact of BioMaster’s debug agent—which works with the execute RAG module to detect, diagnose, and recover from execution errors—by comparing BioMaster (referred to as the full system) to a variant without this debug agent. Four representative workflows were tested—ChIP-seq peak calling, somatic variant detection in WGS/WES, Hi-C contact matrix construction, and RNA-seq differential expression—each executed five times, with success recorded at each analytical step (Figure 4B). Across all workflows, the debug agent substantially increased completion rates by preventing early-stage errors from propagating downstream (Table S3). In ChIP-seq, the full system completed all five stages in every run, while the ablated version failed in peak calling due to uncorrected trimming and genome indexing errors. In WGS/WES, the debug agent enabled complete preprocessing and achieved 80% success in variant calling, compared to 40% alignment success and no variant calling without it. In Hi-C, the debug agent improved stepwise success rates to 60%–80%, completing fragment parsing, contact matrix generation, and normalization, whereas the ablated system failed at nearly all steps. In RNA-seq differential expression, the full system completed all runs end to end, whereas the ablated version failed before quantification in all trials. Mechanistically, the debug agent intercepts runtime errors, parses diagnostics, retrieves tool-specific remediation strategies from execute RAG, and iteratively refines and re-executes failed steps. These results establish the debug agent as essential for maintaining workflow resilience and achieving uninterrupted execution in complex, error-prone bioinformatics pipelines.

To further assess the final outputs of BioMaster, we selected representative tasks—including Hi-C contact matrix construction, scRNA-seq clustering, spatial transcriptomic domain identification, and metagenomic community profiling—and reanalyzed the same raw datasets using manually implemented workflows. We then compared the outputs generated by BioMaster with those obtained from these manual analyses.

**Figure 5:**
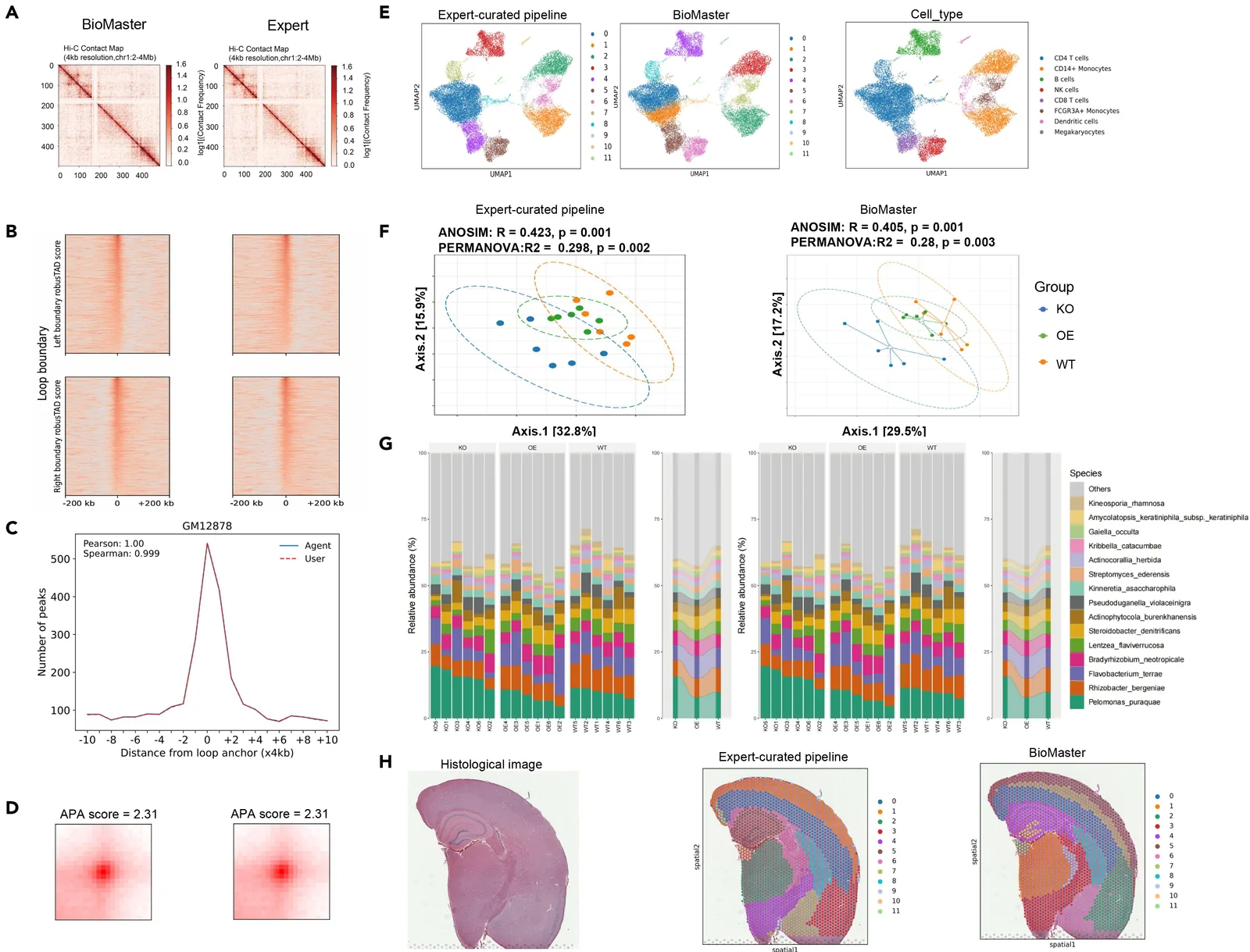
Comparison of results produced by BioMaster and manually curated pipelines across four representative multi-omics workflows (A) Hi-C contact matrices of chromosome 12 from GM12878 cells generated by BioMaster and manually curated pipelines. (B) Loop pile-up heatmaps centered at loop boundaries. (C) CTCF signal enrichment profiles at loop anchors. The two results are highly similar, resulting in nearly identical plots (Pearson *r* = 1.00, Spearman *ρ* = 0.999). (D) Aggregate peak analysis (APA) heatmaps with identical scores. (E) UMAP projections of PBMC single-cell RNA-seq data showing clustering results from manually curated pipelines, BioMaster, and annotated cell types. (F and G) Principal coordinates analysis (PCoA) and relative taxonomic composition of *Arabidopsis* root microbiome samples based on whole-genome shotgun sequencing. (H) Spatial transcriptomic domain segmentation of mouse brain tissue from a histological image, manually curated pipeline, and BioMaster.

In Hi-C analysis of GM12878 data, BioMaster’s contact matrices were visually indistinguishable from manually produced outputs (Figure 5A), with stratum-adjusted correlation coefficient (SCC) values from HiCRep^48^ exceeding 0.99 across all chromosomes (Figure 5B). Loop detection with Mustache^49^ produced identical loop counts and comparable CTCF enrichment (Figure 5C), and aggregate peak analysis scores matched exactly (APA = 2.31; Figure 5D). In scRNA-seq analysis of a 10× Genomics peripheral blood mononuclear cell (PBMC) dataset, both BioMaster and manually produced workflows identified 12 transcriptionally distinct clusters with concordant UMAP embeddings (Figure 5E) and consistent recovery of major immune cell types. Minor differences in cluster granularity reflected alternative yet biologically plausible interpretations. In metagenomic profiling of *Arabidopsis* root microbiomes, BioMaster and manually produced pipelines generated highly concordant taxonomic profiles, with principal coordinates analysis (PCoA) showing nearly identical group separation (Figures 5F and 7G) and comparable analysis of similarities (ANOSIM; R = 0.405 vs. 0.423) and permutational multivariate analysis of variance (PERMANOVA; R*2* = 0.280 vs. 0.298) statistics. Both identified the same key taxa with similar abundance distributions. In spatial transcriptomics of mouse brain tissue (10× Visium), both workflows produced 12 spatial domains, but BioMaster’s segmentation aligned more precisely with known anatomy, particularly in differentiating cortical and subcortical regions (Figure 5H).

These results indicate that BioMaster can produce outputs comparable to manually implemented workflows across the evaluated tasks. While these results are encouraging, they are limited to the analyses considered here, and broader evaluation across additional tasks and datasets will be necessary to fully assess the generality of this behavior.

### BioMaster adapts to open-source LLMs

We then studied how BioMaster performs with open-source LLMs. In this evaluation, we tested GPT-oss-120B, Qwen3-235B, Qwen3-30B, DeepSeek-R1, and Kimi-K2 and carried out 26 tasks spanning genomics, transcriptomics, epigenomics, spatial omics, and metagenomics. This design allowed us to assess whether BioMaster can operate effectively without relying on proprietary models. We found that BioMaster maintained high task-level success rates across all tested open-source backends (Table 2), demonstrating that the framework is model agnostic and robust in fully open deployments.

**Table 2:** Performance of BioMaster across diverse bioinformatics tasks using different open-source large language models

Data type	Task	GPT-120b	Qwen3-235b	Qwen3-30b	Kimi-K2	Deepseek-R1
ChIP-seq	peak calling	✓	✓	✓	✓	✓
ChIP-seq	motif discovery	✓	✗	✗	✓	✓
ChIP-seq	functional enrichment	✗	✓	✗	✓	✓
WGS/WES	somatic structural variation (SV) calling	✓	✗	✗	✓	✗
WGS/WES	somatic SNV + indel calling	✓	✓	✓	✗	✓
WGS/WES	DEG analysis (WGS)	✓	✓	✗	✗	✓
scRNA-seq	DEG identification	✓	✓	✗	✓	✓
scRNA-seq	marker gene identification	✓	✓	✓	✓	✓
scRNA-seq	cell clustering	✓	✓	✓	✓	✓
RNA-seq	RNA editing	✓	✓	✓	✓	✓
RNA-seq	fusion gene detection	✓	✓	✗	✓	✓
RNA-seq	DEG analysis	✓	✓	✗	✓	✓
RNA-seq	top downregulated genes	✓	✗	✗	✗	✓
RNA-seq	isoform quantification	✗	✓	✗	✗	✓
ST	neighborhood enrichment	✓	✓	✗	✗	✗
ST	clustering	✓	✓	✓	✓	✓
ST	ligand-receptor interactions	✓	✓	✗	✓	✓
Hi-C	mapping, sorting, and conversion	✓	✓	✓	✓	✓
Hi-C	pair parsing and cleaning	✓	✓	✗	✓	✓
Hi-C	contact matrix generation	✓	✓	✗	✓	✓
Other types	DNase I hypersensitive sites (DNase-seq)	✓	✓	✓	✗	✓
Other types	RIP-seq (RNA-binding protein bound enriched genes)	✓	✓	✗	✓	✓
Other types	CLIP-seq (protein-RNA cross-link sites)	✓	✓	✓	✓	✓
Other types	TSS identification (GAGE-seq)	✓	✓	✗	✗	✓
Other types	PCA-based spatial domain (NichePCA)	✓	✓	✗	✓	✓
Other types	MAF motif via regex/K-mers/position weight matrices (MAFin)	✓	✓	✓	✓	✓

The evaluation was conducted on 26 representative tasks spanning multiple omics data types, powered by five distinct open-source LLMs. A checkmark indicates that the model successfully guided BioMaster to complete the entire workflow for the given task, while an ✗ signifies a failure. indel, insertion or deletion; DEG, differentially expressed gene; ST, spatial transcriptomics; TSS, transcription start site; PCA, principal component analysis; MAF, minor-allele frequency.

Among the tested backbones, Qwen3-235B and DeepSeek-R1 were explicitly designed with reasoning capabilities, while GPT-oss-120B and Kimi-K2 primarily followed instruction-tuning paradigms. The reasoning-oriented models exhibited the most stable execution across multi-stage pipelines, reliably handling cross-step dependencies, parameter adaptation, and inconsistent identifier formats (e.g., mixed Entrez IDs and gene symbols). GPT-oss-120B achieved performance broadly comparable to these reasoning-oriented backbones, despite being optimized mainly for instruction following. By contrast, Qwen3-30B occasionally produced incomplete runs, while Kimi-K2 required more detailed goal specifications in the prompts to produce reliable results. These differences were most pronounced in long workflows that require maintaining implicit assumptions across multiple steps, such as consistent file formats, identifier conventions, and tool-specific output contracts.

To better characterize open-source model behavior, particularly the limitations of smaller LLM backbones in complex, long workflows, we examined representative failure patterns observed with a 30-billion (30B)-scale open-source model (Qwen3-30B). In alternative polyadenylation quantification in mouse RNA-seq data, the smaller model entered repeated debug loops when attempting to install a non-existent or private repository, resulting in persistent installation errors (e.g., repository not found or authentication failures) without proposing a viable alternative for 3′ end extraction. In Hi-C processing (e.g., pairtools parse), the smaller model produced an empty or invalid intermediate file in an upstream step and initially misattributed the resulting downstream exception to missing dependencies before identifying the actual cause—a missing or malformed pairs header—leading to cascading failures in subsequent steps. In scRNA-seq marker discovery and visualization with Scanpy, the smaller model completed upstream preprocessing and clustering but repeatedly failed during downstream visualization due to brittle plotting defaults (e.g., oversized figures exceeding Matplotlib limits) and hard-coded column-name assumptions. Similar patterns were observed in cell clustering and marker gene identification, where shortened workflows omitted key analysis steps, resulting in incomplete outputs. Occasional failures also arose from fragile parameterization, such as a misplaced newline before end-of-file (EOF) in a bash heredoc block that caused delimiter parsing failures. For larger models, such as GPT-oss-120B, we observed in functional enrichment that GPT-oss-120B occasionally encountered input inconsistencies when a script assumed the gene list contained only Entrez IDs, while the actual list included a mixture of gene symbols and cross-species identifiers. Importantly, BioMaster reliably prevented catastrophic failures such as empty or invalid outputs, ensuring that each step produced syntactically valid results; however, it could not always enforce that outputs were formatted in ways suitable for downstream tasks. Addressing such issues depended on the underlying model’s ability to reason about cross-step constraints, data formats, and implicit assumptions embedded in downstream tools. We also observed that reasoning-oriented models such as Qwen3-235B and DeepSeek-R1 handled these cases more comprehensively, with failures largely restricted to fine-grained parameter settings, whereas smaller or instruction-only backbones exhibited more frequent breakdowns in cross-step consistency.

Although BioMaster ensured that each individual task produced syntactically valid and non-empty outputs, downstream dependencies could still be affected if intermediate results did not conform to the semantic or format requirements of subsequent steps. For example, a functional enrichment script assumed that the input gene list contained only Entrez identifiers, which led to a mapping failure when the preceding step instead returned a mixture of gene symbols and mouse/human IDs. In such cases, the workflow technically progressed through all steps, but the scientific validity of the final output was compromised by format mismatches or incomplete normalization.

Overall, these findings demonstrate that small- and mid-scale open-source models can sustain routine analyses, while GPT-oss-120B, Qwen3-235B, and DeepSeek-R1 more reliably support complex, long-horizon pipelines through complementary mechanisms—strong instruction following in the case of GPT-oss-120B and explicit reasoning in the case of Qwen3-235B and DeepSeek-R1. Together, these results confirm BioMaster’s model-agnostic reproducibility and provide a practical foundation for sustainable, fully open-source deployments in computational biology. At the same time, the choice of foundational LLM remains a principal determinant of the ceiling performance achievable under an identical tool-level KB.

## Discussion

The increasing scale and heterogeneity of biological data have made bioinformatics workflows difficult to construct and maintain, often requiring coordination across many tools, file formats, and software environments. This motivates automation that reduces engineering overhead while preserving flexibility for expert-driven analysis. In this study, we introduced BioMaster, an LLM-enabled multi-agent framework for automating complex bioinformatics workflows through coordinated planning and execution.

BioMaster is designed to improve the reliability of long, interdependent pipelines by combining a multi-agent execution framework with a curated tool- and pipeline-level KB. Together, these components enable orchestration of tool invocation, dependency management, and recovery from common execution failures, increasing the chance that complex workflows complete successfully. Ablation results show that this design improves workflow completion rates and recovery from execution errors in multi-step analyses.

Within this execution-centric design, the debug agent plays a focused role in improving pipeline stability by handling explicit execution failures. Specifically, it addresses errors that are directly signaled at runtime, such as non-zero exit codes, recognizable error patterns in logs or standard error streams, and missing or empty outputs. It does not attempt to diagnose data-specific issues, such as malformed biological inputs or unexpected data distributions. Such issues often lack clear execution-level signals and typically require domain expertise and contextual judgment, making them challenging even for experienced human experts. BioMaster therefore concentrates on stabilizing software execution while leaving data-level interpretation and analytical decision-making to the user.

This focus on execution also determines how BioMaster interacts with biological data. Rather than processing raw data—such as nucleotide sequences or large genomic files—directly within the LLM context, BioMaster delegates data handling to specialized external bioinformatics tools. The LLM agents operate on workflow structure, parameters, file paths, and execution feedback, allowing the system to avoid challenges related to LLM context limits and biological tokenization while leveraging established domain software for data analysis.

BioMaster is designed to be model agnostic and can operate with both proprietary and open-source LLM backbones. This architectural choice allows users to balance cost, accessibility, and performance according to available resources while keeping the underlying tool- and pipeline-level knowledge constant across models.

Within this model-agnostic setting, model choice nonetheless influences practical performance. In our experiments, models with 30 B or even fewer parameters reliably supported short tasks (e.g., single-tool or shallow multi-step analyses) but struggled with long pipelines. These more complex workflows benefited from larger or reasoning-optimized backbones (approximately 70–120 B+), with GPT-oss-120B achieving high success across most tasks under identical tool- and pipeline-level knowledge. Importantly, BioMaster’s performance gains over alternative systems persist when both the underlying LLM and the tool knowledge are held constant, indicating that improvements do not arise solely from model scale.

Despite its strengths, BioMaster has several limitations. It lacks native integration with high-performance computing (HPC) schedulers, which can complicate deployment on clusters with restricted network access. The system is designed to assist rather than replace expert bioinformaticians and therefore focuses on execution-level robustness; detecting subtle analytical or biological errors remains outside its scope. In addition, BioMaster assumes standard input and output formats, and tools with highly customized interfaces may require additional knowledge entries. As with other bioinformatics agents, procedural execution success should not be conflated with scientific correctness, which remains dependent on expert judgment.

In summary, BioMaster demonstrates that multi-agent LLM frameworks can automate complex bioinformatics workflows using modular reasoning and structured retrieval, suggesting a scalable path for tool-driven bioinformatics automation.

## Methods

### System environment

All experiments used OpenAI’s o1-2024-12-17 (OpenAI, “o1”)^50^ as the underlying LLM for all agents. BioMaster was deployed on both local and cloud servers to assess performance across computing environments. The cloud server was equipped with an AMD EPYC 7643 48-core processor and 756 GB of RAM. The local server featured a 13th Gen Intel Core i9-13900K CPU and 96 GB of RAM. For optimal performance, a CPU with ≥16 GB of RAM is recommended. The software stack ran on Ubuntu 22.04 with Conda as the primary package and environment manager. Python (v.3.12) and R (v.4.4.1) environments were configured via Conda for compatibility with required bioinformatics tools and libraries. BioMaster was developed using the LangChain framework for multi-agent orchestration.

### Experiment setting

We compared BioMaster with two baselines: AutoBA and a ChatGPT-based single-agent pipeline. AutoBA was installed and executed following the procedures described in its public GitHub repository; default settings were used without modification to the source code. To ensure knowledge parity, both BioMaster and AutoBA received the same tool-centric execute KB (command-line syntax, parameters, installation, and troubleshooting); only a format conversion was applied to meet AutoBA’s RAG ingestion requirements. By contrast, BioMaster also employs a planning KB (Plan-KB) consulted by its plan agent; AutoBA does not expose a Plan-KB interface. All systems were evaluated on the same datasets and under identical computational environments. For the ChatGPT-based baseline, we used the same high-level prompts as in BioMaster but without access to an external KB. Workflow execution was divided into two phases: (1) generation of a complete analysis plan and (2) stepwise execution of the plan. For each step, ChatGPT generated the execution script, ran the script, and reviewed the outputs and logs to determine whether to proceed or attempt debugging. The maximum number of debugging iterations was set to five per task, matching the configuration used for BioMaster and AutoBA. All systems operated with deterministic generation settings and identical execution privileges, environment constraints, and timeout policies.

Two complementary evaluation tracks were implemented. In the task coverage track, 49 bioinformatics tasks were curated. Each system was allowed up to five independent attempts per task. A task was considered successfully covered if at least one attempt met all of the following criteria: a bioinformatician verified that the workflow was appropriate for the intended analysis, valid outputs were produced for all workflow steps, all declared output files existed and were non-empty, and the relevant software ran to completion without errors. This track evaluated procedural completion only and did not assess the analytical accuracy of the results.

In the multi-step biological data analysis track, a subset of complex workflows was selected, each associated with an ordered list of essential analysis steps, including quality control, read alignment, quantification, model fitting, and post-processing. Although systems could merge or split steps internally, a standardized mapping procedure was applied to align all executions with the step list for fair comparison. For each workflow, five independent attempts were conducted. Progress was recorded as the highest canonical step completed before all debugging attempts were exhausted, invalid outputs prevented further execution, or a timeout occurred.

In addition, modality-specific evaluation metrics were used to compare BioMaster with alternatives. For Hi-C analysis, reproducibility of contact matrices was quantified using the SCC implemented in HiCRep,^48^ which measures the similarity of contact frequency patterns across genomic distances; values close to 1 indicate highly concordant maps. Loop detection results from Mustache^49^ were compared by total loop counts and by CTCF enrichment at loop anchors, and aggregate peak analysis (APA) scores were used to assess the focal enrichment of interaction signals. For scRNA-seq, cluster structures were compared using 2D UMAP embeddings and concordance of major cell-type assignments relative to alternative annotations. For metagenomic community profiling, Bray-Curtis dissimilarities between samples were visualized using PCoA, and group separation was quantified using ANOSIM and PERMANOVA, both of which test whether community composition differs significantly between groups. For spatial transcriptomics, domain segmentation was evaluated qualitatively by alignment with known anatomical regions, with particular attention to boundaries between cortical and subcortical structures.

### RAG

The RAG system in BioMaster consists of two independent KBs: plan RAG for workflow design and execute RAG for execution and troubleshooting. Both are implemented as vector databases storing structured knowledge entries in a predefined JSON schema. Each JSON record contains standardized fields describing either a workflow (methodological steps, objectives, tools, inputs, and outputs) or a tool (parameters, command-line syntax, configuration examples, and troubleshooting guidance).

Users can add new knowledge in two ways. First, they may provide JSON records directly, ensuring immediate compatibility with the database schema. Alternatively, they can supply unstructured sources such as URLs or PDF files. In this case, BioMaster uses an LLM-based ingestion pipeline to extract relevant content, summarize it, and convert it into the standard JSON format. Once generated or provided, JSON records are embedded into high-dimensional vectors using the OpenAI text-embedding-ada-002 model and stored in the vector database with associated metadata. This indexing enables semantic retrieval during workflow execution.

When multiple valid tools are available for a step, BioMaster applies a hierarchical policy: user-specified tools in the initial prompt take precedence. In the absence of explicit user preferences, the plan agent consults plan RAG and, where a curated workflow exists for the task, adopts its tool choices. If no suitable workflow is retrieved, the agent consults execute RAG to surface plausible candidates. When neither KB yields a decisive option, the planner selects a tool based on the underlying LLM’s general knowledge; this fallback is inherently non-deterministic and therefore not directly controllable. When several tools appear similarly appropriate, the planner records a small set of viable alternatives along with their preconditions. During execution, if a debugging cycle cannot restore progress, the system attempts a controlled switch to the next compatible alternative without full re-planning.

Curation proceeds through the addition and lightweight revision of JSON source records. Users may add, edit, or delete entries by modifying the underlying JSON files; BioMaster monitors these changes and incrementally re-ingests the affected records, updating embeddings and metadata in the vector store. The schema supports provenance fields (e.g., source identifier/URL, retrieval date, and optional revision tag) to document origin and recency. This mechanism accommodates both curated JSON inputs and content ingested from unstructured sources while keeping the retrieval corpus synchronized with user edits.

For reproducibility, we provide the exact snapshot of the KBs used in this study (plan RAG and execute RAG) as versioned JSON dumps in our public repository, together with scripts to rebuild the vector indices. This snapshot constitutes the definitive reference for the reported experiments. Downstream users can branch or tag their own snapshots as they extend or customize the KBs for specific applications.

### Memory management strategy

BioMaster implements a summarization-based memory mechanism, inspired by mem0,^51^ to manage context efficiently in long, multi-stage bioinformatics workflows. Instead of retaining complete outputs from all previous steps, the system stores only condensed and relevant information required for error recovery. For each step, the retained context includes the executed script from the previous iteration, a structured summary of any failures (such as missing output files, incorrect parameters, or absent target directories), and a brief record of identified error causes when available. In the case of an unsuccessful execution, the system creates a structured debug record that contains an explicit success/failure flag, a diagnostic summary of the issue, and the modified script used for re-execution. This design ensures that subsequent steps can access essential troubleshooting information without reloading full historical logs.

**Figure 6:**
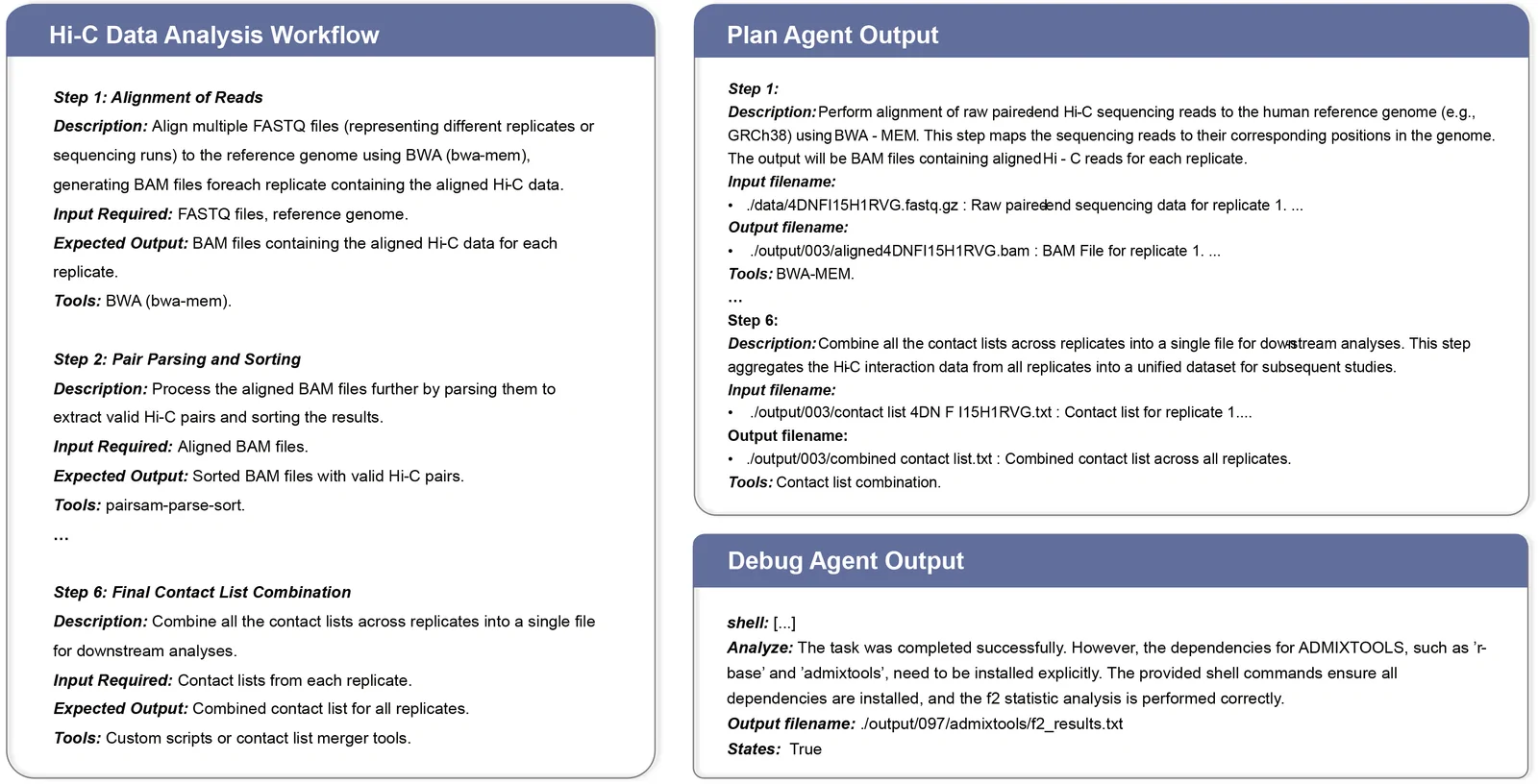
Example knowledge and LLM-generated outputs from BioMaster agents Example Hi-C workflow entry from plan RAG (left), LLM-generated executable plan from the plan agent (top right), and LLM-generated diagnostic report from the debug agent (bottom right).

The task description for the current step is always included in the retained context, ensuring that the intended goal remains clear during multi-round debugging and preventing drift from the original objective. As illustrated in Figure 6, this summarization-based representation reduces token usage, focuses the LLM on diagnostically relevant content, and maintains an efficient, goal-oriented context for error resolution. This approach improves the robustness and efficiency of debugging in complex bioinformatics workflows while avoiding the limitations of full-history retention.

### Agents

#### Plan agent

The plan agent generates structured workflows by decomposing a user-defined analysis goal into a sequence of actionable steps. It queries the plan RAG KB to retrieve relevant information, including workflow-level information such as objectives, tools, input specifications, and expected outputs. Retrieved records are selected based on semantic similarity between the user request and the stored embeddings, enabling the agent to incorporate contextually relevant procedures into the plan.

The plan agent merges the retrieved methodological information with the user’s request to produce a complete workflow specification. Each step in the plan defines the tool to be used, input file descriptions and locations, and the expected outputs. The final workflow is output as a JSON object that conforms to BioMaster’s internal schema, ensuring direct compatibility with downstream agents for execution, error handling, and validation.

#### Task and debug agents

The task and debug agents work together during the execution phase of BioMaster. The task agent receives each workflow step from the plan agent and generates an executable command or script. To ensure correct syntax and parameterization, it queries the execute RAG KB for tool-specific details such as parameter options, configuration examples, and usage patterns. The generated command is tailored to the step’s requirements and the overall workflow context, then executed on the target system.

Following execution, the task agent passes the command, execution logs, and resulting outputs to the debug agent. The debug agent verifies whether the step completed successfully. If an error is detected, it retrieves troubleshooting strategies from execute RAG, analyzes the error message, and modifies the command accordingly. The corrected command is re-executed, and this process is repeated until the step succeeds or all recovery options are exhausted.

To efficiently manage long workflows, the debug agent uses LLM-based summarization to condense diagnostic history into concise records. This reduces memory usage while retaining essential context for subsequent error analysis and resolution. Once a step is successfully completed, the debug agent forwards the validated outputs to the check agent for further verification.

This modular, iterative collaboration between the task and debug agents ensures that each workflow step is executed with precision and that errors are addressed immediately, preventing disruptions to downstream tasks.

#### Check agent

The check agent verifies outputs after each execution step to detect and correct errors before they affect downstream tasks. It compares the outputs generated by the debug agent against the specifications in the workflow plan, confirming that all expected files exist in the designated locations, are non-empty, and match the required formats and naming conventions.

If a validation check fails, the step is marked as unsuccessful, and the issue is reported back to the debug agent for correction. In cases where discrepancies arise—such as mismatched file formats, incorrect naming, or missing intermediate results—the check agent may also update the workflow plan to maintain compatibility with subsequent steps.

By validating after every step and feeding corrections back into the execution loop, the check agent prevents small inconsistencies from propagating, ensuring the workflow remains aligned with the planned specifications and proceeds reliably to completion.

### BioMaster implementation details

**Figure 7:**
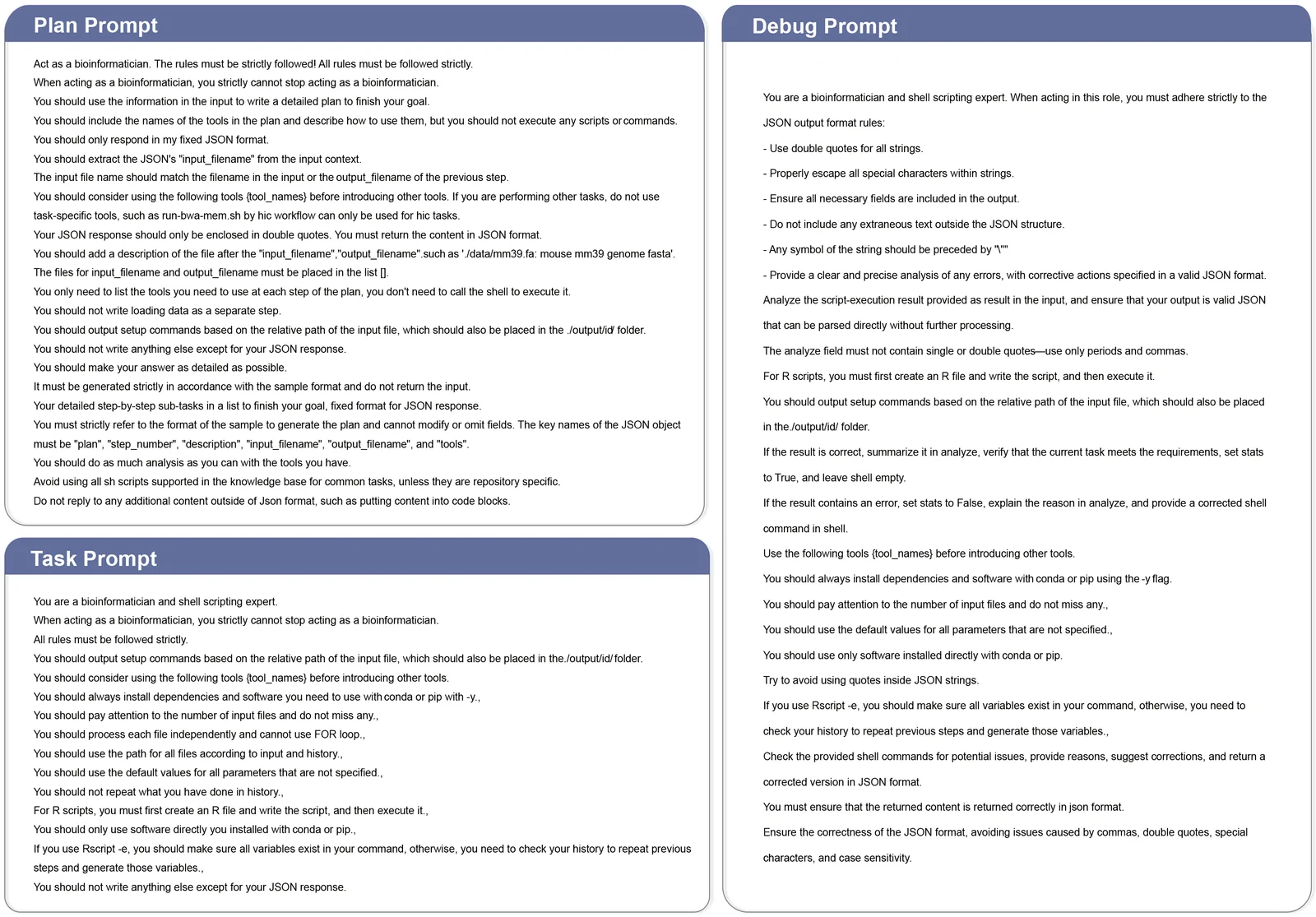
Prompts of BioMaster agents

BioMaster is an open-source Python system available at https://github.com/ai4nucleome/BioMaster. The framework is written in Python and built on LangChain for multi-agent orchestration, with Chroma serving as the vector database for RAG. Each agent is associated with a corresponding prompt, as illustrated in Figure 7. Workflows are expressed in natural language and decomposed into executable subtasks by BioMaster’s agents. All models and embedding backends can be configured through a unified configuration file, supporting both local and API-based deployments.

For the primary experimental setting, BioMaster was evaluated using the proprietary reasoning model o1-2024-12-17 (OpenAI, “o1”)^50^ together with the embedding model text-embedding-ada-002.^52^ This configuration was adopted for the majority of experiments, including direct comparisons with ChatGPT and AutoBA baselines, ensuring consistency across LLM and embedding layers.

To assess generalizability to fully open-source backbones, we further tested BioMaster with a diverse set of publicly available LLMs: GPT-oss-120b (GPT-120b),^53^ Qwen3-235B-A22B-Thinking-2507 (Qwen3-235b),^54^ Qwen3-30B-A3B-Thinking-2507 (Qwen3-30b),^54^ Kimi-K2-Instruct (Kimi-K2),^55^ and DeepSeek-R1-0528 (DeepSeek-R1).^56^ Two deployment modes were used. For models supported by Ollama (https://ollama.com/), such as GPT-oss-120b and the embedding model bge-m3,^57^ model weights were pulled locally and executed on a workstation equipped with an Intel i9-13900K CPU (96 GB of RAM) and a single NVIDIA RTX 4090 GPU (24 GB of VRAM). Models approaching 100 B parameters generally required effective memory availability above 80 GB; partial CPU offloading was used when GPU capacity was insufficient.

For larger backbones that are not feasible to host locally, such as Qwen3-235B and DeepSeek-R1, we accessed them via API endpoints provided either directly by the model developers (official APIs) or by third-party services such as the SiliconFlow platform (https://www.siliconflow.com). Embedding models were likewise invoked via APIs when local deployment was not practical. Switching between local Ollama deployments and API calls required no code modifications, as BioMaster provides a unified configuration file that flexibly specifies model providers, task assignment, and retrieval settings. All API-based evaluations were performed under the default service quotas, without fine-tuning or custom modifications of the remote models.

### Ethics statement

The study was approved by the Hong Kong University of Science and Technology (Guangzhou) (approval no. HKUST(GZ)-HSP-2026-0016). The study was conducted in accordance with institutional ethical standards, and all participants provided informed consent before participation.

## Resource availability

### Lead contact

Requests for further information and resources should be directed to and will be fulfilled by the lead contact, Yanlin Zhang (yanlinzhang@hkust-gz.edu.cn).

### Materials availability

This study did not generate any physical materials.

### Data and code availability

• The datasets analyzed in this study are listed in Table S2. All source code, scripts, and data for the experiments reported in this paper have been published on Zenodo.^58^
• BioMaster source code, documentation, and related scripts are publicly available at https://github.com/ai4nucleome/BioMaster under the MIT License.

## Acknowledgments

This work is supported by the 10.13039/501100001809National Natural Science Foundation of China (no. 32500550), the Guangzhou-HKUST(GZ) Joint Funding Program (2025A03J3865), and a Guangdong Provincial Project (2024QN11N085).

## Author contributions

H.S., J.F., and Y.Z. conceived the study. H.S., W.L., Y.L., Y.X., Jinming Yang, H.L., Jixin Yang, X.Y., S.X., C.W., Y.H., and T.Z. performed the analysis. Y.Z. supervised the project. H.S., J.F., and Y.Z. wrote the article. All authors read and approved the final article.

## Declaration of interests

The authors declare no competing interests.

## Declaration of generative AI and AI-assisted technologies in the writing process

During the preparation of this work, the authors used ChatGPT to polish the writing. After using this tool or service, the authors reviewed and edited the content as needed and take full responsibility for the content of the publication.

## Supplemental information

Document S1. Figures S1–S7, Tables S1–S72, Listing S1, and supplemental methods

Document S2. Article plus supplemental information
