# Supplementary material for: BioMaster: Multi-agent system for automated bioinformatics analysis workflow
Source: Patterns (N Y). 2026 Jul 7;7(8):101611. doi: 10.1016/j.patter.2026.101611 (PMC13494596; doi:10.1016/j.patter.2026.101611)
Supplement: Supplementary file 1 [file mmc1.pdf]

Patterns, Volume *7*

Supplemental information

BioMaster: Multi-agent system

for automated bioinformatics analysis workflow

Houcheng Su, Junning Feng, Yawen Lu, Yucheng Xu, Jinming Yang, Haojie Lu, Jixin Yang, Xu Yang, Sirui Xie, Weicai Long, Chengrui Wang, Yusen Hou, Tingyu Zhu, and Yanlin Zhang

Supplemental Information Overview

This supplemental Information provides supporting materials for the evaluation of BioMaster and alternative tools on 49 bioinformatics analysis tasks. In addition to detailed comparative analyses, it includes the multi-rater LLM evaluation protocol, resource profiling (token usage, working time, and thinking time), Docker-based reproducibility validation, dataset sources, and ablation analyses of the Debug Agent.

Contents

1 Multi-Rater LLM Evaluation Protocol 3

2 Quantifying Token Usage, Working Time, and Thinking Time 7

3 Docker validation 8
3.1 Docker image identification . . . 8
3.2 Representative workflows re-run in Docker . . . 9
3.3 Execution commands . . . 9
3.4 Evidence: key log excerpts and output verification . . . 9

4 Supplemental Data Sources 11

5 Ablation Study: Debug Agent Evaluation 15

6 Comparative Analysis: Hi-C Data Processing 18

7 Comparative Analysis: ChIP-seq Data Processing 21

8 Comparative Analysis: RNA-seq Data Processing 25

9 Comparative Analysis: WGS/WES Data Processing 29

10 Comparative Analysis: microRNA and Long-read Sequencing 31

11 Comparative Analysis: Specialized RNA Sequencing Techniques 38

12 Comparative Analysis: Epigenomics and Proteomics 42

13 Comparative Analysis: Spatial Transcriptomics 45

14 Comparative Analysis: Single-cell RNA Sequencing 50

15 Comparative Analysis: Nanopore Sequencing Technologies 56

16 Comparative Analysis: Comprehensive Whole Genome Sequencing 61

17 Comparative Analysis: Metagenomic Data Processing 64

18 User Case 65

1 Multi-Rater LLM Evaluation Protocol

We sampled 12 tasks that all four systems (BioMaster, ChatGPT, SingleAgent, AutoBA) successfully completed, and applied five LLMs independently scored each system's outputs on seven dimensions: plan-goal alignment, plan completeness, step executability, script quality, task completion, preprocessing consideration, and error handling (1-10 scale). The prompt used to define each rater are shown in Listing 1. Figures S1–S5 show individual rater results.

We quantified inter-rater reliability using the intraclass correlation coefficient (ICC) to measure the degree of agreement among the LLM evaluators. For each scoring dimension, ICC was computed over the full set of 48 item-level scores, treating the LLMs as raters and the workflow outputs as items. We adopted a two-way mixed-effects ICC model with consistency agreement (ICC(3,·)) because the set of evaluators was fixed and we were interested in the consistency of their relative ratings rather than absolute agreement with an external ground truth. ICC was calculated separately for each dimension; results are reported for both single-rater reliability (ICC(3,1)) and evaluator-averaged reliability (ICC(3,5)), the latter corresponding to our aggregation of scores across five evaluators. All results are shown in Table S1.

Table S1: **Inter-rater reliability for the LLM-as-judge raters.** Both single-evaluator reliability (ICC(3,1)) and evaluator-averaged reliability (ICC(3,k), $k=5$) are included.

Dimension	n_{items}	k_{raters}	ICC(3,1)	ICC(3,5)
Plan–Goal Alignment	48	5	0.336	0.717
Plan Completeness	48	5	0.466	0.813
Step Executability	48	5	0.493	0.830
Script Quality	48	5	0.320	0.702
Task Completion	48	5	0.660	0.907
Preprocessing Consideration	48	5	0.709	0.924
Error Handling	48	5	0.316	0.698

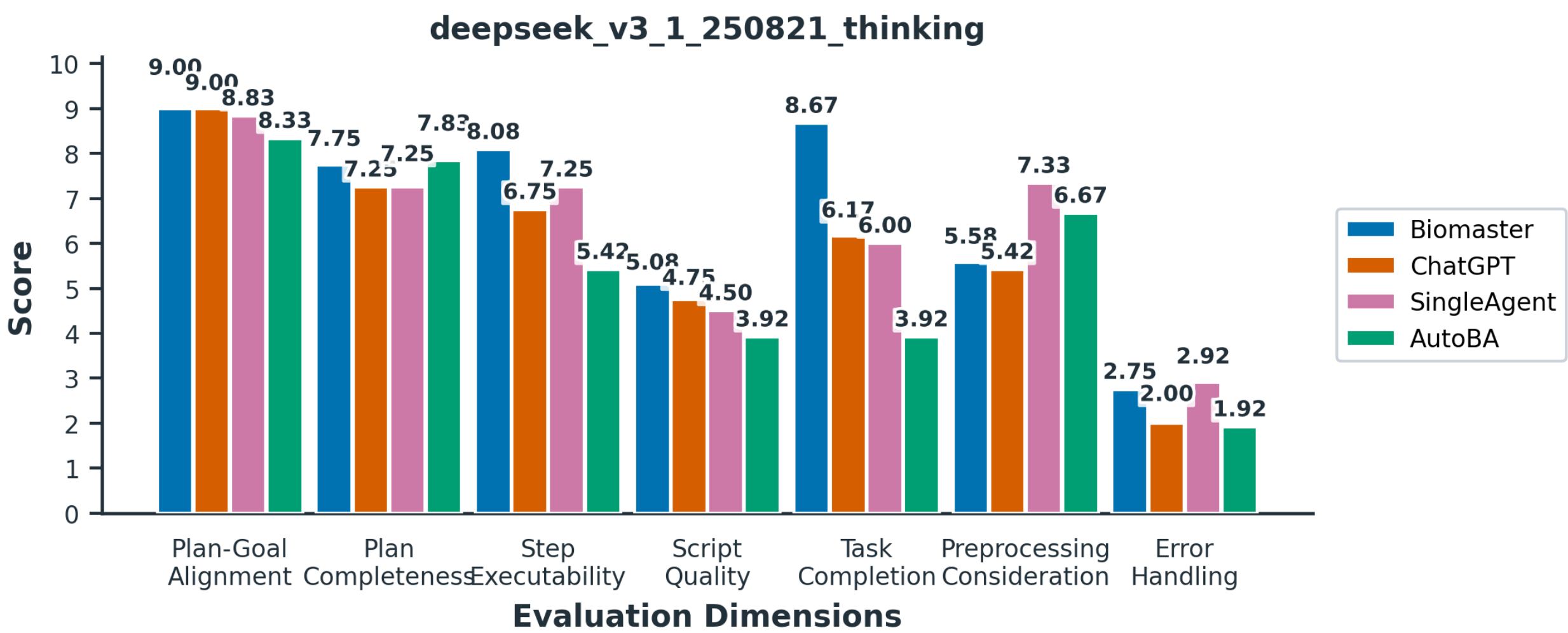

Figure S1: **Per-rater results using DeepSeek-V3 (thinking) as the evaluator.** Scores are averaged over the same 12 randomly sampled, jointly executable tasks and the same seven dimensions as in Fig. 6 a, but reflect ratings from this rater only (no averaging across LLM raters).

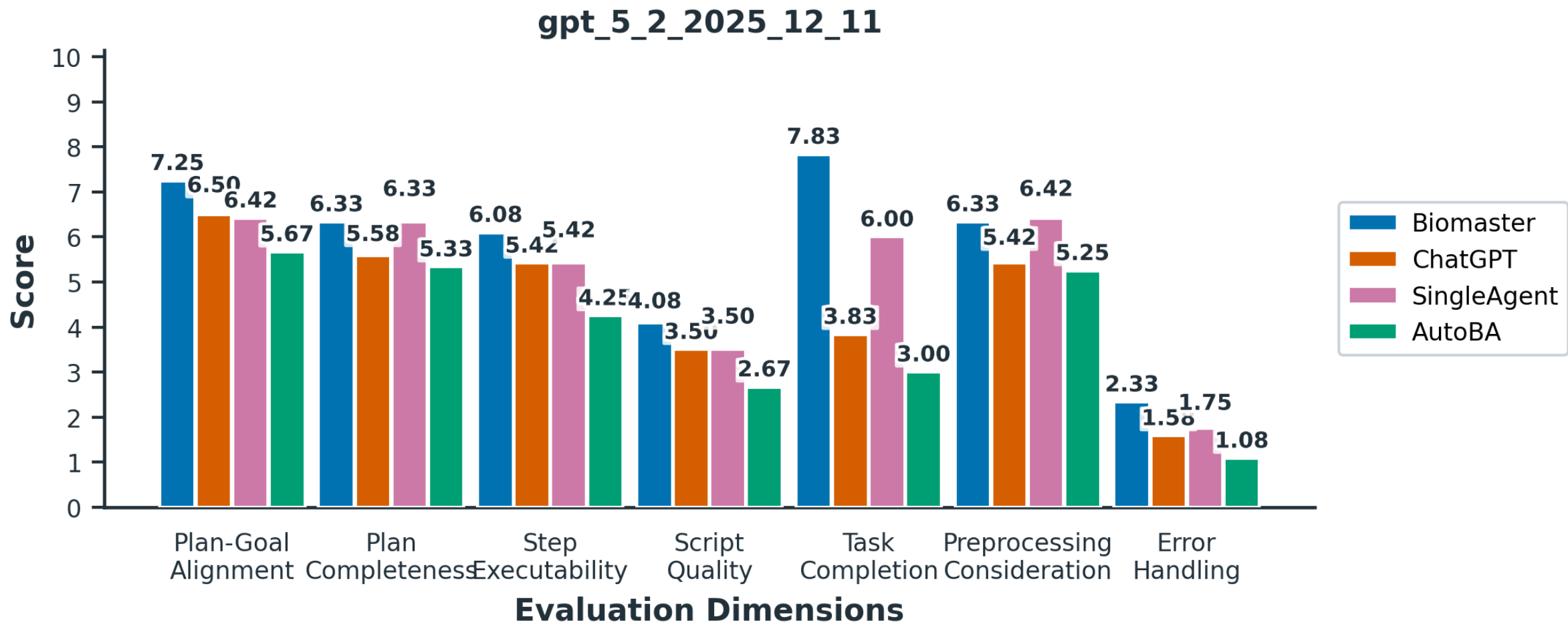

Figure S2: **Per-rater results using GPT-5.2 as the evaluator.** Same task set and seven-dimensional rubric as Fig. 6 a; scores are averaged over tasks for this rater only.

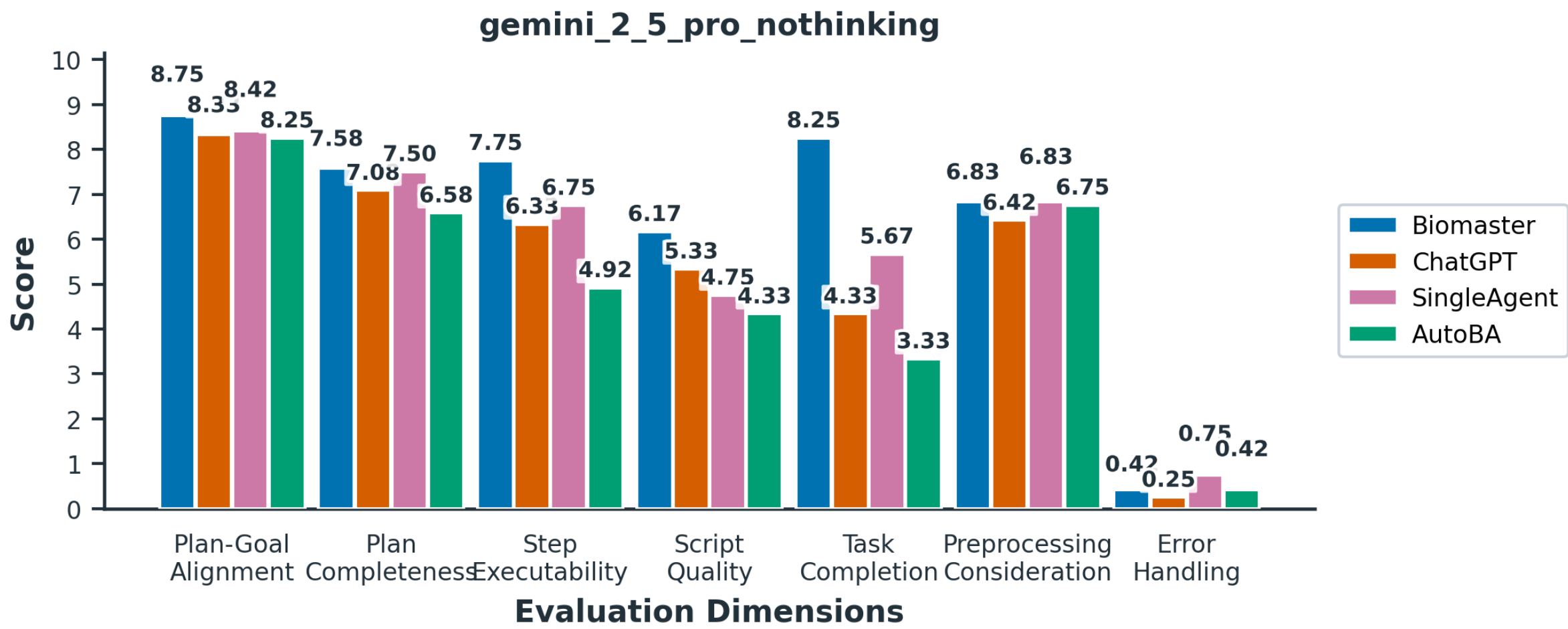

Figure S3: **Per-rater results using Gemini 2.5 Pro (non-thinking) as the evaluator.** Same task set and seven-dimensional rubric as Fig. 6 a; scores are averaged over tasks for this rater only.

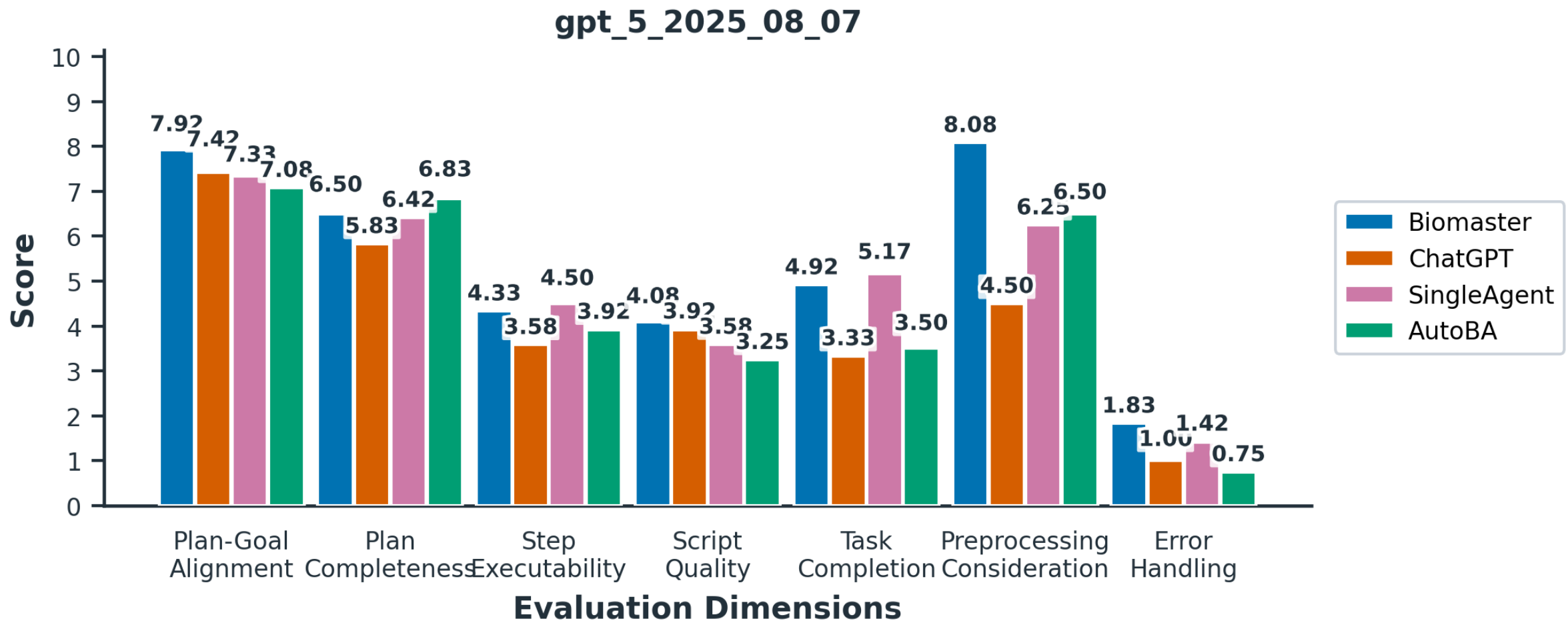

Figure S4: **Per-rater results using GPT-5 as the evaluator.** Same task set and seven-dimensional rubric as Fig. 6 a; scores are averaged over tasks for this rater only.

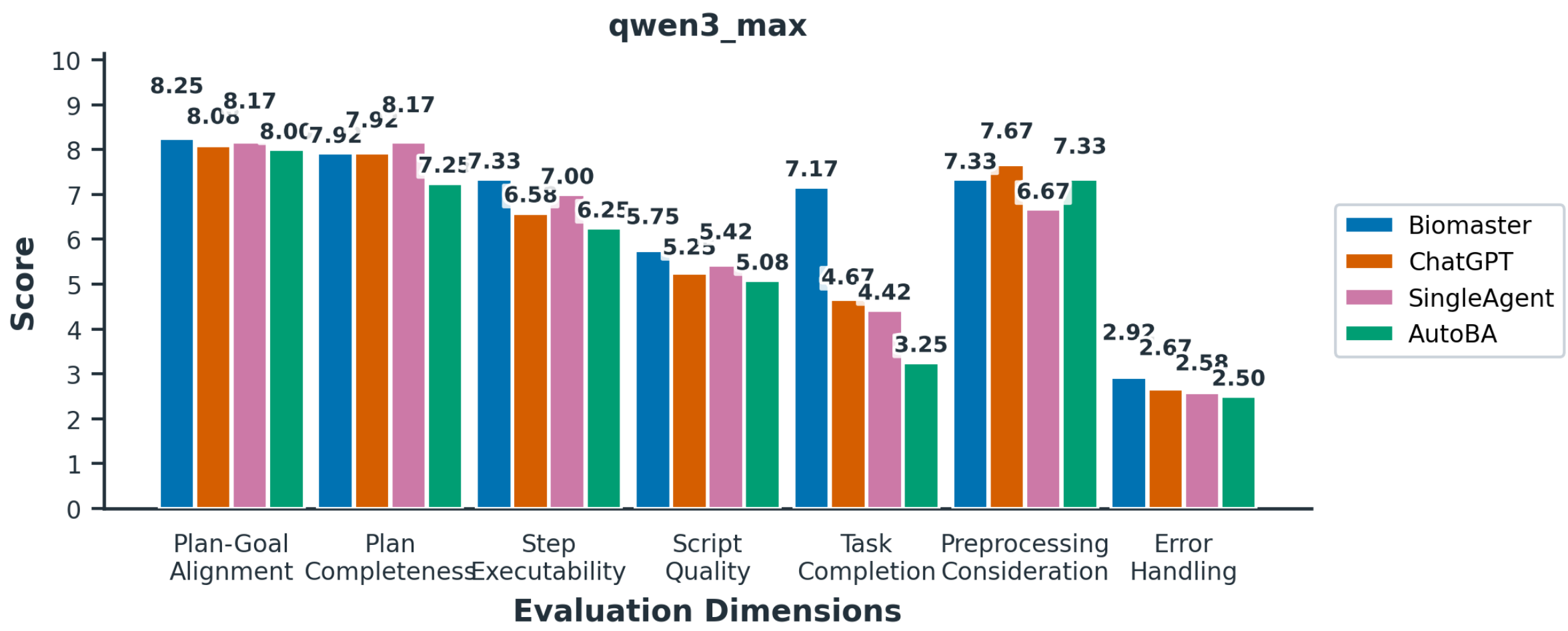

Figure S5: **Per-rater results using Qwen3-Max as the evaluator.** Same task set and seven-dimensional rubric as Fig. 6 a; scores are averaged over tasks for this rater only.

```
You are a bioinformatics analysis evaluator. Your role is to objectively assess the quality of automated
     bioinformatics workflows based on concrete, observable evidence.

## EVALUATION FRAMEWORK

You will evaluate 7 dimensions, each scored 0-10. For each dimension, apply the specific scoring criteria
     below. Base your scores ONLY on evidence present in the provided data.

### DIMENSION 1: Plan-Goal Alignment (0-10)
How well does the analysis plan address the stated biological goal?

SCORING CRITERIA:
- 9-10: Plan directly addresses the goal with scientifically appropriate methods; clear logical flow from goal
      to analysis steps
- 7-8: Plan addresses the goal with suitable methods; minor gaps in logic or method selection
- 5-6: Plan partially addresses the goal; some methods appropriate but others questionable or missing key
     connections
- 3-4: Plan weakly connected to goal; several inappropriate method choices or logical gaps
- 1-2: Plan barely relates to the stated goal; most methods inappropriate
- 0: No plan provided or completely unrelated to goal

### DIMENSION 2: Plan Completeness (0-10)
Does the workflow include all necessary analysis steps?

SCORING CRITERIA:
- 9-10: Includes data QC, preprocessing, main analysis, validation, and output generation; proper step
     sequencing
- 7-8: Includes most essential steps; 1-2 minor steps missing (e.g., some validation)
- 5-6: Core analysis present but missing important preprocessing or validation steps
- 3-4: Several essential steps missing; incomplete workflow
- 1-2: Only 1-2 steps defined; most workflow missing
- 0: No workflow structure defined

### DIMENSION 3: Step Executability (0-10)
Can the defined steps actually be executed?

SCORING CRITERIA:
- 9-10: All steps have correct tool calls, parameters, input/output specifications; dependencies handled
- 7-8: Most steps executable; minor parameter or path issues in 1-2 steps
- 5-6: Some steps executable; others have missing inputs, wrong parameters, or dependency issues
- 3-4: Many steps have execution problems; significant configuration errors
- 1-2: Most steps cannot execute due to missing dependencies, wrong syntax, or invalid parameters
- 0: No executable steps defined

### DIMENSION 4: Script Quality (0-10)
What is the quality of generated code/scripts?

SCORING CRITERIA:
- 9-10: Clean, well-organized code; proper variable naming; comments present; follows bioinformatics
     conventions
- 7-8: Readable code; mostly follows conventions; adequate but not comprehensive documentation
- 5-6: Functional code with readability issues; minimal comments; some convention violations
- 3-4: Poorly organized code; hard to follow; missing documentation; many style issues
- 1-2: Very poor code quality; barely functional; no documentation
- 0: No code/scripts provided

### DIMENSION 5: Task Completion (0-10)
How much of the planned workflow was successfully executed?

SCORING CRITERIA:
- 9-10: All planned steps completed; expected outputs generated; results align with goal
- 7-8: Most steps completed (>75%); main outputs generated; minor issues in some steps
- 5-6: Partial completion (50-75%); some outputs generated; workflow partially achieved goal
- 3-4: Limited completion (25-50%); few outputs; significant execution failures
- 1-2: Minimal completion (<25%); almost no useful outputs
- 0: No steps completed or immediate failure

```

```
### DIMENSION 6: Preprocessing Consideration (0-10)
Is data quality and preparation adequately addressed?

SCORING CRITERIA:
- 9-10: Comprehensive QC (FastQC or equivalent); appropriate filtering/trimming; normalization when needed
- 7-8: QC step present; most preprocessing appropriate; minor gaps
- 5-6: Basic preprocessing mentioned but incomplete; some QC but not comprehensive
- 3-4: Minimal preprocessing; missing critical QC steps; inappropriate methods
- 1-2: Almost no preprocessing; data quality not considered
- 0: No preprocessing when clearly required by data type

### DIMENSION 7: Error Handling (0-10)
How does the system handle errors and failures?

SCORING CRITERIA:
- 9-10: Explicit error checking; logging present; evidence of successful error recovery or debugging
- 7-8: Some error handling; debugging attempts visible; partial recovery from issues
- 5-6: Basic error handling; some log information; limited debugging evidence
- 3-4: Minimal error handling; errors cause workflow to stop; little debugging
- 1-2: Almost no error handling; failures not addressed
- 0: No error handling mechanisms; no evidence of debugging

## EVALUATION PRINCIPLES

1. EVIDENCE-BASED: Score only based on what is explicitly shown in the data. Do not assume or infer
     capabilities not demonstrated.

2. INDEPENDENT DIMENSIONS: Evaluate each dimension separately. A poor score in one dimension should not
     automatically lower scores in others.

3. CALIBRATED SCORING: Use the full 0-10 scale. Avoid clustering scores around any particular range.

4. OBJECTIVE ASSESSMENT: Apply the same criteria consistently. Avoid bias from task complexity or agent type.

OUTPUT FORMAT: Return exactly 7 integers (0-10) separated by single spaces.
Example: 7 6 5 4 6 5 3
```

Listing S1: Evaluator prompt for bioinformatics tasks. The rubric defines seven scoring dimensions (plan–goal alignment, plan completeness, step executability, script quality, task completion, preprocessing consideration, error handling) and enforces an evidence-based 0–10 scoring scheme without default-high anchors.

2 Quantifying Token Usage, Working Time, and Thinking Time

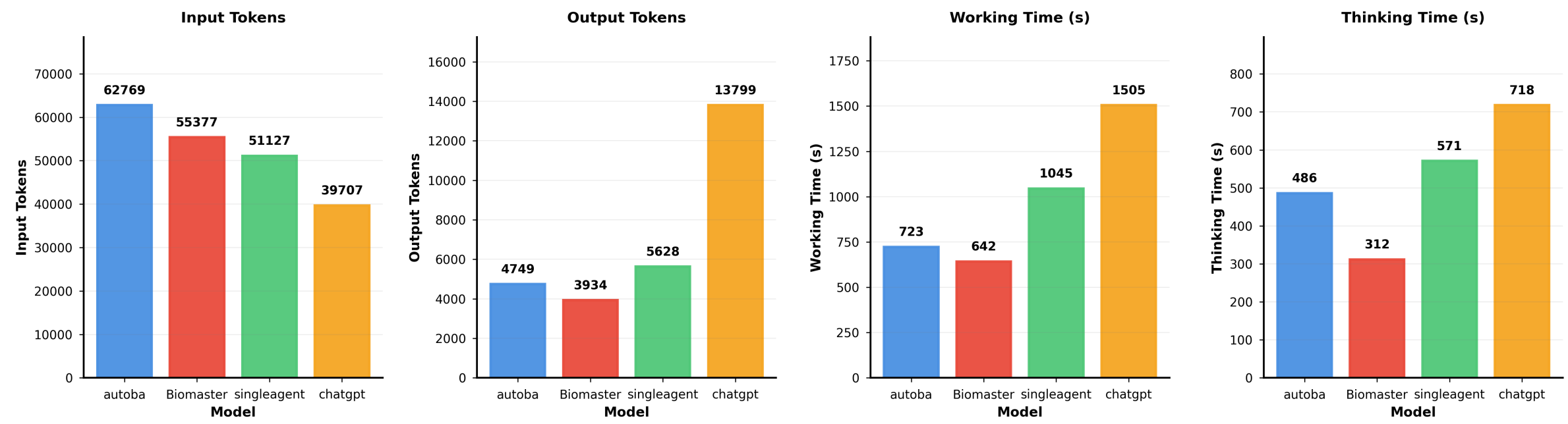

Figure S6: Resource overhead across agents on five representative tasks. Bar charts report, from left to right, Input Tokens, Output Tokens, Working Time (wall-clock seconds for code execution), and Thinking Time (seconds spent awaiting LLM API responses) for AutoBA, BioMaster, SingleAgent, and ChatGPT. Metrics are aggregated over successful runs of five tasks common to all systems: single-cell marker gene identification, single-cell clustering, RNA-seq differential expression, ChIP-seq peak calling, and motif discovery (five runs per task per system). Under identical tool-level knowledge, BioMaster shows lower token usage and shorter working/thinking times than AutoBA and SingleAgent, while ChatGPT exhibits the largest Output Tokens, Working Time, and Thinking Time. Note that these measurements are restricted to tasks completed by all systems; on more complex workflows where baselines fail, their token/time usage can increase due to additional retries and is not reflected here.

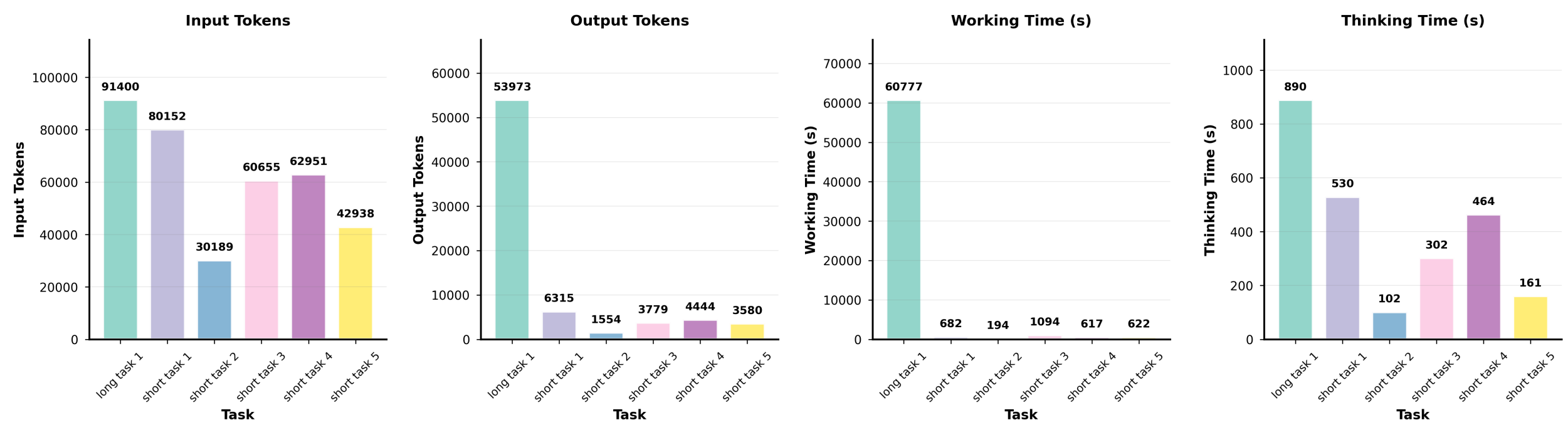

Figure S7: **Computational overhead profiling on representative bioinformatics workflows.** We report four metrics: Input Tokens and Output Tokens (LLM communication cost), Thinking Time (time spent on model reasoning and API responses), and Working Time (wall-clock time consumed by actual bioinformatics tool execution). Five short routine tasks (short tasks 1–5) and one full real-world workflow (long task 1; Hi-C data analysis with multi-stage preprocessing and feature extraction) were evaluated. The results show that, in realistic scenarios, Working Time overwhelmingly dominates total runtime (e.g., 60,777s vs. 890s Thinking Time), confirming that the computational burden primarily arises from underlying bioinformatics programs rather than LLM orchestration. In contrast, short synthetic tasks exhibit a higher proportional contribution from Thinking Time, since their execution workloads are small. This demonstrates that, for real biological data, the practical bottleneck is computation on the data itself, not token usage or LLM latency.

3 Docker validation

3.1 Docker image identification

We validated BioMaster inside a Docker container built from the repository docker/Dockerfile. The image was built and tagged locally as follows.

Image name: biomaster-docker:latest

Image identifier (sha256):

sha256:fbea0767e7662415273e51deddc42bc1ff46ab1cd9d869704530b089bf3b5619

Build command:

```
docker build -f docker/Dockerfile -t biomaster-docker:latest .
```

Verification command:

```
docker image inspect biomaster-docker:latest --format '{{.Id}}'
```

3.2 Representative workflows re-run in Docker

To address reproducibility under containerization, we re-ran two representative single-cell RNA-seq workflows end-to-end in the Docker container: (i) scRNA-seq clustering (5 steps) and (ii) scRNA-seq marker gene identification (6 steps). For each run, we report the exact execution command, key log excerpts indicating step completion, and the resulting output files.

3.3 Execution commands

All commands were executed from the repository root directory (denoted as `$PROJECT_DIR`).

Workflow 1 (scRNA-seq clustering; workflow ID: `gptsccluster`)

```
cd $PROJECT_DIR
./docker/docker-run.sh gptsccluster.yaml
```

Workflow 2 (marker gene identification; workflow ID: `gptscnew1`)

```
cd $PROJECT_DIR
./docker/docker-run.sh gptscnew1.yaml
```

3.4 Evidence: key log excerpts and output verification

For transparency, we provide trimmed excerpts from the execution logs showing successful completion and the key outputs produced at each step. Host-specific absolute paths and external endpoint strings are omitted in the excerpts below.

Workflow 1 (gptsccluster): key log excerpts (trimmed).

```
Step 1: completed; output: ./output/gptsccluster/adata_raw.h5ad
Step 2: completed; output: ./output/gptsccluster/adata_qc.h5ad
Step 3: completed; output: ./output/gptsccluster/adata_norm.h5ad
Step 4: completed; output: ./output/gptsccluster/adata_dimred.h5ad
Step 5: completed; outputs: ./output/gptsccluster/adata_clustered.h5ad
./output/gptsccluster/cluster_plots.png
BioMaster execution completed.
```

Workflow 1 output files (relative paths):

```
./output/gptsccluster/adata_raw.h5ad
./output/gptsccluster/adata_qc.h5ad
./output/gptsccluster/adata_norm.h5ad
./output/gptsccluster/adata_dimred.h5ad
./output/gptsccluster/adata_clustered.h5ad
./output/gptsccluster/cluster_plots.png
```

Workflow 2 (gptscnew1): key log excerpts (trimmed).

```
Step 1: completed; output: ./output/gptscnew1/filtered_singlecellexpress.h5ad
Step 2: completed; output: ./output/gptscnew1/normalized_singlecellexpress.h5ad
Step 3: completed; output: ./output/gptscnew1/pca_singlecellexpress.h5ad
Step 4: completed; output: ./output/gptscnew1/clustered_singlecellexpress.h5ad
```

```
Step 5: completed; output: ./output/gptscnew1/marker_genes.txt
Step 6: completed; outputs: ./output/gptscnew1/umap_clusters.png
./output/gptscnew1/marker_expression_plots.png
BioMaster execution completed.
```

Workflow 2 output files (relative paths):

```
./output/gptscnew1/filtered_singlecellexpress.h5ad
./output/gptscnew1/normalized_singlecellexpress.h5ad
./output/gptscnew1/pca_singlecellexpress.h5ad
./output/gptscnew1/clustered_singlecellexpress.h5ad
./output/gptscnew1/marker_genes.txt
./output/gptscnew1/umap_clusters.png
./output/gptscnew1/marker_expression_plots.png
```

4 Supplemental Data Sources

Overview

Table S2 lists all publicly available datasets used for BioMaster evaluation across 18 biological data types. Datasets were obtained from ENCODE, NCBI SRA, ENA, and other established repositories.

Table S2: **Data sources applied in BioMaster evaluation.** Complete list of publicly available datasets used for benchmarking BioMaster performance across diverse bioinformatics workflows.

Analysis Type	Data Source Link(s)
CAGE-seq Analysis	1. https://www.encodeproject.org/files/ENCFF465ZRB/<br>2. https://ftp.ebi.ac.uk/pub/databases/gencode/Gencode_human/release_47/gencode.v47.primary_assembly.basic.annotation.gtf.gz
DNase-seq Analysis	1. https://www.encodeproject.org/files/ENCFF038HKP/<br>2. https://www.encodeproject.org/files/ENCFF593IOT/
Protein Expression Quantitative Analysis	https://www.ebi.ac.uk/pride/archive/projects/PXD051229
CLIP-seq Analysis	1. https://www.encodeproject.org/files/ENCFF361AWK/<br>2. https://github.com/timflutre/trimmomatic/blob/master/adapters/TruSeq3-PE-2.fa<br>3. https://github.com/carlsonkPhD/tRNA_Charge-Seq/blob/CodovaElife2022/hg38-mature-tRNAs.fa<br>4. https://www.arb-silva.de/no_cache/download/archive/release_138_1/Exports/<br>5. http://ftp.ensembl.org/pub/release-104/fasta/homo_sapiens/dna/Homo_sapiens.GRCh38.dna.primary_assembly.fa.gz<br>6. https://ftp.ensembl.org/pub/release-104/gtf/homo_sapiens/
Ribo-seq Analysis	1. https://www.ncbi.nlm.nih.gov/sra/?term=SRR2007098<br>2. https://github.com/timflutre/trimmomatic/blob/master/adapters/TruSeq3-SE.fa<br>3. http://ftp.ensembl.org/pub/release-104/fasta/homo_sapiens/dna/Homo_sapiens.GRCh38.dna.primary_assembly.fa.gz<br>4. https://ftp.ensembl.org/pub/release-104/gtf/homo_sapiens/
RIP-seq Analysis	1. https://www.ncbi.nlm.nih.gov/sra/?term=SRR15295695<br>2. https://github.com/timflutre/trimmomatic/blob/master/adapters/TruSeq3-SE.fa<br>3. http://ftp.ensembl.org/pub/release-104/fasta/homo_sapiens/dna/Homo_sapiens.GRCh38.dna.primary_assembly.fa.gz<br>4. https://ftp.ensembl.org/pub/release-104/gtf/homo_sapiens/
Spatial Transcriptomics Analysis	1. https://cf.10xgenomics.com/samples/spatial-exp/1.3.0/Visium_FFPE_Mouse_Brain/Visium_FFPE_Mouse_Brain_fastqs.tar

Table S2: **Data sources applied in BioMaster evaluation.** Complete list of publicly available datasets used for benchmarking BioMaster performance across diverse bioinformatics workflows.

**Analysis Type**	**Data Source Link(s)**
	2. https://cf.10xgenomics.com/samples/spatial-exp/1.3.0/Visium_FFPE_Mouse_Brain/Visium_FFPE_Mouse_Brain_image.jpg
WGS/WES Analysis	1. https://www.ncbi.nlm.nih.gov/sra/ERR14089508<br>2. https://www.ncbi.nlm.nih.gov/sra/SRX26690893<br>3. https://ftp.sra.ebi.ac.uk/vol1/fastq/SRR318/003/SRR3182423/SRR3182423_1.fastq.gz<br>4. https://ftp.sra.ebi.ac.uk/vol1/fastq/SRR318/003/SRR3182423/SRR3182423_2.fastq.gz
microRNA Analysis	https://nf-co.re/smrnaseq/2.4.0/
3' End-seq Analysis	https://nf-co.re/smrnaseq/2.4.0/
PacBio RNA-seq Analysis	https://nf-co.re/smrnaseq/2.4.0/
Nanopore Analysis	https://www.ebi.ac.uk/ena/browser/home
Bisulfite-Seq Analysis	1. gs://nigms-sandbox/dna-methyl/Habibi2013_chr6F3A.tar.gz<br>2. gs://nigms-sandbox/dna-methyl/Mus_musculus.GRCm39.dna.chromosome.6.fa
ChIP-seq Analysis	1. https://www.ebi.ac.uk/<br>2. ftp://ftp.sra.ebi.ac.uk/vol1/fastq/SRR620/SRR6204/SRR6204.fastq.gz<br>3. ftp://ftp.sra.ebi.ac.uk/vol1/fastq/SRR620/SRR6204/SRR6205.fastq.gz<br>4. ftp://ftp.sra.ebi.ac.uk/vol1/fastq/SRR620/SRR6204/SRR6206.fastq.gz<br>5. ftp://ftp.sra.ebi.ac.uk/vol1/fastq/SRR620/SRR6204/SRR6207.fastq.gz<br>6. ftp://ftp.sra.ebi.ac.uk/vol1/fastq/SRR620/SRR6204/SRR6208.fastq.gz<br>7. http://hgdownload.cse.ucsc.edu/goldenPath/mm10/bigZips/chromFa.tar.gz
RNA-seq Analysis	1. https://github.com/STAR-Fusion/STAR-Fusion-Tutorial.git<br>2. http://hgdownload.soe.ucsc.edu/goldenPath/mm39/bigZips/mm39.fa.gz

Table S2: **Data sources applied in BioMaster evaluation.** Complete list of publicly available datasets used for benchmarking BioMaster performance across diverse bioinformatics workflows.

Analysis Type	Data Source Link(s)
	3. ftp://ftp.sra.ebi.ac.uk/vol1/fastq/SRR137/001/SRR1374921/SRR1374921.fastq.gz<br>4. ftp://ftp.sra.ebi.ac.uk/vol1/fastq/SRR137/002/SRR1374922/SRR1374922.fastq.gz<br>5. ftp://ftp.sra.ebi.ac.uk/vol1/fastq/SRR137/003/SRR1374923/SRR1374923.fastq.gz<br>6. ftp://ftp.sra.ebi.ac.uk/vol1/fastq/SRR137/004/SRR1374924/SRR1374924.fastq.gz<br>7. http://hgdownload.soe.ucsc.edu/goldenPath/mm39/bigZips/genes/mm39.ncbiRefSeq.gtf.gz<br>8. https://www.ncbi.nlm.nih.gov/sra/?term=SRR1039500<br>9. https://github.com/timflutre/trimmomatic/blob/master/adapters/TruSeq3-SE.fa<br>10. http://ftp.ensembl.org/pub/release-104/fasta/homo_sapiens/dna/Homo_sapiens.GRCh38.dna.primary_assembly.fa.gz<br>11. https://ftp.ensembl.org/pub/release-104/gtf/homo_sapiens/
Hi-C Analysis	https://data.4dnucleome.org/resources/data-analysis/hi_c-processing-pipeline
scRNA-seq Analysis	https://figshare.com/articles/dataset/Kang_HM_Subramaniam_M_Targ_S_Nguyen_M_et_al_2017/19397624
Metagenomic Analysis	https://github.com/taowenmicro/EasyMicrobiomeR

5 Ablation Study: Debug Agent Evaluation

Overview

We performed ablation studies on 5 bioinformatics pipelines to assess the Debug Agent's contribution, running each pipeline 5 times with and without the Debug Agent. The Debug Agent substantially improves execution success, with particularly pronounced gains in ChIP-seq peak calling, WGS/WES somatic analysis, Hi-C processing, and RNA-seq differential expression.

Table S3: **Ablation study on Debug Agent component.** Comparison of BioMaster performance with and without the Debug Agent across 5 bioinformatics pipelines and 5 independent runs each. Success indicates successful execution; fail indicates failed execution; dash (-) indicates step not reached due to upstream failure.

Pipeline	Steps	Case 1	Case 2	Case 3	Case 4	Case 5	Total Success Rate
CHIP-Seq call peaks BioMaster	quality control and adapter trimming	success	success	success	success	success	1
	Align	success	success	success	success	success	1
	SAM files to sorted BAM	success	success	success	success	success	1
	Remove PCR duplicates	success	success	success	success	success	1
	call peak	success	success	success	success	success	1
CHIP-Seq call peaks BioMaster w/o Debug	quality control and adapter trimming	success	success	success	success	success	1
	Align	fail	fail	fail	success	success	0.4
	SAM files to sorted BAM	-	-	-	fail	fail	0
	Remove PCR duplicates	-	-	-	-	-	0
	call peak	-	-	-	-	-	0
WGS/WES analysis Somatic BioMaster	quality control and adapter trimming	success	success	success	success	success	1
	Align	success	success	success	success	success	1
	SAM files to sorted BAM	success	success	success	success	success	1
	Remove PCR duplicates	success	fail	success	success	success	0.8
	somatic SNV and indel calling	success	-	success	success	success	0.8
WGS/WES analysis Somatic BioMaster w/o Debug	quality control and adapter trimming	fail	success	success	success	success	0.8
	Align	-	success	success	fail	fail	0.4
	SAM files to sorted BAM	-	success	success	-	-	0.4
	Remove PCR duplicates	-	fail	fail	-	-	0
	somatic SNV and indel calling	-	-	-	-	-	0
HIC Data Analysis BioMaster	align	fail	success	success	success	success	0.8
	Parse and sort the unsorted BAM files	-	success	success	success	success	0.8
	Merge the two pairsam files	-	success	fail	success	success	0.6
	Mark duplicate reads in the merged pairsam file	-	success	-	success	success	0.6
	Filter and annotate the duplicate-marked pairsam file	-	success	-	success	success	0.6
	Merge the final pairs files	-	success	-	success	success	0.6

Continued on next page

Table S3 – continued from previous page

Pipeline	Steps	Case 1	Case 2	Case 3	Case 4	Case 5	Total Success Rate
	Create an unnormalized contact matrix	-	success	-	success	success	0.6
	Balance (normalize) the contact matrix	-	success	-	success	success	0.6
	Generate a multi-resolution	-	success	-	success	success	0.6
HIC Data Analysis BioMaster w/o Debug	align	fail	fail	fail	success	success	0.4
	Parse and sort the unsorted BAM files	-	-	-	fail	success	0.2
	Merge the two pairsam files	-	-	-	-	fail	0
	Mark duplicate reads in the merged pairsam file	-	-	-	-	-	0
	Filter and annotate the duplicate-marked pairsam file	-	-	-	-	-	0
	Merge the final pairs files	-	-	-	-	-	0
	Create an unnormalized contact matrix	-	-	-	-	-	0
	Balance (normalize) the contact matrix	-	-	-	-	-	0
	Generate a multi-resolution	-	-	-	-	-	0
RNA Identify top5 down regulated genes BioMaster	quality control and adapter trimming	success	success	success	success	success	1
	Align	success	success	success	success	success	1
	SAM files to sorted BAM	success	success	success	success	success	1
	Remove PCR duplicates	success	success	success	success	success	1
	Quantify gene expression	success	success	success	success	success	1
	Conduct differential expression analysis	success	success	success	success	success	1
	Filter and rank the differential expression result	success	success	success	success	success	1
RNA Identify top5 down regulated genes BioMaster w/o Debug	quality control and adapter trimming	fail	success	success	success	success	0.8
	Align	-	fail	fail	fail	success	0.2
	SAM files to sorted BAM	-	-	-	-	success	0.2
	Remove PCR duplicates	-	-	-	-	success	0.2
	Quantify gene expression	-	-	-	-	fail	0
	Conduct differential expression analysis	-	-	-	-	-	0
	Filter and rank the differential expression result	-	-	-	-	-	0

6 Comparative Analysis: Hi-C Data Processing

Overview

Tables S4–S8 compare system performance on Hi-C data analysis, covering alignment, pair processing, filtering, contact matrix generation, and normalization. BioMaster completes all 9 workflow steps, while ChatGPT and AutoBA fail at step 5 (filter and annotate) due to incorrect pairtools usage.

Table S4: **Hi-C data analysis comparison (Part 1).** Comparison of alignment and initial processing steps across three systems. ✓ indicates successful step completion; × indicates failure.

Hi-C analysis	Alignment	Parse & sort BAM	Merge pairsam files	Mark duplicates
Tools	bwa	samtools, pairtools	pairtools	pairtools, pairix
ChatGPT	✓	✓	✓	✓
AutoBA	✓	✓	✓	✓
BioMaster	✓	✓	✓	✓

Table S5: **Hi-C data analysis comparison (Part 2).** Comparison of advanced processing steps including filtering, matrix generation, and normalization. Only BioMaster successfully completes all downstream analysis steps.

Hi-C analysis	Filter & annotate	Merge pairs files	Create contact matrix	Balance matrix	Generate multi-resolution
Tools	pairtools	pairix	cooler	cooler	cooler
ChatGPT	wrongly use pairtools	×	×	×	×
AutoBA	wrongly use pairtools	×	×	×	×
BioMaster	✓	✓	✓	✓	✓

Table S6: **Hi-C data mapping and sorting.** All three systems successfully perform basic alignment and BAM sorting operations.

**Hi-C data mapping, sorting and conversion**	**Alignment**	**BAM sorting**
Tools	bwa	samtools
ChatGPT	✓	✓
AutoBA	✓	✓
BioMaster	✓	✓

Table S7: **Hi-C contact matrix processing.** ChatGPT fails at matrix creation due to incorrect cooler settings; AutoBA and BioMaster succeed at all steps.

**Hi-C data contact matrix processing**	**Create unnormalized matrix**	**Balance matrix**	**Generate multi-resolution cooler files**
Tools	cooler	cooler	cooler
ChatGPT	wrongly use cooler setting	×	×
AutoBA	✓	✓	✓
BioMaster	✓	✓	✓

Table S8: **Hi-C pair parsing and cleaning.** BioMaster demonstrates superior handling of pairtools and pairix workflows; ChatGPT and AutoBA fail at filtering and merging steps.

**Hi-C data pair parsing and cleaning**	**Merge pairsam files**	**Mark duplicates**	**Filter & annotate**	**Merge pairs files**
Tools	pairtools	pairtools, pairix	pairtools	pairix
ChatGPT	✓	wrongly use pairtools	×	×
AutoBA	✓	✓	wrongly use pairtools	×
BioMaster	✓	✓	✓	✓

7 Comparative Analysis: ChIP-seq Data Processing

Overview

Tables S9–S13 evaluate ChIP-seq workflows including peak calling, functional enrichment, and motif discovery. All systems complete basic peak calling and motif discovery, but only BioMaster successfully performs functional enrichment analysis with peak annotation, differential binding, and pathway enrichment.

Table S9: **ChIP-seq peak calling workflow.** All three systems successfully complete the standard ChIP-seq peak calling pipeline.

**ChIP-seq peak calling**	**Quality control & trimming**	**Alignment**	**Sort & remove duplicates**	**Peak calling**
Tools	FastQC, Trimmomatic	BWA-MEM	SAMtools, Picard	MACS3
ChatGPT	✓	✓	✓	✓
AutoBA	✓	✓	✓	✓
BioMaster	✓	✓	✓	✓

Table S10: **ChIP-seq functional enrichment analysis (Part 1).** Initial processing steps: ChatGPT encounters errors in SAM conversion; AutoBA fails at signal track generation.

**Functional Enrichment (Part 1)**	**Quality control**	**Alignment**	**SAM to BAM**	**Signal tracks**
Tools	FastQC, Trimmomatic, Cutadapt	BWA	samtools	deeptools
ChatGPT	✓	✓	wrong bwa output file	×
AutoBA	✓	✓	✓	wrong use deeptools
BioMaster	✓	✓	✓	✓

Table S11: **ChIP-seq functional enrichment analysis (Part 2).** Advanced analysis steps: only BioMaster successfully completes peak calling, annotation, differential binding, and enrichment analysis.

**Functional Enrichment (Part 2)**	**Call peaks**	**Annotate peaks**	**Differential binding**	**Enrichment analysis**
Tools	MACS3	HOMER	DESeq2	ClusterProfiler
ChatGPT	×	×	×	×
AutoBA	×	×	×	×
BioMaster	✓	✓	✓	✓

Table S12: **ChIP-seq motif discovery (Part 1).** All systems successfully complete initial processing for motif discovery workflow.

**Motif Discovery**	**Quality control**	**Alignment**	**SAM to BAM**	**Signal tracks**
Tools	FastQC, Trimmomatic, Cutadapt	BWA	samtools	deeptools
ChatGPT	✓	✓	✓	✓
AutoBA	✓	✓	✓	✓
BioMaster	✓	✓	✓	✓

Table S13: **ChIP-seq motif discovery (Part 2).** All systems successfully complete peak calling, annotation, and motif discovery using HOMER.

**Motif Discovery**	**Call peaks**	**Annotate peaks**	**Discover DNA motifs**
Tools	MACS3	HOMER	HOMER
ChatGPT	✓	✓	✓
AutoBA	✓	✓	✓
BioMaster	✓	✓	✓

8 Comparative Analysis: RNA-seq Data Processing

Overview

Tables S14–S21 cover 10 RNA-seq analysis types: alternative polyadenylation (APA), differential expression, isoform quantification, fusion detection, and RNA editing. BioMaster completes 9/10 tasks, compared to ChatGPT (5/10) and AutoBA (4/10). APA analysis remains challenging for all systems.

Table S14: **RNA-seq alternative polyadenylation (APA) analysis (Part 1).** ChatGPT and AutoBA fail at alignment step; BioMaster successfully completes initial processing.

RNA-seq APA (Part 1)	Alignment	SAM to BAM	Index BAM	Read depth profile
Tools	STAR	samtools	samtools	samtools
ChatGPT	×	×	×	×
AutoBA	Keep thinking	×	×	×
BioMaster	✓	✓	✓	✓

Table S15: **RNA-seq APA analysis (Part 2).** Downstream APA quantification steps; BioMaster successfully extracts regions and quantifies APA events.

RNA-seq APA (Part 2)	Extract genomic regions	Quantify APA
Tools	R	R
ChatGPT	×	×
AutoBA	×	×
BioMaster	✓	✓

Table S16: **RNA-seq differential gene expression analysis.** All systems successfully complete the standard differential expression workflow.

Differential Expression	Quality control	Alignment	SAM to BAM	Gene expression quantification	Differential analysis
Tools	Trimmomatic	BWA	samtools	HTSeq	DESeq2
ChatGPT	✓	✓	✓	✓	✓
AutoBA	✓	✓	✓	✓	✓
BioMaster	✓	✓	✓	✓	✓

Table S17: **RNA-seq top downregulated genes identification (Part 1).** All systems successfully complete initial processing steps.

Top Downregulated Genes identification	Quality control	Alignment	SAM to BAM	Sort BAM
Tools	Trimmomatic	BWA	samtools	samtools
ChatGPT	✓	✓	✓	✓
AutoBA	✓	✓	✓	✓
BioMaster	✓	✓	✓	✓

Table S18: **RNA-seq top downregulated genes identification (Part 2).** All systems successfully complete gene quantification, differential analysis, and result filtering.

Top Downregulated Genes identification	Gene expression quantification	Differential expression analysis	Filter and rank results
Tools	HTSeq	DESeq2	R
ChatGPT	✓	✓	✓
AutoBA	✓	✓	✓
BioMaster	✓	✓	✓

Table S19: **RNA-seq isoform expression analysis.** ChatGPT fails at STAR alignment; AutoBA and BioMaster successfully complete isoform quantification.

RNA Isoform Expression	Quality control	Transcriptome alignment	Convert SAM	Isoform quantification
Tools	Trimmomatic	STAR	Samtools	RESM
ChatGPT	✓	failed use STAR	×	×
AutoBA	✓	✓	✓	✓
BioMaster	✓	✓	✓	✓

Table S20: **RNA-seq fusion gene prediction.** All systems successfully complete fusion gene detection using STAR-Fusion.

RNA-seq Fusion Gene Prediction	RNA-seq read alignment	Fusion genes detection
Tools	STAR	STAR-Fusion
ChatGPT	✓	✓
AutoBA	✓	✓
BioMaster	✓	✓

Table S21: **RNA-seq editing analysis.** AutoBA gets stuck at alignment step; ChatGPT and BioMaster successfully identify RNA editing events.

**RNA Editing**	**Quality control**	**Transcriptome alignment**	**SAM to sorted BAM**	**Variant calling**	**Identify editing events**
Tools	Trimmomatic	STAR	Samtools	Samtools	R
ChatGPT	✓	✓	✓	✓	✓
AutoBA	✓	Keep thinking	×	×	×
BioMaster	✓	✓	✓	✓	✓

9 Comparative Analysis: WGS/WES Data Processing

Overview

Tables S22–S24 evaluate WGS/WES workflows including differential expression, somatic variant calling, and variant annotation. All systems complete differential expression and somatic variant calling. ChatGPT fails at variant annotation due to incorrect GATK Funcotator usage.

Table S22: **WGS differential gene expression analysis.** All systems successfully complete transcriptome alignment and differential analysis.

WGS Differential Expression	Transcriptome alignment	Sort & index BAM	Differential expression analysis
Tools	STAR	Samtools	DESeq2
ChatGPT	✓	✓	✓
AutoBA	✓	✓	✓
BioMaster	✓	✓	✓

Table S23: **WGS/WES somatic variant calling.** All systems successfully complete the full somatic variant calling pipeline.

Somatic Variant Calling	Genome alignment	SAM to sorted BAM	Mark duplicates	SNV & indel calling	Filter variants
Tools	STAR	samtools	GATK	GATK	GATK
ChatGPT	✓	✓	✓	✓	✓
AutoBA	✓	✓	✓	✓	✓
BioMaster	✓	✓	✓	✓	✓

Table S24: **WGS/WES somatic variant annotation.** ChatGPT fails at GATK Funcotator usage; AutoBA and BioMaster successfully annotate variants.

Somatic Variant Annotation	RNA-seq alignment	SAM conversion	Sort & index BAM	SNV & indel calling	Variant annotation
Tools	STAR	Samtools	Samtools	GATK	GATK Funcotator
ChatGPT	✓	✓	✓	✓	wrong use GATK Funcotator
AutoBA	✓	✓	✓	✓	✓
BioMaster	✓	✓	✓	✓	✓

10 Comparative Analysis: microRNA and Long-read Sequencing

Overview

Tables S25–S31 evaluate microRNA analysis and long-read sequencing (Nanopore, PacBio). For microRNA quantification, only BioMaster succeeds (ChatGPT and AutoBA fail at bowtie alignment). For miRNA prediction, ChatGPT and BioMaster succeed while AutoBA incorrectly uses miRDeep2. Only BioMaster completes the full PacBio RNA-seq pipeline. Polyadenylation site (PAS) identification remains challenging for all systems.

Table S25: **MicroRNA quantification.** ChatGPT and AutoBA fail at alignment using bowtie; BioMaster successfully quantifies miRNA expression.

**microRNA quantification**	**quality control and trim using FastQC**	**alignment and trim reads to reference sequence**	**Quantify miRNA expression**
Tools	FastQC, fastq	bowtie1, samtools	featureCounts
ChatGPT	✓	failed to align using bowtie2	×
AutoBA	✓	wrongly use bowtie2 to read genome.fa	×
BioMaster	✓	✓	✓

Table S26: **MicroRNA prediction.** AutoBA incorrectly uses miRDeep2; ChatGPT and BioMaster successfully identify novel miRNAs.

**microRNA prediction**	**quality control and trim using FastQC**	**alignment and trim reads to reference genome**	**Quantify miRNA expression levels**	**Identify novel miRNAs using miRDeep2**
Tools	FastQC, fastq	bowtie1, samtools	featureCounts	miRDeep2
ChatGPT	✓	✓	✓	✓
AutoBA	✓	✓	Wrongly Use miRDeep2 to quantify known miRNAs	×
BioMaster	✓	✓	✓	✓

Table S27: **Nanopore RNA-seq analysis (Part 1).** AutoBA fails at minimap2 usage; ChatGPT and BioMaster complete isoform expression analysis.

**Isoform Expression Analysis**	**Align reads to the human reference genome**	**Convert and sort the SAM file to BAM format**	**Use StringTie to reconstruct transcripts and quantify their expression**
Tools	minimap2	Samtools	StringTie
ChatGPT	✓	✓	✓
AutoBA	Wrongly use minimap2 to read file	×	×
BioMaster	✓	✓	✓

Table S28: **BioMaster plan for Nanopore RNA-seq (Part 2).** Detailed workflow showing quality control, alignment, and quantification steps.

**Isoform Expression Analysis**	**Perform internal quality control and adapter trimming**	**Align the trimmed Nanopore reads to reference genome**	**Convert SAM to BAM format**	**Sort and index the BAM file**	**Quantify transcript isoform expression**
Tools	internal_trim_qc	minimap2	samtools	samtools	featureCounts
BioMaster	✓	✓	✓	✓	✓

Table S29: **PacBio RNA-seq analysis.** ChatGPT fails at SAM conversion; AutoBA fails at quality assessment; BioMaster completes full pipeline.

PacBio RNA sequencing data analysis	Perform internal quality control and adapter trimming	Align the trimmed reads to reference genome	Convert SAM to BAM format	Sort and index the BAM file	Quantify transcript expression
Tools	internal_trim_qc	minimap2	samtools	samtools	featureCounts
ChatGPT	✓	✓	failed to use samtool to convert format	×	×
AutoBA	Wrongly use manaplot for quality assessment	×	×	×	×
BioMaster	✓	✓	✓	✓	✓

Table S30: **PAS identification (Part 1).** AutoBA fails at adapter trimming; ChatGPT cannot identify high-confidence PAS.

PAS (polyadenylation site) identification	Quality control & adapter trimming	Remove trailing polyA sequences	Cluster reads by similarity	Build consensus sequences	Identify high-confidence PAS
Tools	Trimmomatic	Cutadapt	VSEARCH	NA	NA
ChatGPT	✓	✓	✓	✓	cannot identify PAS
AutoBA	Wrongly use trim galore	×	×	×	×

Table S31: **PAS identification (Part 2).** BioMaster workflow for PAS clustering; limited success in final PAS identification.

PAS (polyadenylation site) identification	Quality control & adapter trimming	Extract last 20nt from reads	Cluster similar 3′ end sequences
Tools	fastp	extract_3p_end	cluster_pas_peaks
BioMaster	✓	✓	cannot identify PAS

Table S32: **RNA-seq expression quantification.** ChatGPT and AutoBA fail at alignment; BioMaster successfully quantifies expression.

RNA-Seq Expression quantification	Quality control	Trimming	Aligning	Quantifying expression
Tools	FastQC	Trimmomatic	STAR/Hisat2	featureCounts
ChatGPT	✓	✓	failing in using STAR	×
AutoBA	✓	✓	failing in using Hisat2	×
BioMaster	✓	✓	✓	✓

Table S33: **RNA-seq functional annotation and enrichment.** Only BioMaster completes full functional annotation pipeline.

RNA-Seq Functional annotation	Quality control	Trimming	Aligning	Counting reads	Differential expression	Functional annotation & enrichment
Tools	FastQC	Trimmomatic	STAR/HISAT2	featureCounts	DESeq2	clusterProfiler
ChatGPT	✓	✓	failing in using STAR	×	×	×
AutoBA	✓	✓	failing in using Hisat2	×	×	×
BioMaster	✓	✓	✓	✓	✓	✓

Table S34: **Circular RNA identification.** ChatGPT and AutoBA fail at BWA alignment; BioMaster successfully identifies circRNAs.

**Circular RNA identification**	**Quality control**	**Trimming**	**Aligning**	**Indexing**	**Identifying candidate circular RNAs**
Tools	FastQC	Trim Galore	STAR/BWA	samtools	CIRCexplorer2/CIRIquant
ChatGPT	✓	✓	failing using BWA	×	×
AutoBA	✓	✓	failing in using BWA	×	×
BioMaster	✓	✓	✓	✓	✓

11 Comparative Analysis: Specialized RNA Sequencing Techniques

Overview

Tables S32–S38 cover specialized RNA techniques: CLIP-seq, RIP-seq, Ribo-seq, and splicing analysis. BioMaster completes all tasks successfully. ChatGPT and AutoBA encounter various failures including incorrect tool selection (CLIPper, RiboCode, rMATS), alignment errors, and peak annotation issues.

Table S35: **CLIP-seq crosslink identification.** ChatGPT fails at CLIPper usage; AutoBA returns invalid response; BioMaster succeeds.

**Clipseq**	**Adapter and Quality Trimming**	**Pre-mapping to rRNA and tRNA**	**Genome Mapping**	**UMI-Based Deduplication**	**Crosslink Identification**
Tools	Cutadapt	Bowtie2	Bowtie2	UMI-tools	BEDTools
ChatGPT	✓	✓	✓	✓	fail in using CLIPper to recognize cross link sites
AutoBA	✓	✓	✓	✓	invalid response in reading file
BioMaster	✓	✓	✓	✓	✓

Table S36: **RIP-seq RBP-bound gene identification.** ChatGPT and AutoBA fail at peak annotation; BioMaster completes full analysis.

**RIP-seq**	**Adapter and Quality Trimming**	**Genome Mapping**	**Convert SAM to sorted BAM**	**Identify potential RBP binding sites**	**Annotate the detected peaks**	**Extract enriched genes**
Tools	Cutadapt	Bowtie2	Samtools	pureclip	BEDTools	BEDTools
ChatGPT	✓	✓	✓	✓	fail in using MACS2 to recognize called peaks for RBP-bound regions	×
AutoBA	✓	✓	✓	✓	fail in using DESeq2 in R	×
BioMaster	✓	✓	✓	✓	✓	✓

Table S37: **Ribo-seq ORF detection.** ChatGPT incorrectly uses RiboCode; AutoBA fails at alignment; BioMaster succeeds.

**Ribo-seq**	**Adapter and Quality Trimming**	**Genome Mapping**	**Convert SAM to sorted BAM**	**Identify actively translated ORFs**
Tools	Trimmomatic	Bowtie2	Samtools	ribotricer
ChatGPT	✓	✓	✓	Wrongly use RiboCode to identify translated ORFs
AutoBA	✓	fail in using bowtie2	×	×
BioMaster	✓	✓	✓	✓

Table S38: **RNA-seq splicing analysis.** ChatGPT and AutoBA fail at rMATS usage; BioMaster successfully identifies alternative splicing.

**RNA-seq Splicing analysis**	**Adapter and Quality Trimming**	**Genome Mapping**	**Run rMATS to identify alternative splicing events**
Tools	Trimmomatic	Bowtie2	rMATS
ChatGPT	✓	✓	fail in use rMATS to recognize alternative splicing
AutoBA	✓	✓	×, no output file
BioMaster	✓	✓	✓

12 Comparative Analysis: Epigenomics and Proteomics

Overview

Tables S39–S42 evaluate epigenomics (WGS/WES structural variants, DNase-seq, CAGE-seq) and proteomics (mass spectrometry). BioMaster completes all analyses. ChatGPT and AutoBA fail across all tasks due to incorrect tool usage (Delly, samtools, CAGEr, limma).

Table S39: **WGS/WES structural variation.** ChatGPT and AutoBA encounter tool usage errors; BioMaster successfully detects SVs.

**WGS/WES Structure variation identification**	**Trim low-quality bases**	**Alignment**	**Parse & sort**	**Structural variant detection**
Tools	Trimmomatic	BWA	samtools	Delly/cuteSV
ChatGPT	✓	✓	✓	Wrongly use Delly
AutoBA	✓	Wrongly use BWA	×	×
BioMaster	✓	✓	✓	✓

Table S40: **DNase-seq analysis.** ChatGPT fails at samtools usage; AutoBA fails at trimming; BioMaster completes analysis.

DNase-seq	Quality control and adapter trimming	Alignment	Parse & sort	Index the sorted BAM file	Identify
Tools	Trimmomatic	BWA	samtools	samtools	MACS2
ChatGPT	✓	✓	wrongly use samtools	×	×
AutoBA	Wrongly use trimmomatic	×	×	×	×
BioMaster	✓	✓	✓	✓	✓

Table S41: **CAGE-seq analysis.** ChatGPT incorrectly uses CAGEr; AutoBA fails at hisat2; BioMaster succeeds.

CAGE-seq	Parse & sort	Index the sorted BAM file	Peak calling	differential expression analysis
Tools	samtools/hisat2	samtools	CAGEr	EdgeR/DESeq2
ChatGPT	✓	✓	Wrongly use CAGEr	×
AutoBA	Wrongly use hisat2	×	×	×
BioMaster	✓	✓	✓	✓

Table S42: **Mass spectrometry analysis.** ChatGPT fails at normalization; AutoBA incorrectly uses limma; BioMaster completes analysis.

Mass spectrometry data analysis	Data clean	Apply normalization	identify significant expression differences	calculate fold change
Tools	pandas	numpy	scipy/lima	pandas
ChatGPT	✓	Wrongly apply normalization	×	×
AutoBA	✓	✓	✓	Wrongly use limma
BioMaster	✓	✓	✓	✓

13 Comparative Analysis: Spatial Transcriptomics

Overview

Tables S43–S49 evaluate spatial transcriptomics analyses including neighborhood enrichment, cell-type deconvolution, ligand-receptor interactions, and domain identification. BioMaster completes 4/5 tasks; ChatGPT and AutoBA complete 1/5. Cell-type deconvolution fails for all systems due to package conflicts. ChatGPT encounters Python script errors; AutoBA fails at data reading and clustering steps.

Table S43: **Spatial transcriptomics neighborhood enrichment.** ChatGPT has script errors; AutoBA has invalid response; BioMaster succeeds.

**ST - neighborhood enrichment**	**quality control**	**Normalization and Log Transformation**	**compute spatial neighborhood graph**	**neighborhood enrichment**
Tools	scanpy	scanpy	squidpy	squidpy
ChatGPT	✓	error python script	×	×
AutoBA	Invalid response in reading data	×	×	×
BioMaster	✓	✓	✓	✓

Table S44: **Spatial transcriptomics deconvolution.** All systems fail due to package conflicts and data reading issues.

ST Deconvolution	sc data preprocessing	ST preprocessing	Cell type annotation	Deconvolution
Tools	scanpy	squidpy	celltypist	tangram
ChatGPT	error python script	×	×	×
AutoBA	×, Invalid response in reading data	×	×	×
BioMaster	×, package conflicts	×	×	×

Table S45: **Spatial transcriptomics ligand-receptor analysis.** ChatGPT has script errors; AutoBA has invalid response; BioMaster succeeds.

ST Ligand-Receptor	Quality control	Normalization & Log Transform	Compute Neighborhood Graph	Ligand-Receptor Pairs & Scores
Tools	scanpy	scanpy	squidpy	squidpy
ChatGPT	✓	✓	error python script	×
AutoBA	×, Invalid response in reading data	×	×	×
BioMaster	✓	✓	✓	✓

Table S46: **Spatial transcriptomics domain identification (Part 1).** ChatGPT has Python script errors; AutoBA and BioMaster complete initial steps.

**domain identification**	**Quality control**	**Normalization and Log Transformation**	**Highly Variable Gene Selection**	**Dimensionality Reduction**
Tools	scanpy	scanpy	scanpy	scanpy
ChatGPT	✓	✓	error python script	×
AutoBA	✓	✓	✓	✓
BioMaster	✓	✓	✓	✓

Table S47: **Spatial transcriptomics domain identification (Part 2).** ChatGPT fails completely; AutoBA encounters clustering errors; BioMaster succeeds.

**domain identification**	**Compute the neighborhood graph**	**Clustering**	**Visualization (umap & mapping to tissue)**
Tools	squidpy	squidpy	squidpy
ChatGPT	×	×	×
AutoBA	✓	×, ’cluster’ key missing	×
BioMaster	✓	✓	✓

Table S48: **Complete spatial transcriptomics analysis (Part 1).** ChatGPT has script errors; AutoBA fails at preprocessing; BioMaster succeeds.

**ST Analysis**	**Preprocessing**	**Quality control**	**Normalization and Log Transformation**	**Highly Variable Gene Selection**	**Dimensionality Reduction**
Tools	scanpy	scanpy	scanpy	scanpy	scanpy
ChatGPT	✓	✓	✓	✓	wrong python script
AutoBA	×, cannot use spaceranger to do preprocessing	×	×	×	×
BioMaster	✓	✓	✓	✓	✓

Table S49: **Complete spatial transcriptomics analysis (Part 2).** Only BioMaster completes neighborhood enrichment, clustering, and variable gene analysis.

**ST Analysis**	**Compute the neighborhood graph**	**Neighborhood enrichment**	**Clustering**	**Spatial Variable Gene Analysis**	**Visualization**
Tools	scanpy	scanpy	squidpy	squidpy	squidpy
ChatGPT	×	×	×	×	×
AutoBA	×	×	×	×	×
BioMaster	✓	✓	✓	✓	✓

14 Comparative Analysis: Single-cell RNA Sequencing

Overview

Tables S50–S59 evaluate scRNA-seq workflows including clustering, differential expression, marker gene identification, cell type annotation, trajectory analysis, and regulatory network inference. BioMaster completes all 9 tasks. ChatGPT fails at quality control in most tasks (2/9). AutoBA completes basic clustering but lacks visualization and fails at advanced analyses (3/9).

Table S50: **ScRNA-seq clustering.** ChatGPT fails at quality control; AutoBA succeeds but lacks visualization; BioMaster completes full analysis.

**scRNA-seq clustering**	**Data Reading and Initialization**	**Quality Control (QC)**	**Normalization and Highly Variable Gene Selection**	**Dimensionality Reduction**	**Clustering Analysis**
Tools	scanpy	scanpy	scanpy	scanpy	scanpy
ChatGPT	✓	wrongly use scanpy to do quality control	×	×	×
AutoBA	✓	✓	✓	✓	✓, but no visualization output
BioMaster	✓	✓	✓	✓	✓

Table S51: **ScRNA-seq differential expression (Part 1).** ChatGPT fails at QC; AutoBA fails at gene selection; BioMaster succeeds.

scRNA-seq for differentially expressed genes identification	Data Reading and Initialization	Quality Control (QC)	Normalization and Highly Variable Gene Selection
Tools	scanpy	scanpy	scanpy
ChatGPT	✓	wrongly use scanpy to do quality control	×
AutoBA	✓	✓	failed to use scanpy to do highly variable gene selection
BioMaster	✓	✓	✓

Table S52: **ScRNA-seq differential expression (Part 2).** Only BioMaster completes dimensionality reduction, clustering, and differential analysis.

scRNA-seq for differentially expressed genes identification	Dimensionality Reduction	Clustering Analysis	Whole-Cell Differential Expression Analysis
Tools	scanpy	scanpy	scanpy, pandas
ChatGPT	×	×	×
AutoBA	×	×	×
BioMaster	✓	✓	✓

Table S53: **ScRNA-seq marker gene identification (Part 1).** ChatGPT fails at QC; AutoBA fails at gene selection; BioMaster succeeds.

**Single-cell data analysis Find marker genes based on count matrix**	**Data Reading and Initialization**	**Quality Control (QC)**	**Normalization and Highly Variable Gene Selection**	**Dimensionality Reduction**
Tools	scanpy	scanpy	scanpy	scanpy
ChatGPT	✓	wrongly use scanpy to do quality control	×	×
AutoBA	✓	✓	failed to use scanpy to do highly variable gene selection	×
BioMaster	✓	✓	✓	✓

Table S54: **ScRNA-seq marker gene identification (Part 2).** Only BioMaster successfully identifies and filters marker genes.

**Single-cell data analysis Find marker genes based on count matrix**	**Clustering Analysis**	**Whole-Cell Differential Expression Analysis**	**Filter marker genes**
Tools	scanpy	scanpy, pandas	pandas
ChatGPT	×	×	×
AutoBA	×	×	×
BioMaster	✓	✓	✓

Table S55: **ScRNA-seq top 5 marker genes (Part 1).** ChatGPT fails at QC; AutoBA fails at gene selection; BioMaster succeeds.

**Single-cell data analysis Identify top5 marker genes**	**Data Reading and Initialization**	**Quality Control (QC)**	**Normalization and Highly Variable Gene Selection**	**Dimensionality Reduction**
Tools	scanpy	scanpy	scanpy	scanpy
ChatGPT	✓	wrongly use scanpy to do quality control	×	×
AutoBA	✓	✓	failed to use scanpy to do highly variable gene selection	×
BioMaster	✓	✓	✓	✓

Table S56: **ScRNA-seq top 5 marker genes (Part 2).** Only BioMaster successfully identifies top 5 marker genes per cluster.

**Single-cell data analysis Identify top5 marker genes**	**Clustering Analysis**	**Whole-Cell Differential Expression Analysis**	**Find top 5 marker genes**
Tools	scanpy	scanpy, pandas	pandas
ChatGPT	×	×	×
AutoBA	×	×	×
BioMaster	✓	✓	✓

Table S57: **Complete scRNA-seq analysis (Part 1).** ChatGPT fails at QC; AutoBA and BioMaster complete basic workflow.

scRNA-seq analysis	Data Reading and Initialization	Quality Control (QC)	Normalization and Highly Variable Gene Selection	Dimensionality Reduction	Clustering Analysis
Tools	scanpy	scanpy	scanpy	scanpy	scanpy
ChatGPT	✓	wrongly use scanpy to do quality control	×	×	×
AutoBA	✓	✓	✓	✓	✓
BioMaster	✓	✓	✓	✓	✓

Table S58: **Complete scRNA-seq analysis (Part 2).** AutoBA fails at marker gene output; only BioMaster completes advanced analyses.

scRNA-seq analysis	Whole-Cell Differential Expression Analysis	Find top 20 marker genes	Cell Type Annotation	Pseudotime Trajectory Analysis
Tools	scanpy, pandas	pandas	scanpy, celltypist, pandas, matplotlib	scanpy, matplotlib
ChatGPT	✓	×	×	×
AutoBA	✓	fail in outputting marker genes file	×	×
BioMaster	✓	✓	✓	✓

Table S59: **Complete scRNA-seq analysis (Part 3).** Only BioMaster succeeds at purity, heterogeneity, and regulatory network analysis.

scRNA-seq analysis	Purity Analysis	Functional Heterogeneity Analysis	Regulatory Network Analysis
Tools	scanpy, pandas, numpy, scipy	scanpy, pandas	scanpy, decoupler, pandas
ChatGPT	×	×	×
AutoBA	×	×	×
BioMaster	✓	✓	✓

15 Comparative Analysis: Nanopore Sequencing Technologies

Overview

Tables S60–S67 evaluate Nanopore workflows including DNA methylation calling, event alignment, and comprehensive DNA sequencing (12 steps). BioMaster completes all tasks. AutoBA fails at dorado basecalling and minimap2 alignment. ChatGPT generates incorrect workflows using wrong tools (nanopolish, BWA-MEM, GATK).

Table S60: **Nanopore DNA methylation.** AutoBA fails due to incorrect dorado usage; BioMaster successfully identifies methylation.

**Nanopore Identifying DNA methylation**	**using dorado basecaller to perform modified basecalling from pod5 files and align to reference genome**	**Use dorado summary to generead-level sequencing information, including methylation details**
Tools	dorado	dorado
AutoBA	stuck due to wrongly used dorado tools	×
BioMaster	✓	✓

Table S61: **ChatGPT plan for Nanopore methylation.** ChatGPT generates incorrect workflow resulting in empty output.

**plan generated by ChatGPT**	**Basecall raw nanopore pod5 files to generate fastq reads**	**Align the basecalled reads to the reference genome**	**Convert SAM to a sorted, indexed BAM file**	**Call DNA methylation from the nanopore raw signal data and alignment information**
Tools	dorado	bwa	samtools	Megalodon
ChatGPT	empty output (due to wrong use of dorado)	×	×	×

Table S62: **Nanopore event alignment.** AutoBA incorrectly uses minimap2; BioMaster completes alignment successfully.

Nanopore Event alignment	Map the nanopore fastq file to the reference genome	Convert SAM to a sorted, indexed BAM file
Tools	minimap2	samtools
AutoBA	wrongly used minimap2 to read files	×
BioMaster	✓	✓

Table S63: **ChatGPT plan for Nanopore event alignment.** ChatGPT uses nanopolish incorrectly, generating wrong output.

plan generated by ChatGPT	index the set of FAST5 files	extract and align the raw signal data
Tools	nanopolish	nanopolish
ChatGPT	✓	wrong output due to wrong use of nanopolish eventalign

Table S64: **Nanopore DNA sequencing (Part 1).** AutoBA fails at basecalling; BioMaster completes QC and FASTQ conversion.

**Nanopore DNA seq. analysis**	**Simplex basecalling**	**Read-level summary**	**BAM to FASTQ conversion**	**FASTQ QC**
Tools	dorado	dorado	samtools	NanoPlot
AutoBA	Stuck due to wrong using of dorado tools	×	×	×
BioMaster	✓	✓	✓	✓

Table S65: **Nanopore DNA sequencing (Part 2).** Only BioMaster successfully filters reads and removes contamination.

**Nanopore DNA seq. analysis**	**Low-quality read filtering**	**Host contamination removal**	**BAM to FASTQ conversion**	**QC of filtered reads**
Tools	NanoFilter	minimap2	samtools	NanoPlot
AutoBA	×	×	×	×
BioMaster	✓	✓	✓	✓

Table S66: **Nanopore DNA sequencing (Part 3).** Only BioMaster completes reference mapping and generates statistics.

**Nanopore DNA seq. analysis**	**Reference genome mapping**	**SAM sorting and indexing**	**Alignment statistics**	**Results consolidation**
Tools	minimap2	samtools	samtools	bash
AutoBA	×	×	×	×
BioMaster	✓	✓	✓	✓

Table S67: **ChatGPT plan for Nanopore DNA sequencing.** ChatGPT plan fails at basecalling, preventing downstream analysis.

**Plan generated by ChatGPT**	**POD5 to FASTQ conversion**	**Read alignment**	**SAM to BAM conversion**	**Variant calling**	**Cross-species contamination check**
Tools	dorado	BWA-MEM	samtools	GATK	BWA-MEM
ChatGPT	Get empty output due to wrong use of dorado	×	×	×	×

16 Comparative Analysis: Comprehensive Whole Genome Sequencing

Overview

Tables S68–S71 evaluate comprehensive WGS pipelines including SNV, CNV, SV calling, annotation, and antibiotic resistance gene identification. BioMaster completes all 12 steps. AutoBA and ChatGPT fail at advanced variant calling and annotation. For SV analysis without matched normal samples, BioMaster completes all steps while AutoBA and ChatGPT achieve partial completion.

Table S68: **Comprehensive WGS analysis (Part 1).** All systems complete initial processing steps successfully.

WGS analysis	SRA to FASTQ conversion	Quality control	Read alignment	SAM to BAM conversion
Tools	fastq-dump	fastp	BWA	samtools
BioMaster	✓	✓	✓	✓
AutoBA	✓	✓	✓	✓
BioMaster-ChatGPT	✓	✓	✓	✓

Table S69: **Comprehensive WGS analysis (Part 2).** Only BioMaster completes CNV, SV detection, and SNV annotation.

WGS analysis	BAM processing	SNV calling	CNV & SV detection	SNV annotation
Tools	picard, samtools	bcftools	delly	annovar
BioMaster	✓	✓	✓	✓
AutoBA	×	×	×	×
BioMaster-ChatGPT	✓	×	×	×

Table S70: **Comprehensive WGS analysis (Part 3).** Only BioMaster completes SV annotation, VCF merging, and antibiotic resistance gene identification.

WGS analysis	CNV & SV annotation	VCF files merging	VCF to FASTA conversion	Antibiotic resistance gene identification
Tools	annovar	vcftools	bcftools, vcf2fasta	abricate
BioMaster	✓	✓	✓	✓
AutoBA	×	×	×	×
BioMaster-ChatGPT	×	×	×	×

Table S71: **WGS SV analysis without normal sample.** AutoBA and ChatGPT encounter errors; BioMaster successfully detects SVs.

**WGS SV analysis without normal sample**	**SRA to FASTQ conversion**	**Quality control**	**Alignment & SAM to BAM**	**BAM processing**	**SV detection**
Tools	fastq-dump	fastp	bwa, samtools	samtools	delly
BioMaster	✓	✓	✓	✓	✓
AutoBA	✓	×	✓	✓	×
BioMaster-ChatGPT	✓	×	✓	×	×

17 Comparative Analysis: Metagenomic Data Processing

Overview

Table S72 evaluates metagenomic analysis using R-based phyloseq workflows. AutoBA successfully completes phyloseq object creation, filtering, and visualization. ChatGPT and BioMaster encounter R script errors. This represents a domain where AutoBA demonstrates superior R package handling.

Table S72: **Metagenomic analysis.** ChatGPT and BioMaster have script errors; AutoBA successfully completes phyloseq analysis.

Metagenomic analysis	Create a phyloseq-class object	Filter the created phyloseq-class object	Plot the composition figure
**Tools**	create_phyloseq	BiocManager, devtools, phyloseq, microbiome, preprocessCore	microbiome, caret, file2meco
ChatGPT	error script	×	×
AutoBA	error script	×	×
BioMaster	✓	✓	✓

18 User Case

Setup and tasks. We recruited five novice users with no prior bioinformatics training (four undergraduates and one master's student). Each participant attempted two single-cell RNA-seq tasks: (1) clustering and (2) differential expression analysis (DEG). Participants were given only public README-level instructions and asked to iteratively refine their own prompts (e.g., "help me to do single-cell transcriptomics differential expression gene analysis by scanpy, and save the visualization results as images"; "please help me do clustering by scanpy, squidpy, and save the visualization results as images"). Unless otherwise noted, model settings followed the defaults used in the main text (e.g., main/tool model o1-2024-12-17, embedding text-embedding-ada-002).

Quantitative summary. Across participants, both tasks were completed successfully. Relative to expert references on the same datasets, clustering achieved a median concordance of ≈ 0.75 (e.g., ARI/NMI), while DEG exhibited a median top-k gene overlap of ≈ 0.85 (e.g., Jaccard). Divergences primarily reflected conservative/aggressive parameter choices (e.g., filtering thresholds, cluster resolution) rather than execution failures.

Representative prompts used by participants. help me to do single-cell transcriptomics differential expression gene analysis by scanpy, and save the visualization results as images; please help me do clustering by scanpy, squidpy, and save the visualization results as images.

Participant feedback

User A. Male, China, Sichuan Agricultural University, School of Information Engineering, Internet of Things, undergraduate.

> The user experience with this bioinformatics agent was excellent. The tool is simple to use, and the API configuration is remarkably straightforward, allowing users to get started quickly. The tool is well-designed for stability, featuring a built-in five-retry mechanism to ensure task completion. Furthermore, restarting after an interruption is very convenient and does not result in lost progress. Regarding its core functionality, the tool efficiently completed the single-cell transcriptomics differential expression analysis using Scanpy. The analysis results feature high-quality visualizations; key outputs such as the Leiden clustering plot and the volcano plot were generated clearly. The tool successfully outputted the marker genes along with their detailed metrics (e.g., logFC, p-values), providing a solid data foundation for subsequent research. A particularly commendable feature is the tool's ability to directly save visualization results as images on demand. This fully satisfies the user's need for a one-step process to acquire materials for reports. Furthermore, the selected configurations for the main model ("o1-2024-12-17") and the embedding model ("text-embedding-ada-002") were appropriate and operated smoothly. Overall, this agent tool is accurate, efficient, and easy to use, demonstrating high value in the bioinformatics analysis workflow.

User B. Male, China, Sichuan Agricultural University, School of Information Engineering, Internet of Things, undergraduate.

> Using BioMaster was extremely positive. As an undergraduate new to bioinformatics, I was surprised that I could run full single-cell workflows without understanding tool parameters, environment setup, or pipeline orchestration. I only described my goals in natural language, and BioMaster automatically planned, executed, debugged, and verified the entire workflow end-to-end with almost no intervention. All tasks (differential expression, clustering, spatial clustering, marker

identification) were executed using the following configurations: Main Model o1-2024-12-17, Tool Model o1-2024-12-17, and Embedding Model text-embedding-ada-002. The model configuration worked smoothly across the entire workflow, and the selection was flexible enough to meet performance requirements while still maintaining cost efficiency. Additionally, BioMaster supported both local deployment and API-based execution modes, enabling flexible usage depending on available resources and computational environments. During execution, all four prompts achieved a 100% completion rate. The outputs were stable, deterministic, and highly reproducible. Each task produced structured standardized artifacts, visualizations, and summary metrics that were directly suitable for downstream biological interpretation and scientific research usage. The Gradio UI significantly lowered the barrier to use — users only needed to provide Goal, Data List, Model selection, and execution mode, and the system handled everything else transparently. Runtime visualization of plans, step scripts, debug traces, and results further enhanced user trust and controllability. Compared to traditional prompt→error→debug→rerun loops, BioMaster completely removed the initial barrier that usually blocks beginners like me at the very first step. The system provided stable structured outputs, visualizations, and verified standardized files ready for interpretation. The live UI plan visualization + debug trace made me feel safe and in control, even though I do not yet fully understand every technical detail. BioMaster preserved full logs and fully reproducible shell scripts, allowing me to inspect exactly what was executed, and optionally re-run the generated .sh files manually for learning or debugging. For me, BioMaster transformed single-cell analysis from a multi-year skill barrier into something I can reliably execute today — even at the undergraduate beginner stage.

User C. Female, China, Sichuan Agricultural University, School of Information Engineering, Computer Science and Technology, undergraduate.

As an undergraduate majoring in computer science who has just started learning bioinformatics, I initially found it quite challenging to get started — there were so many tools, parameters, and workflows to understand, and even running a simple analysis often felt overwhelming. When I first tried to analyze single-cell data manually, I constantly ran into dependency errors and parameter issues. Using BioMaster completely changed that experience. My overall workflow became incredibly smooth, and I was finally able to focus on understanding the biology instead of struggling with technical setup. I was also pleasantly surprised by BioMaster's flexibility in model configuration and technical architecture. It supports multiple large language models such as OpenAI, DeepSeek, and Claude, and even allows running local Ollama models smoothly. Its dual RAG knowledge base design (PLAN RAG and EXECUTE RAG) is particularly clever — it keeps planning knowledge and execution knowledge separated, ensuring precise retrieval and avoiding confusion. On top of that, BioMaster supports over 30 common bioinformatics analysis types, including single-cell, RNA-seq, and WGS/WES, basically covering everything I might need as a beginner in the field. BioMaster also excels in visualization and reproducibility. It automatically generates key plots and result files during analysis and supports a checkpoint resume mechanism, so if something fails, it can continue from the last successful step instead of starting over — a huge time-saver. It also keeps detailed logs and shell scripts, letting me review exactly what was executed, which helps me both learn and troubleshoot. The Gradio GUI is especially friendly for newcomers, while the command-line mode is great for more advanced users. During my tests, I asked it to generate visualization outputs in PNG, JPG, and PDF formats — all worked perfectly. What really amazed me was when I requested an HTML report: it automatically created a complete research report that was detailed and well-organized, even covering aspects I hadn't considered. Overall, BioMaster delivers accurate, well-structured, and efficient outputs. The generated analysis reports and scripts are clean, parameterized properly, and can be directly used for publication or downstream analyses. The interface is intuitive and user-friendly, allowing me to complete tasks that used to take hours or days in a fraction of the time. For someone like me who's just stepping into bioinformatics, BioMaster is truly both smart and practical — it not only saves time but also helps me genuinely understand how a full analysis pipeline works.

User D. Male, China, Sichuan Agricultural University, School of Information Engineering, Computer Science and Technology, undergraduate.

As a beginner in bioinformatics, I have greatly benefited from BioMaster. Traditional analysis often fails due to errors in manually assembling command-line tools, making it inefficient. BioMaster transforms this complexity into a simple "task description," significantly lowering the learning threshold for those without deep specialized backgrounds. The clear configuration files and friendly Gradio UI make it immediately accessible, allowing me to focus more on interpreting biological data. Tasks that previously took days now complete in hours with excellent analysis quality. The system's reliability and efficiency comes from its superior engineering architecture, centered on the Multi-Agent Collaboration System. By deploying specialized Agents (PLAN, TASK, DEBUG, CHECK), BioMaster mitigates the common "error propagation problem" in long pipelines. The DEBUG Agent is particularly impressive—it intelligently analyzes error types and generates targeted solutions rather than simply retrying. Additionally, extensive model compatibility (OpenAI, Claude, local Ollama) provides crucial flexibility in balancing data privacy and computational budgets. Besides, BioMaster is extremely practical. It offers both fully automated and manual review modes—I can learn commands line by line in manual mode before switching to automation for efficiency. The detailed debug_output logs and preserved shell scripts make the entire process transparent, providing vital documentation for methodology sections and ensuring reproducibility. In summary, BioMaster is a useful tool that applies advanced agent technology to deliver a reliable, efficient, and user-friendly solution for bioinformatics.

User E. Female, China, University of Electronic Science and Technology of China, School of Information and Software Engineering, Software Engineering, Master's student.

The feedback for using the bioinformatics agent is highly positive. The tool effectively completed the task of single-cell transcriptomics differential expression gene analysis using Scanpy, as requested. The key visualizations, such as the Leiden clustering plot and the volcano plot, were generated without any issues. The tool also successfully outputted the marker genes, providing detailed information on each gene, including scores, logFC, p-values, and adjusted p-values. The model configurations, such as selecting the main model ("o1-2024-12-17") and embedding model ("text-embedding-ada-002"), were appropriately set and worked seamlessly throughout the analysis. The API configurations were also correctly implemented, ensuring smooth integration with the remote services for data retrieval and processing. The tool was able to process the provided single-cell data and generate the required visualizations efficiently. One of the standout features was the tool's ability to save the visualization results as images, as requested. This feature is particularly useful for users who need to present or further analyze the visual output. The user's goal of having the results saved directly from the tool without extra steps was met successfully, and the output images were stored in the expected formats, ready for immediate use. In terms of the tool's overall performance, the outputs were both accurate and timely, and the process was user-friendly. The clear and structured format of the results, with essential metrics such as logFC and p-values included for each gene, provides valuable insights for downstream analyses. The visualizations were not only informative but also aesthetically pleasing, enhancing the overall quality of the analysis.
