# Supplementary material for: BioMaster: Multi-agent system for automated bioinformatics analysis workflow
Source: Patterns (N Y). 2026 Jul 7;7(8):101611. doi: 10.1016/j.patter.2026.101611 (PMC13494596; doi:10.1016/j.patter.2026.101611)
Supplement: Supplementary file 2 [file mmc2.pdf]

Patterns

Article

BioMaster: Multi-agent system for automated bioinformatics analysis workflow

Highlights

- BioMaster is a multi-agent system for automated bioinformatics workflows
- BioMaster integrates a dual RAG design for planning and execution knowledge
- BioMaster is evaluated on a wide range of bioinformatics tasks spanning 102 tools

Authors

Houcheng Su, Junning Feng, Yawen Lu, ..., Yusen Hou, Tingyu Zhu, Yanlin Zhang

Correspondence

yanlinzhang@hkust-gz.edu.cn

In brief

BioMaster is a knowledge-guided multi-agent framework that automates bioinformatics workflow planning, execution, debugging, and validation. Across 49 tasks and 102 tools, it improves workflow completion over existing automated systems and supports both proprietary and open-source language models.

Su et al., 2026, Patterns *7*, 101611
August 14, 2026 © 2026 The Author(s). Published by Elsevier Inc.
https://doi.org/10.1016/j.patter.2026.101611

CellPress

Patterns

CellPress
OPEN ACCESS

Article

BioMaster: Multi-agent system for automated bioinformatics analysis workflow

Houcheng Su,[1,3] Junning Feng,[1,3] Yawen Lu,[1] Yucheng Xu,[1] Jinming Yang,[1] Haojie Lu,[1] Jixin Yang,[1] Xu Yang,[1] Sirui Xie,[1] Weicai Long,[1] Chengrui Wang,[2] Yusen Hou,[1] Tingyu Zhu,[1] and Yanlin Zhang[1,4,*]
[1]Data Science and Analytics Thrust, Hong Kong University of Science and Technology (Guangzhou), Guangzhou, Guangdong, China
[2]Guangdong Hospital of Traditional Chinese Medicine, Guangzhou, Guangdong, China
[3]These authors contributed equally
[4]Lead contact
*Correspondence: yanlinzhang@hkust-gz.edu.cn
https://doi.org/10.1016/j.patter.2026.101611

THE BIGGER PICTURE Bioinformatics has become central to modern life sciences, yet many analyses still depend on complex, multi-step workflows that integrate diverse software tools and heterogeneous data formats. Despite advances in workflow managers, researchers are often required to manually select methods, configure parameters, and resolve software dependencies. This process is time-consuming, error-prone, and difficult to reproduce, creating a persistent barrier for researchers without strong computational backgrounds and limiting the scalability of biological data analysis. Recent advances in large language models (LLMs) have created new opportunities for automating scientific workflows through natural language interaction. This work introduces a knowledge-guided, multi-agent framework that leverages LLMs to automate the construction and execution of bioinformatics workflows from high-level task descriptions. By combining structured workflow planning with retrieval of tool-specific knowledge, the system reduces the need for manual pipeline engineering while maintaining flexibility across diverse analysis tasks.

SUMMARY

The growing volume and complexity of biological data have made bioinformatics workflows increasingly labor-intensive, error-prone, and difficult to scale. Large language model-based agents offer potential for automation but often fail in complex, multi-step analyses because of limited robustness. We present BioMaster, a multi-agent framework that integrates workflow planning, execution, error recovery, and output validation. BioMaster incorporates a dual retrieval-augmented design to leverage domain knowledge for tool selection, parameterization, and adaptation across tasks. A dedicated debug agent supports real-time error detection and correction, while memory optimization enables long, multi-stage workflows. In benchmarking across 49 bioinformatics tasks spanning 102 tools, BioMaster completed substantially more workflows than did existing automated systems, particularly in complex, interdependent pipelines. BioMaster supports both proprietary and open-source language models, enabling flexible deployment across different computational settings.

INTRODUCTION

Advances in high-throughput technologies such as whole-genome sequencing (WGS),[1] single-cell transcriptomics,[2] and 3D genome mapping[3] have dramatically expanded the volume and diversity of biological data.[4–6] Extracting biological insight from these data requires multi-step computational workflows that integrate numerous software tools, from standard preprocessing—such as read alignment and quality control—to specialized downstream analyses including chromatin loop detection, topologically associating domain annotation,[7–9] spatial domain segmentation, and microbial community profiling. While standardized solutions exist for certain early-stage tasks, downstream analyses remain fragmented, often lacking consensus "gold standard" tools. Researchers must therefore manually identify and evaluate alternative methods, resolve software dependencies, and interpret heterogeneous outputs. These processes are labor-intensive, error prone, and sensitive to computational environment inconsistencies,[10,11] posing significant barriers for researchers without advanced computational expertise and limiting scalability and reproducibility.

This is an open access article under the CC BY license (http://creativecommons.org/licenses/by/4.0/).

Workflow management systems such as Snakemake[12] and Nextflow[10,11] provide robust frameworks for executing bioinformatics pipelines but require substantial scripting and configuration skills. Web-based platforms including Galaxy,[13] iDEP,[14] and ICARUS[15] offer more accessible interfaces but often at the expense of flexibility, limiting their applicability to highly customized analyses and raising potential data privacy concerns. Crucially, both categories still depend on user expertise for tool selection, parameter tuning, and troubleshooting, leaving a significant automation gap.

Recent advances in AI, particularly large language models (LLMs),[16,17] have created opportunities to automate scientific workflows via intelligent agent-based systems. Domain-specific agents have demonstrated promising results in fields such as chemistry and molecular design, exemplified by ChemCrow[18] and MultiMol,[19] which autonomously propose synthetic routes, conduct virtual experiments, and optimize molecular structures. Knowledge-graph-driven systems such as SciToolAgent show that graph-based retrieval can intelligently orchestrate hundreds of tools with built-in safety checks across biology, chemistry, and materials science.[20] In bioinformatics, AutoBA[21] showcased LLM-driven automation for multi-omics workflow planning. However, as a single-agent system, AutoBA focuses on pipeline design and struggles with executing complex, interdependent tasks, robust error detection, and recovery. Multi-agent architectures offer a pathway to overcome these limitations by delegating specialized responsibilities—such as planning, execution, and validation—to dedicated agents. Tools such as CellAgent[22] and other single-cell-focused systems[23] illustrate the potential of role-specific agents, but most existing frameworks target narrow application domains and lack the versatility to manage diverse modalities such as epigenomics, proteomics, and population genetics. CRISPR-GPT[24] demonstrates fully AI-guided CRISPR design and data analysis with experimental validation across knockout and epigenetic activation assays. Technical barriers also persist: long and complex workflows challenge LLMs' memory management[16]; reliance on static, manually curated knowledge limits adaptability in rapidly evolving fields, and the absence of robust, automated error recovery mechanisms risks error propagation and undermines analytical integrity. In addition, many existing systems rely heavily on proprietary models such as GPT-4, raising concerns regarding transparency, reproducibility, accessibility, and long-term sustainability.

Here, we present BioMaster, a knowledge-enabled multi-agent framework for fully automated biological data analysis. BioMaster integrates four specialized agents within a decision-making system: a plan agent for workflow design, a task agent for command generation and execution, a debug agent for real-time error detection and correction, and a check agent for rigorous output validation. Central to BioMaster's adaptability is a dual retrieval-augmented generation (RAG) system that dynamically queries continuously updated, domain-specific knowledge repositories containing curated workflows, detailed tool documentation, and methodological best practices. We evaluated BioMaster across 49 tasks spanning multiple omics modalities—including transcriptomics, epigenomics, metagenomics, and 3D genome architecture—covering 102 bioinformatics tools. BioMaster autonomously executed multi-step workflows, effectively recovered from execution errors, and outperformed existing automated systems in versatility, robustness, and reproducibility. By unifying planning, execution, error correction, and validation, BioMaster reduces manual workload, promotes methodological consistency, and enables scalable, accessible bioinformatics analyses across a broad spectrum of research applications.

RESULTS

BioMaster is a crew of agents for biological data analysis

We developed BioMaster, a multi-agent framework that autonomously translates natural language task descriptions into executable bioinformatics workflows (Figure 1). It comprises four specialized agents—plan, task, debug, and check—that operate in an iterative loop to plan, execute, monitor, and validate each step of the analysis. Each agent is role based, with its functionality guided by a dedicated prompt. This process is supported by a dual RAG architecture that dynamically injects expert knowledge at key decision points. The plan RAG module provides high-level methodological knowledge, such as common analysis strategies and workflow templates, which the plan agent uses to generate a step-by-step analysis plan. Each step specifies the required inputs and outputs and the appropriate software tools. The execute RAG, in contrast, supplies low-level technical details—including installation commands, configuration parameters, and command-line examples—that inform the task and debug agents during execution and error handling. While the dual RAG design improves performance on complex bioinformatics workflows, it requires an upfront knowledge curation step. This process is knowledge based rather than code based, involving the provision of documentation, example commands, and expected input-output specifications. In our study, adding a new tool typically requires less than 10 min when complete documentation and straightforward installation are available or ∼30 min for more complex setups; the longest case did not exceed 1 h. This one-time effort amortizes quickly across repeated analyses and users.

Workflow execution begins with the plan agent, which formulates a structured analysis plan based on the user's objective and retrieved domain knowledge. The task agent then converts each planned step into executable scripts by incorporating detailed usage instructions from execute RAG. These scripts are executed within a Conda-managed environment or a Docker container (supplemental methods: "Docker validation"). The debug agent continuously monitors for runtime failures. When errors occur, it parses diagnostic messages and queries execute RAG for targeted troubleshooting guidance, such as resolving missing dependencies or correcting invalid arguments. To manage context efficiently, the debug agent maintains compact summaries of previous diagnostic attempts, preserving essential information while minimizing token usage. After each step is executed, the check agent verifies the outputs against the planned specifications—ensuring completeness and format conformity. If discrepancies are found, corrective actions are triggered before moving on to the next step. For tools or tasks absent from the curated knowledge base (KB; cold-start scenarios), BioMaster can proceed using the backbone model's general knowledge; however, this mode is less reliable.

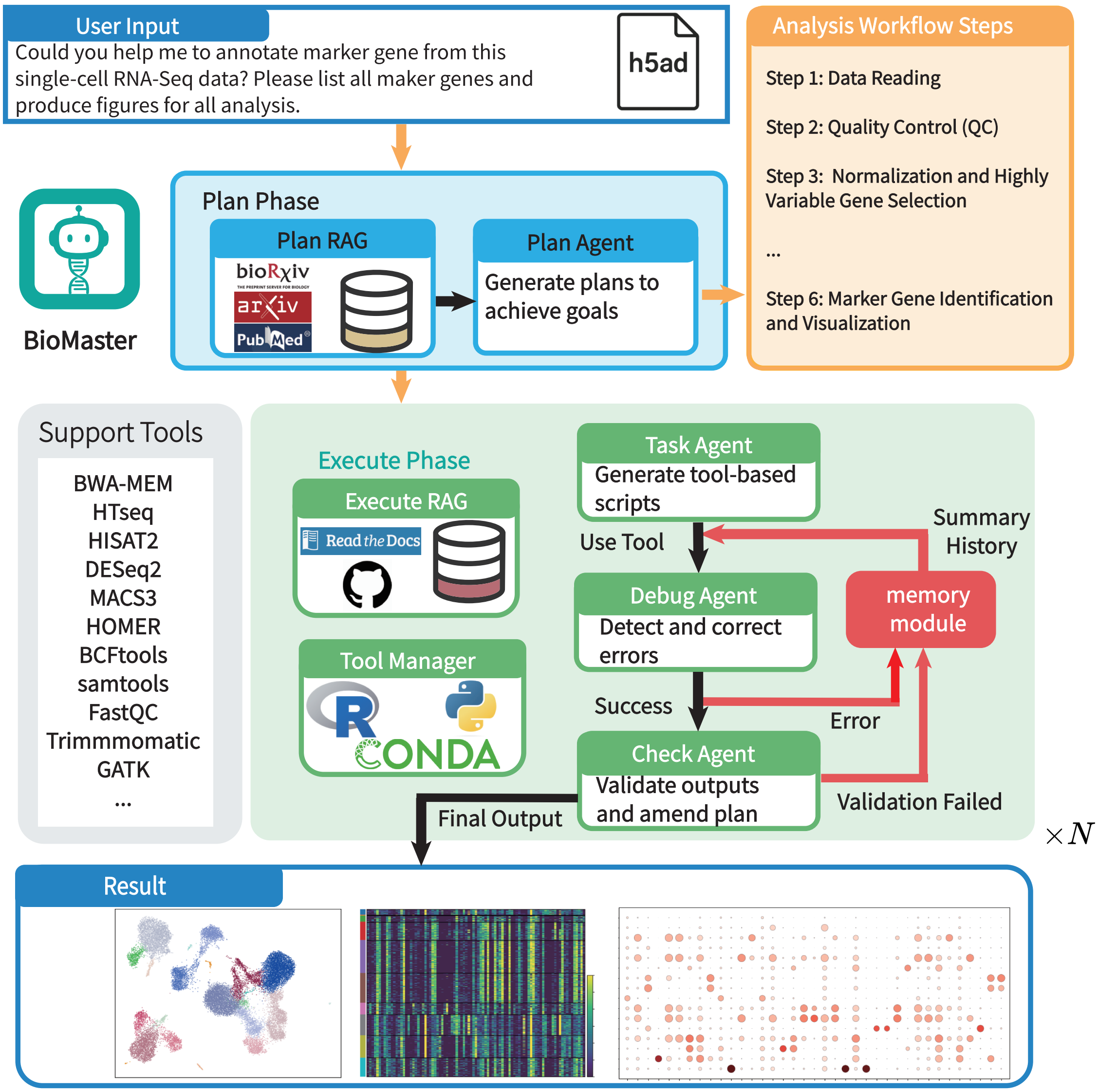

Figure 1. Overview of BioMaster

The system receives a user-defined analysis goal (example shown for single-cell marker gene identification and visualization) and generates an executable workflow. In the planning phase, the plan agent queries the plan RAG knowledge base to retrieve relevant methodological workflows and outputs a structured, stepwise plan. In the execution phase, the task agent uses execute RAG to generate and run tool-specific scripts, while the debug agent monitors execution, resolves errors using retrieved troubleshooting information, and maintains a condensed summary of diagnostic history. The check agent validates outputs at each step, amends the plan if discrepancies are detected, and triggers re-execution when necessary. This cycle repeats until all steps are successfully completed.

To support coherent inter-agent reasoning, BioMaster performs summarization after every step, recording the task outcome, relevant feedback, and any workflow adjustments. These summaries streamline downstream decisions by providing context in a compact form.

These agents operate in a coordinated loop—planning, executing, validating, and adjusting—until the full workflow is completed. By combining specialized agents with modular retrieval and context management, BioMaster enables robust execution of complex bioinformatics workflows directly from natural language instructions.

To assess real-world usability and mitigate developer-prompt bias, we recruited five participants without bioinformatics expertise (four undergraduates and one master's student) for a user study. Each participant followed only the tutorial materials referenced in the data and code availability (installation, configuration, and quick-start usage) and was asked to complete two single-cell RNA sequencing (scRNA-seq) tasks—clustering and differential expression—by iteratively formulating natural language goals. scRNA-seq was selected because it yields interpretable visualizations that enable objective evaluation without specialized domain knowledge. We also collected structured

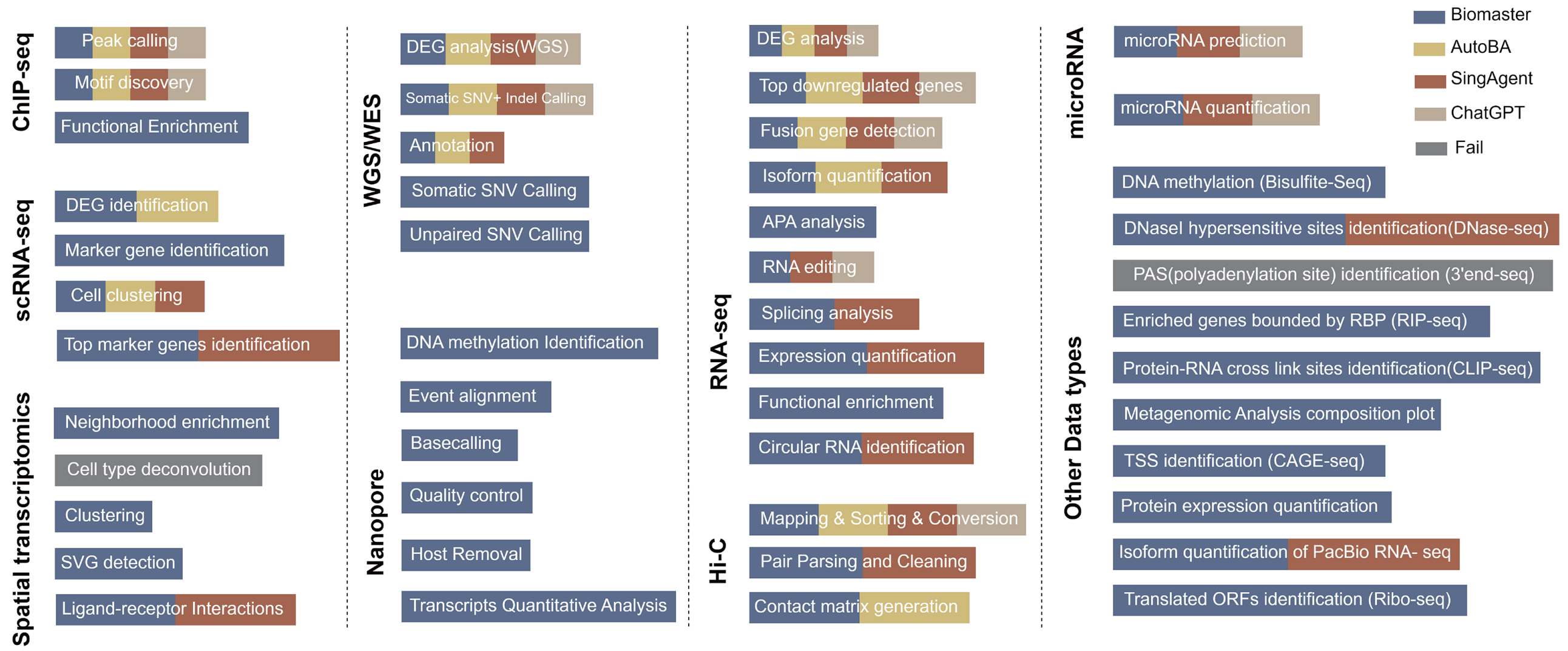

Figure 2. Task coverage of BioMaster, AutoBA, and ChatGPT across bioinformatics workflows

Each rectangle represents an analytical task by data type, with colors indicating which system completed it end to end without human intervention. BioMaster shows substantially broader coverage across modalities than AutoBA and ChatGPT.

user feedback on usability and clarity (supplemental methods: "user case"). Outputs were compared against results produced by an expert. Clustering achieved a median concordance of approximately 0.75, as measured by the adjusted Rand index (ARI) and normalized mutual information (NMI), with uniform manifold approximation and projection (UMAP) structure broadly consistent with the expert solution; differential expression reached roughly 0.85 overlap with expert-selected top genes. Discrepancies arose mainly from parameter choices (e.g., filtering thresholds and clustering resolution) rather than execution failures, indicating that BioMaster can automate pipelines and reduce user effort by shifting attention from workflow execution to parameter refinement.

BioMaster is a versatile agent for analyzing a wide spectrum of bioinformatics tasks

To assess the versatility of BioMaster, we benchmarked its performance across 49 tasks (Table S2). These workflows encompassed both routine and advanced analyses across genomics, transcriptomics, epigenomics, spatial omics, metagenomics, and proteomics. We compared BioMaster to two baselines: ChatGPT, a general-purpose LLM, and AutoBA, a single-agent pipeline executor. Given the scale of the benchmark, systematic comparison with expert-curated results is impractical. As BioMaster and alternative tools are all designed to automate bioinformatics workflows at the level of tool execution rather than assess biological validity, we evaluate performance by workflow execution success.

BioMaster successfully completed 47 out of 49 tasks (95.9%), compared with 24 (49.0%) for SingleAgent, 13 (26.5%) for AutoBA, and 12 (24.5%) for ChatGPT. We configured AutoBA to use the same tool-level execute KB as BioMaster, ensuring consistent access to tool knowledge across methods. The SingleAgent baseline received a single merged KB (plan+execute) to test whether access to the same information—without multi-agent orchestration—closes the gap; ChatGPT ran without an external KB (methods). The SingleAgent baseline improved over the no-KB (without any KB) baseline but remained substantially below BioMaster, with failures concentrated in later, interdependent stages where merged retrieval often surfaced high-level workflow text when fine-grained tool parameters were required and where the absence of specialized debug/check agents limited recovery. Execution logs indicated that 40 workflows invoked the plan RAG module for task-level planning, and 183 of 275 analytical steps accessed the execute RAG module for tool-specific knowledge retrieval. All systems required manual environment setup for R-based packages due to unresolved dependency conflicts—a common challenge in bioinformatics. Despite this shared limitation, BioMaster demonstrated greater robustness by autonomously handling additional technical issues that caused failure in the baseline systems. Figure 2 summarizes completion status across systems, highlighting BioMaster's consistency across diverse tasks; Table 1 offers a qualitative side-by-side comparison of automation, versatility, error correction, robustness, and token efficiency. To quantify computational overhead, we further analyzed five representative tasks that all systems could complete (single-cell marker identification, clustering, differential expression, chromatin immunoprecipitation [ChIP]-seq peak calling, and motif discovery). We measured total input tokens, output tokens, working time (wall-clock execution), and thinking time (LLM API latency). The detailed results can be found in Figures S6 and S7.

In standard tasks, such as Hi-C data alignment (Table S6), ChIP-seq peak calling (Table S9), and WGS preprocessing (Tables S22 and S23), all three systems performed comparably. However, as task complexity increased, baseline systems showed a substantial drop in completion rates. For example, in bulk RNA-seq analysis, while all systems handled differential expression (Table S16), only BioMaster consistently completed advanced tasks including alternative polyadenylation analysis (Tables S14 and S15), transcript quantification (Table S32), functional enrichment (Table S33), circular RNA detection

CellPress
OPEN ACCESS

Table 1. A comparison of bioinformatics workflow execution methods

Methods	Automation	Versatility	Error correction	Robustness	Token efficiency
BioMaster	✓✓✓✓	✓✓✓✓	✓✓✓✓	✓✓✓✓	✓✓✓
AutoBA	✓✓✓	✓✓✓	✓✓✓	✓✓✓	✓
Conventional tools	✓	✓	✓	✓✓	–
Online webserver	✓✓	✓	✓	✓✓	–
ChatGPT	✓✓	✓✓	✓✓	✓✓	✓✓
Open-source LLMs	✓✓	✓	✓	✓	✓✓

Each method was evaluated on degree of automation, versatility, error correction, robustness, and token-saving based on qualitative observation of their capabilities.

(Table S34), and RNA splicing (Table S38). Among these, the alternative polyadenylation workflow highlighted a clear performance gap: BioMaster successfully resolved all dependencies and handled intermediate files correctly, whereas AutoBA failed due to missing annotations, and ChatGPT produced invalid scripts. A similar pattern emerged in splicing analysis: BioMaster generated an executable pipeline end to end, while ChatGPT failed midway through execution, and AutoBA failed to produce essential outputs.

In single-cell transcriptomics, BioMaster was the only system to complete end-to-end workflows, encompassing quality control, clustering, marker gene identification, and differential expression (Tables S50–S59). AutoBA completed clustering but failed in subsequent stages, while ChatGPT consistently failed during preprocessing and initial quality assessment. A similar trend was observed in spatial transcriptomics: BioMaster successfully executed neighborhood enrichment, spatial clustering, and ligand-receptor interaction analysis. In contrast, AutoBA encountered parsing errors (e.g., "key missing" during clustering), and ChatGPT was unable to load spatial data for neighborhood analysis (Tables S43–S49).

In epigenomics, BioMaster completed full Hi-C workflows—from raw read alignment through pair parsing, filtering, and contact matrix generation. AutoBA and ChatGPT completed only the initial mapping and sorting steps, failing in downstream stages that require interdependent tool usage and format-specific handling (Tables S4–S8). These results underscore the strength of BioMaster's multi-agent system in coordinating complex, tool-specific processes that conventional systems struggle to manage. BioMaster also demonstrated strong performance in long-read sequencing. For Nanopore DNA data, it completed workflows involving base calling, quality control, methylation detection, and event alignment. AutoBA encountered file-handling errors during base calling, whereas ChatGPT generated incorrect methylation detection outputs. Similarly, for long-read transcriptomics from PacBio and Nanopore platforms, BioMaster completed isoform quantification pipelines, whereas AutoBA applied incorrect tools, and ChatGPT failed to parse the input formats (Tables S60–S67).

In specialized RNA-seq modalities such as crosslinking-immunoprecipitation sequencing (CLIP-seq), RNA immunoprecipitation sequencing (RIP-seq), ribosome profiling sequencing (Ribo-seq), genome architecture and gene expression sequencing (GAGE-seq), and cap analysis of gene expression sequencing (CAGE-seq), BioMaster outperformed both baselines. In CLIP-seq, it correctly identified protein-RNA cross-linking sites through a multi-step process, while AutoBA returned an "invalid response" error, and ChatGPT failed to properly use CLIPper (Table S35). In CAGE-seq, BioMaster accurately identified transcription start sites, while both ChatGPT and AutoBA misused inappropriate tools (Table S41). BioMaster also succeeded in complex metagenomic and proteomic analyses. In metagenomics, it performed full microbial community profiling, while both baselines failed to complete the compositional analysis (Table S72). In proteomics, BioMaster executed mass spectrometry-based workflows for protein quantification, overcoming configuration and dependency issues that caused AutoBA and ChatGPT to fail (Table S42).

To further analyze workflow behavior beyond end-to-end completion rates, we examined the procedural quality of the generated plans and scripts. Given the scale of the benchmark and the heterogeneity of tasks, manually inspecting execution artifacts across all workflows would require substantial expert effort and is not feasible in practice. We therefore adopt an automated assessment in which LLMs are used as independent raters to review execution-related properties of workflows, following recent practice in large-scale system evaluation.[25] Specifically, this assessment was applied to the subset of tasks that all systems could complete (12 tasks; five runs per system). Five LLMs (deepseek_v3_1_250821_thinking, gpt_5_2025_08_07, gemini_2_5_pro_nothinking, gpt_5_2_2025_12_11, and qwen3_max) were used as raters to score the generated plans and scripts along several execution-oriented dimensions, such as plan completeness, script quality, error handling, etc. Scores were aggregated across runs and raters to obtain system-level summaries. As shown in Figure 4A, BioMaster achieves higher scores than alternative systems across these dimensions. The full assessment prompts and per-rater results are provided in Figures S1–S5 and Listing S1. To assess agreement among these raters, we quantified inter-rater reliability using the intraclass correlation coefficient (ICC). Across the seven scoring dimensions, evaluator-averaged reliability was moderate to high, with ICC(3,5) values ranging from 0.70 to 0.92 and higher agreement for more concrete criteria such as task completion (supplemental methods: "multi-rater LLM evaluation protocol").

In summary, BioMaster demonstrated broad versatility and robustness across a wide range of bioinformatics tasks and data types. Its superior performance stems from its multi-agent architecture, modular task decomposition, and integration of retrieval-augmented domain knowledge—establishing it as a reliable and generalizable framework for automated biological data analysis.

BioMaster excels across established and emerging bioinformatics tools

Existing methods, including AutoBA and ChatGPT, perform reliably with widely used, well-documented software but often fail when workflows require complex, under-documented, or newly released tools. To assess performance across this spectrum, we benchmarked BioMaster, AutoBA, and ChatGPT on 102 bioinformatics tools spanning established utilities, specialized domain software, and recently developed packages.

For widely used tools with standardized interfaces and comprehensive documentation—such as FastQC,[26] Trimmomatic,[27] HISAT2,[28] Samtools,[29] and GATK[30]—all three systems generated syntactically valid commands, reflecting the accessibility of well-supported tools to both language model- and template-based systems.

Performance diverged markedly for tools requiring complex parameterization, multi-stage execution, or environment-specific configurations. ChatGPT, although often producing syntactically correct commands, frequently failed due to incomplete arguments, unresolved dependencies, or misinterpreted usage requirements, as with pairtools and cooler for Hi-C processing, phyloseq[31] for metagenomic classification, and BEDTools[32] for genomic interval operations. AutoBA performed better for tools with explicit, well-documented workflows but struggled with environment customization or cross-tool integration. Failures included assay for transposase-accessible chromatin using sequencing (ATAC-seq) workflows using BEDTools (incorrect input formatting); genome assembly with minimap2[33] and dorado (unresolved dependencies); and analyses using bowtie2,[34] miRDeep2,[35] or BWA[36] (Conda installation conflicts). AutoBA also misapplied Trim Galore[37] in polyadenylation site identification, indicating poor tool-task alignment.

In contrast, BioMaster maintained high success rates across both standard and complex tools. Leveraging its dual RAG framework, it retrieved and parsed official documentation, GitHub repositories, and user-provided resources to generate executable, context-specific commands. In scRNA-seq, it ran pipelines involving Pandas,[38] Scanpy,[39] and Cell Ranger,[40] correctly configuring inputs, clustering parameters, and visualization outputs. In Hi-C analysis, it executed complete pairtools-based workflows with appropriate resolution and normalization, while both baselines failed. In proteomics, it applied Numpy[41] and Scipy[42] for mass spectrometry data normalization, avoiding the misapplications seen in AutoBA and ChatGPT. For Nanopore sequencing, it successfully completed the Nanopolish workflow, managing installation, indexing, and event alignment—tasks where both baselines failed due to command-line interface (CLI) errors or dependency mismanagement.

We further tested adaptability to recently released, minimally documented tools: GeneFEAST,[43] MAFin,[44] Rnalib,[45] nichePCA,[46] and WGATools.[47] Selected for methodological diversity, recency, and absence from pre-training corpora, these tools challenge static, template-based approaches. BioMaster successfully executed all five by retrieving and parsing source documentation, configuring environments, and generating correct outputs. AutoBA completed two (GeneFEAST and WGATools), while ChatGPT succeeded only on GeneFEAST. Baseline failures stemmed from syntax errors, incorrect parameters, or unresolved dependencies—for example, AutoBA's inability to configure Rnalib (specialized Conda packages) and failure to run MAFin (incompatible CLI syntax). BioMaster configured Rnalib directly from repository instructions and executed MAFin with multithreaded motif search and correct output formatting.

Collectively, these results demonstrate BioMaster's ability to operate across the full spectrum of bioinformatics tools. By integrating retrieval-augmented reasoning with dynamic environment configuration, it bridges the gap between static LLM capabilities and the rapidly evolving bioinformatics software ecosystem, enabling autonomous, reproducible analysis across both established and emerging tools.

BioMaster outperforms alternative systems in multi-step biological data analysis tasks

We benchmarked BioMaster, AutoBA, and ChatGPT on four computationally intensive workflows—Hi-C chromatin conformation (9 steps), scRNA-seq (10 steps), spatial transcriptomics (9 steps), and WGS/whole-exome sequencing (WES; 12 steps)—each requiring sequential execution of heterogeneous tools, complex file transformations, and dynamic parameter tuning. Each workflow was executed five times per system on identical datasets, with stage-level success (0–5 completions) recorded to assess both performance and reproducibility (Figure 3).

BioMaster achieved the highest completion and reproducibility rates across all workflows, particularly in later stages where tool interoperability and parameter optimization were critical. In Hi-C analysis, it completed all stages in all runs, whereas AutoBA stalled after initial file operations, and ChatGPT failed during matrix construction. In scRNA-seq, BioMaster maintained perfect preprocessing success (5/5) and $\geq$4/5 in advanced analyses; AutoBA matched early-stage performance but dropped sharply downstream, and ChatGPT failed after quality control. In spatial transcriptomics, only BioMaster consistently progressed through clustering (4/5), spatial visualization (3/5), and spatial expression pattern analysis (3/5), with both baselines failing in these tasks. In WGS/WES, BioMaster completed all 12 stages in all runs, whereas AutoBA and ChatGPT succeeded only in early processing before failing in variant annotation and interpretation.

BioMaster also supports user intervention at any stage of execution without restarting workflows, allowing parameters or tools to be modified and execution to resume from the same point. This flexibility, absent in alternatives, reduces redundant computation and makes BioMaster well suited for iterative and exploratory analyses.

BioMaster enables autonomous error correction in complex bioinformatics workflows

We evaluated the impact of BioMaster's debug agent—which works with the execute RAG module to detect, diagnose, and recover from execution errors—by comparing BioMaster (referred to as the full system) to a variant without this debug agent. Four representative workflows were tested—ChIP-seq peak calling, somatic variant detection in WGS/WES, Hi-C contact matrix construction, and RNA-seq differential expression—each executed five times, with success recorded at each analytical step (Figure 4B). Across all workflows, the debug agent

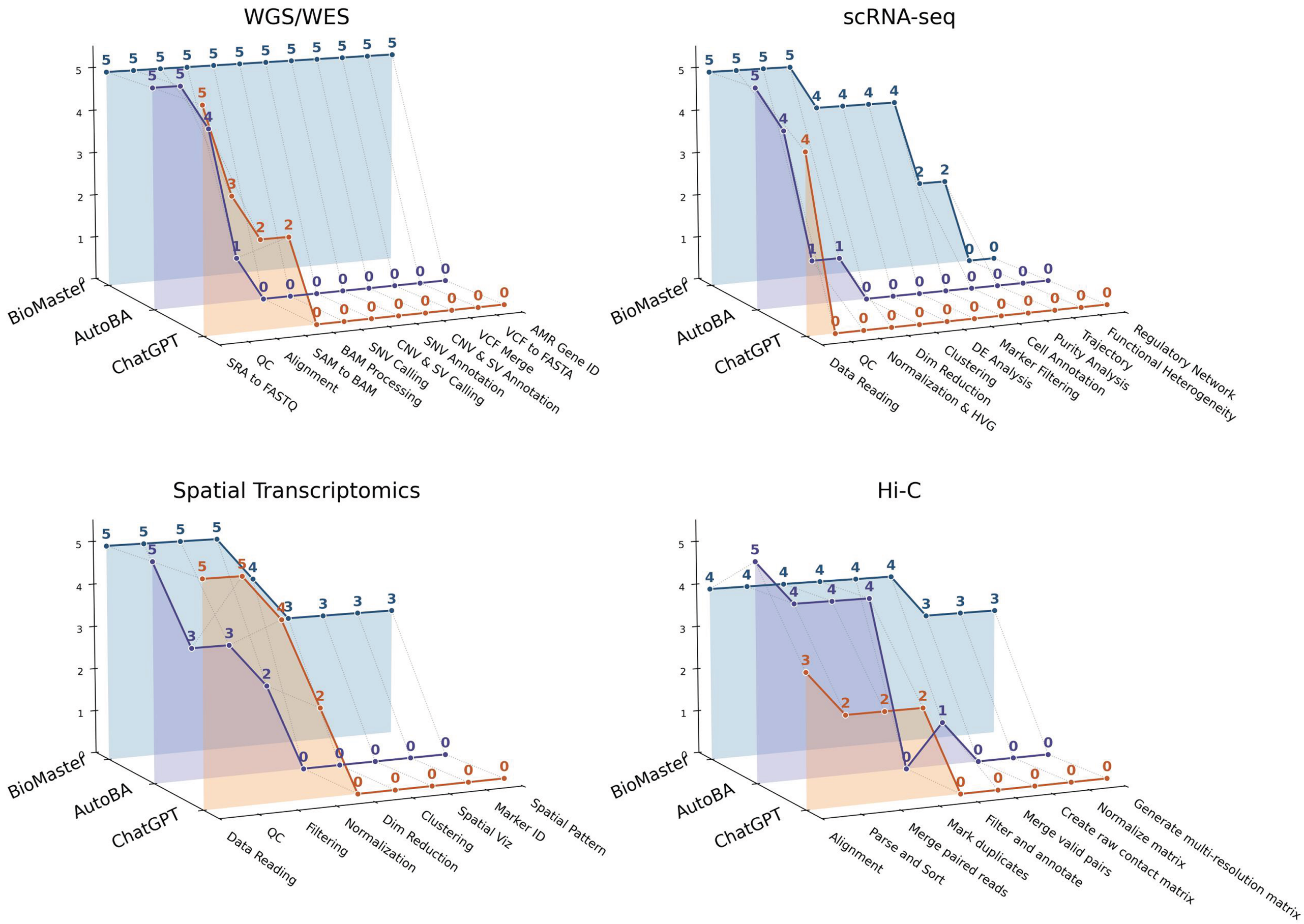

Figure 3. Execution success across multi-step bioinformatics workflows

Execution success rates (0–5, across five trials) are shown for BioMaster (blue), AutoBA (purple), and ChatGPT (red) on four representative workflows: Hi-C, single-cell RNA–seq, spatial transcriptomics, and WGS/WES. Each plot tracks the number of successful runs per step, highlighting BioMaster's higher completion rates across full workflows compared to the alternatives.

substantially increased completion rates by preventing early-stage errors from propagating downstream (Table S3). In ChIP-seq, the full system completed all five stages in every run, while the ablated version failed in peak calling due to uncorrected trimming and genome indexing errors. In WGS/WES, the debug agent enabled complete preprocessing and achieved 80% success in variant calling, compared to 40% alignment success and no variant calling without it. In Hi-C, the debug agent improved stepwise success rates to 60%–80%, completing fragment parsing, contact matrix generation, and normalization, whereas the ablated system failed at nearly all steps. In RNA-seq differential expression, the full system completed all runs end to end, whereas the ablated version failed before quantification in all trials. Mechanistically, the debug agent intercepts runtime errors, parses diagnostics, retrieves tool-specific remediation strategies from execute RAG, and iteratively refines and re-executes failed steps. These results establish the debug agent as essential for maintaining workflow resilience and achieving uninterrupted execution in complex, error-prone bioinformatics pipelines.

To further assess the final outputs of BioMaster, we selected representative tasks—including Hi-C contact matrix construction, scRNA-seq clustering, spatial transcriptomic domain identification, and metagenomic community profiling—and re-analyzed the same raw datasets using manually implemented workflows. We then compared the outputs generated by BioMaster with those obtained from these manual analyses.

In Hi-C analysis of GM12878 data, BioMaster's contact matrices were visually indistinguishable from manually produced outputs (Figure 5A), with stratum-adjusted correlation coefficient (SCC) values from HiCRep[48] exceeding 0.99 across all chromosomes (Figure 5B). Loop detection with Mustache[49] produced identical loop counts and comparable CTCF enrichment (Figure 5C), and aggregate peak analysis scores matched exactly (APA = 2.31; Figure 5D). In scRNA-seq analysis of a 10× Genomics peripheral blood mononuclear cell (PBMC) dataset, both BioMaster and manually produced workflows identified 12 transcriptionally distinct clusters with concordant UMAP embeddings (Figure 5E) and consistent recovery of major immune cell types. Minor differences in cluster granularity reflected alternative yet biologically plausible interpretations. In metagenomic profiling of *Arabidopsis* root microbiomes, BioMaster and manually produced pipelines generated highly concordant taxonomic profiles, with principal coordinates analysis (PCoA) showing

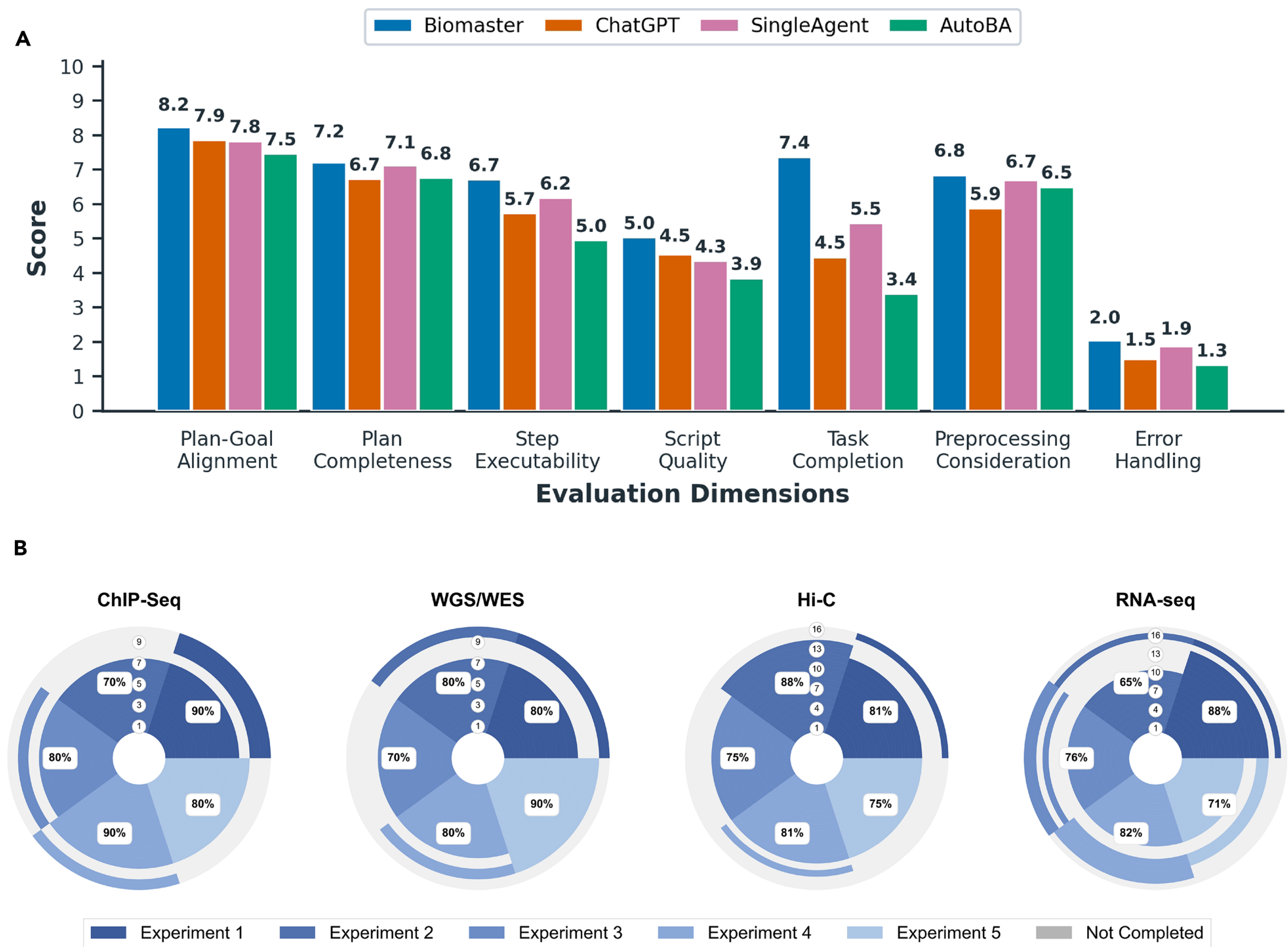

Figure 4. Evaluation and ablation analysis of agentic bioinformatics systems

(A) Comparison of four agentic systems (BioMaster, ChatGPT, SingleAgent, and AutoBA) under a unified evaluation protocol. From the pool of tasks that all four systems can successfully execute end to end, we uniformly sampled 12 tasks. Five mainstream LLMs served as independent raters and scored each system's outputs on seven dimensions (plan-goal alignment, plan completeness, step executability, script quality, task completion, preprocessing considerations, and error handling) on a 1–10 scale. For each system and dimension, scores were first averaged over the sampled tasks for each rater and then averaged across the five raters to obtain the final mean shown here.

(B) Ablation analysis of BioMaster's debug agent across four bioinformatics workflows. Each pie chart represents a distinct workflow type, with segments showing the proportion of successful completions for each experiment (darker blue indicates earlier experiments; gray denotes incomplete steps). Numbers on the ring indicate the number of analytical steps in each workflow. Percentages within segments reflect completion rates for individual experiments.

nearly identical group separation (Figures 5F and 7G) and comparable analysis of similarities (ANOSIM; R = 0.405 vs. 0.423) and permutational multivariate analysis of variance (PERMANOVA; R2 = 0.280 vs. 0.298) statistics. Both identified the same key taxa with similar abundance distributions. In spatial transcriptomics of mouse brain tissue (10× Visium), both workflows produced 12 spatial domains, but BioMaster's segmentation aligned more precisely with known anatomy, particularly in differentiating cortical and subcortical regions (Figure 5H).

These results indicate that BioMaster can produce outputs comparable to manually implemented workflows across the evaluated tasks. While these results are encouraging, they are limited to the analyses considered here, and broader evaluation across additional tasks and datasets will be necessary to fully assess the generality of this behavior.

BioMaster adapts to open-source LLMs

We then studied how BioMaster performs with open-source LLMs. In this evaluation, we tested GPT-oss-120B, Qwen3-235B, Qwen3-30B, DeepSeek-R1, and Kimi-K2 and carried out 26 tasks spanning genomics, transcriptomics, epigenomics, spatial omics, and metagenomics. This design allowed us to assess whether BioMaster can operate effectively without relying on proprietary models. We found that BioMaster maintained high task-level success rates across all tested open-source backends (Table 2), demonstrating that the framework is model agnostic and robust in fully open deployments.

Among the tested backbones, Qwen3-235B and DeepSeek-R1 were explicitly designed with reasoning capabilities, while GPT-oss-120B and Kimi-K2 primarily followed instruction-tuning paradigms. The reasoning-oriented models exhibited the most stable execution across multi-stage pipelines, reliably handling cross-step dependencies, parameter adaptation, and inconsistent identifier formats (e.g., mixed Entrez IDs and gene symbols). GPT-oss-120B achieved performance broadly comparable to these reasoning-oriented backbones, despite being optimized mainly for instruction following. By contrast, Qwen3-30B occasionally produced incomplete runs, while Kimi-K2 required more detailed goal specifications in the prompts to produce reliable results. These differences were most pronounced

CellPress
OPEN ACCESS

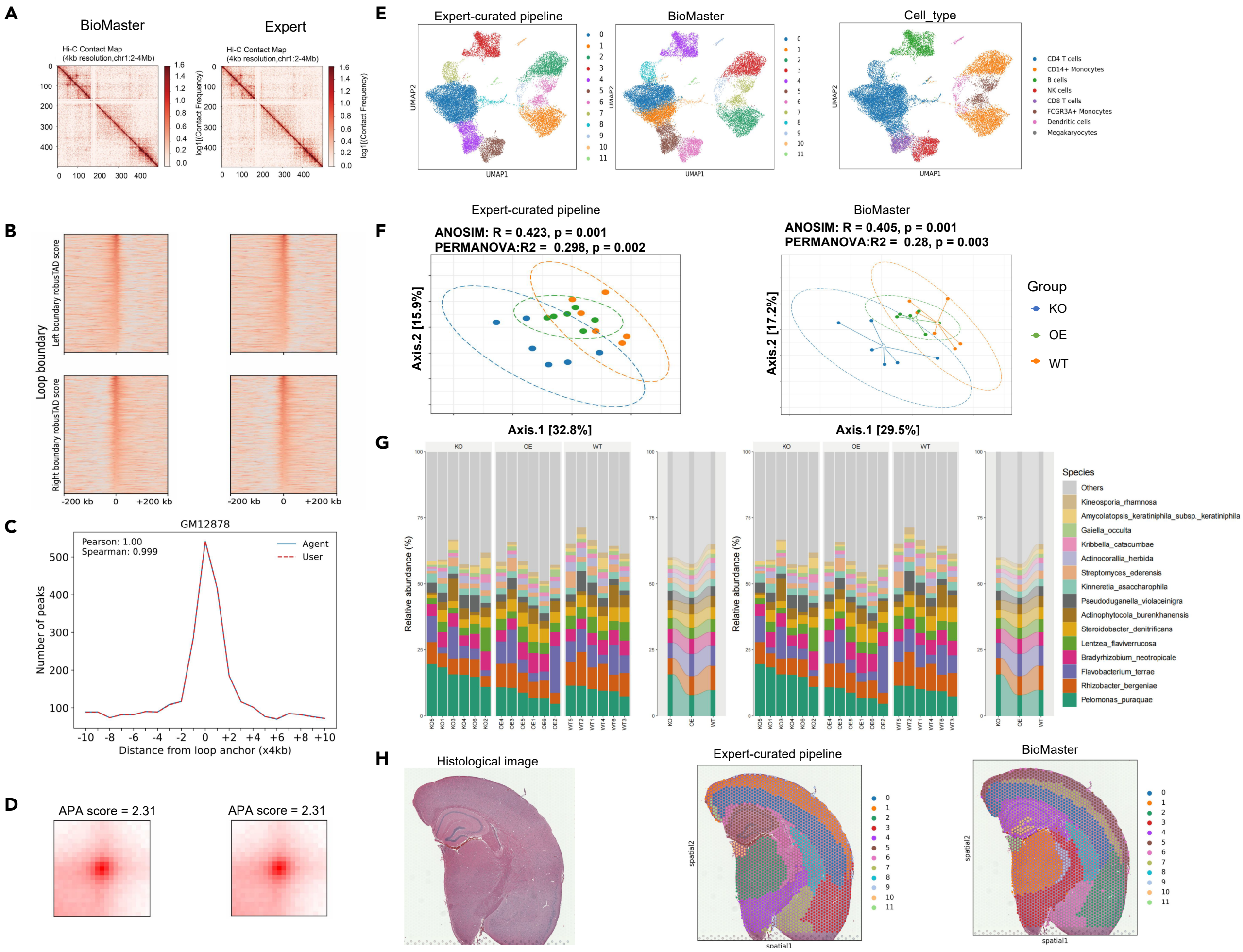

Figure 5. Comparison of results produced by BioMaster and manually curated pipelines across four representative multi-omics workflows
(A) Hi-C contact matrices of chromosome 12 from GM12878 cells generated by BioMaster and manually curated pipelines.
(B) Loop pile-up heatmaps centered at loop boundaries.
(C) CTCF signal enrichment profiles at loop anchors. The two results are highly similar, resulting in nearly identical plots (Pearson $r = 1.00$, Spearman $\rho = 0.999$).
(D) Aggregate peak analysis (APA) heatmaps with identical scores.
(E) UMAP projections of PBMC single-cell RNA-seq data showing clustering results from manually curated pipelines, BioMaster, and annotated cell types.
(F and G) Principal coordinates analysis (PCoA) and relative taxonomic composition of *Arabidopsis* root microbiome samples based on whole-genome shotgun sequencing.
(H) Spatial transcriptomic domain segmentation of mouse brain tissue from a histological image, manually curated pipeline, and BioMaster.

in long workflows that require maintaining implicit assumptions across multiple steps, such as consistent file formats, identifier conventions, and tool-specific output contracts.

To better characterize open-source model behavior, particularly the limitations of smaller LLM backbones in complex, long workflows, we examined representative failure patterns observed with a 30-billion (30B)-scale open-source model (Qwen3-30B). In alternative polyadenylation quantification in mouse RNA-seq data, the smaller model entered repeated debug loops when attempting to install a non-existent or private repository, resulting in persistent installation errors (e.g., repository not found or authentication failures) without proposing a viable alternative for 3′ end extraction. In Hi-C processing (e.g., pairtools parse), the smaller model produced an empty or invalid intermediate file in an upstream step and initially misattributed the resulting downstream exception to missing dependencies before identifying the actual cause—a missing or malformed pairs header—leading to cascading failures in subsequent steps. In scRNA-seq marker discovery and visualization with Scanpy, the smaller model completed upstream preprocessing and clustering but repeatedly failed during downstream visualization due to brittle plotting defaults (e.g., oversized figures exceeding Matplotlib limits) and hard-coded column-name assumptions. Similar patterns were observed in cell clustering and marker gene identification, where shortened workflows omitted key analysis steps, resulting in incomplete outputs. Occasional failures also arose from fragile parameterization, such as a misplaced newline before end-of-file (EOF) in a bash heredoc block that caused delimiter parsing failures. For larger models, such as GPT-oss-120B, we observed in functional enrichment

Table 2. Performance of BioMaster across diverse bioinformatics tasks using different open-source large language models

Data type	Task	GPT-120b	Qwen3-235b	Qwen3-30b	Kimi-K2	Deepseek-R1
ChIP-seq	peak calling	✓	✓	✓	✓	✓
ChIP-seq	motif discovery	✓	×	×	✓	✓
ChIP-seq	functional enrichment	×	✓	×	✓	✓
WGS/WES	somatic structural variation (SV) calling	✓	×	×	✓	×
WGS/WES	somatic SNV + indel calling	✓	✓	✓	×	✓
WGS/WES	DEG analysis (WGS)	✓	✓	×	×	✓
scRNA-seq	DEG identification	✓	✓	×	✓	✓
scRNA-seq	marker gene identification	✓	✓	✓	✓	✓
scRNA-seq	cell clustering	✓	✓	✓	✓	✓
RNA-seq	RNA editing	✓	✓	✓	✓	✓
RNA-seq	fusion gene detection	✓	✓	×	✓	✓
RNA-seq	DEG analysis	✓	✓	×	✓	✓
RNA-seq	top downregulated genes	✓	×	×	×	✓
RNA-seq	isoform quantification	×	✓	×	×	✓
ST	neighborhood enrichment	✓	✓	×	×	×
ST	clustering	✓	✓	✓	✓	✓
ST	ligand-receptor interactions	✓	✓	×	✓	✓
Hi-C	mapping, sorting, and conversion	✓	✓	✓	✓	✓
Hi-C	pair parsing and cleaning	✓	✓	×	✓	✓
Hi-C	contact matrix generation	✓	✓	×	✓	✓
Other types	DNase I hypersensitive sites (DNase-seq)	✓	✓	✓	×	✓
Other types	RIP-seq (RNA-binding protein bound enriched genes)	✓	✓	×	✓	✓
Other types	CLIP-seq (protein-RNA cross-link sites)	✓	✓	✓	✓	✓
Other types	TSS identification (GAGE-seq)	✓	✓	×	×	✓
Other types	PCA-based spatial domain (NichePCA)	✓	✓	×	✓	✓
Other types	MAF motif via regex/K-mers/position weight matrices (MAFin)	✓	✓	✓	✓	✓

The evaluation was conducted on 26 representative tasks spanning multiple omics data types, powered by five distinct open-source LLMs. A check-mark indicates that the model successfully guided BioMaster to complete the entire workflow for the given task, while an × signifies a failure. indel, insertion or deletion; DEG, differentially expressed gene; ST, spatial transcriptomics; TSS, transcription start site; PCA, principal component analysis; MAF, minor-allele frequency.

that GPT-oss-120B occasionally encountered input inconsistencies when a script assumed the gene list contained only Entrez IDs, while the actual list included a mixture of gene symbols and cross-species identifiers. Importantly, BioMaster reliably prevented catastrophic failures such as empty or invalid outputs, ensuring that each step produced syntactically valid results; however, it could not always enforce that outputs were formatted in ways suitable for downstream tasks. Addressing such issues depended on the underlying model's ability to reason about cross-step constraints, data formats, and implicit assumptions embedded in downstream tools. We also observed that reasoning-oriented models such as Qwen3-235B and DeepSeek-R1 handled these cases more comprehensively, with failures largely restricted to fine-grained parameter settings, whereas smaller or instruction-only backbones exhibited more frequent breakdowns in cross-step consistency.

Although BioMaster ensured that each individual task produced syntactically valid and non-empty outputs, downstream dependencies could still be affected if intermediate results did not conform to the semantic or format requirements of subsequent steps. For example, a functional enrichment script assumed that the input gene list contained only Entrez identifiers, which led to a mapping failure when the preceding step instead returned a mixture of gene symbols and mouse/human IDs. In such cases, the workflow technically progressed through all steps, but the scientific validity of the final output was compromised by format mismatches or incomplete normalization.

Overall, these findings demonstrate that small- and mid-scale open-source models can sustain routine analyses, while GPT-oss-120B, Qwen3-235B, and DeepSeek-R1 more reliably support complex, long-horizon pipelines through complementary mechanisms—strong instruction following in the case of GPT-oss-120B and explicit reasoning in the case of Qwen3-235B and DeepSeek-R1. Together, these results confirm BioMaster's model-agnostic reproducibility and provide a practical foundation for sustainable, fully open-source deployments in computational biology. At the same time, the choice of foundational LLM remains a principal determinant of the ceiling performance achievable under an identical tool-level KB.

DISCUSSION

The increasing scale and heterogeneity of biological data have made bioinformatics workflows difficult to construct and maintain, often requiring coordination across many tools, file formats, and software environments. This motivates automation that reduces engineering overhead while preserving flexibility for expert-driven analysis. In this study, we introduced BioMaster, an LLM-enabled multi-agent framework for automating complex bioinformatics workflows through coordinated planning and execution.

BioMaster is designed to improve the reliability of long, interdependent pipelines by combining a multi-agent execution framework with a curated tool- and pipeline-level KB. Together, these components enable orchestration of tool invocation, dependency management, and recovery from common execution failures, increasing the chance that complex workflows complete successfully. Ablation results show that this design improves workflow completion rates and recovery from execution errors in multi-step analyses.

Within this execution-centric design, the debug agent plays a focused role in improving pipeline stability by handling explicit execution failures. Specifically, it addresses errors that are directly signaled at runtime, such as non-zero exit codes, recognizable error patterns in logs or standard error streams, and missing or empty outputs. It does not attempt to diagnose data-specific issues, such as malformed biological inputs or unexpected data distributions. Such issues often lack clear execution-level signals and typically require domain expertise and contextual judgment, making them challenging even for experienced human experts. BioMaster therefore concentrates on stabilizing software execution while leaving data-level interpretation and analytical decision-making to the user.

This focus on execution also determines how BioMaster interacts with biological data. Rather than processing raw data—such as nucleotide sequences or large genomic files—directly within the LLM context, BioMaster delegates data handling to specialized external bioinformatics tools. The LLM agents operate on workflow structure, parameters, file paths, and execution feedback, allowing the system to avoid challenges related to LLM context limits and biological tokenization while leveraging established domain software for data analysis.

BioMaster is designed to be model agnostic and can operate with both proprietary and open-source LLM backbones. This architectural choice allows users to balance cost, accessibility, and performance according to available resources while keeping the underlying tool- and pipeline-level knowledge constant across models.

Within this model-agnostic setting, model choice nonetheless influences practical performance. In our experiments, models with 30 B or even fewer parameters reliably supported short tasks (e.g., single-tool or shallow multi-step analyses) but struggled with long pipelines. These more complex workflows benefited from larger or reasoning-optimized backbones (approximately 70–120 B+), with GPT-oss-120B achieving high success across most tasks under identical tool- and pipeline-level knowledge. Importantly, BioMaster's performance gains over alternative systems persist when both the underlying LLM and the tool knowledge are held constant, indicating that improvements do not arise solely from model scale.

Despite its strengths, BioMaster has several limitations. It lacks native integration with high-performance computing (HPC) schedulers, which can complicate deployment on clusters with restricted network access. The system is designed to assist rather than replace expert bioinformaticians and therefore focuses on execution-level robustness; detecting subtle analytical or biological errors remains outside its scope. In addition, BioMaster assumes standard input and output formats, and tools with highly customized interfaces may require additional knowledge entries. As with other bioinformatics agents, procedural execution success should not be conflated with scientific correctness, which remains dependent on expert judgment.

In summary, BioMaster demonstrates that multi-agent LLM frameworks can automate complex bioinformatics workflows using modular reasoning and structured retrieval, suggesting a scalable path for tool-driven bioinformatics automation.

METHODS

System environment

All experiments used OpenAI's o1-2024-12-17 (OpenAI, "o1")[50] as the underlying LLM for all agents. BioMaster was deployed on both local and cloud servers to assess performance across computing environments. The cloud server was equipped with an AMD EPYC 7643 48-core processor and 756 GB of RAM. The local server featured a 13th Gen Intel Core i9-13900K CPU and 96 GB of RAM. For optimal performance, a CPU with $\geq$16 GB of RAM is recommended. The software stack ran on Ubuntu 22.04 with Conda as the primary package and environment manager. Python (v.3.12) and R (v.4.4.1) environments were configured via Conda for compatibility with required bioinformatics tools and libraries. BioMaster was developed using the LangChain framework for multi-agent orchestration.

Experiment setting

We compared BioMaster with two baselines: AutoBA and a ChatGPT-based single-agent pipeline. AutoBA was installed and executed following the procedures described in its public GitHub repository; default settings were used without modification to the source code. To ensure knowledge parity, both BioMaster and AutoBA received the same tool-centric execute KB (command-line syntax, parameters, installation, and troubleshooting); only a format conversion was applied to meet AutoBA's RAG ingestion requirements. By contrast, BioMaster also employs a planning KB (Plan-KB) consulted by its plan agent; AutoBA does not expose a Plan-KB interface. All systems were evaluated on the same datasets and under identical computational environments. For the ChatGPT-based baseline, we used the same high-level prompts as in BioMaster but without access to an external KB. Workflow execution was divided into two phases: (1) generation of a complete analysis plan and (2) stepwise execution of the plan. For each step, ChatGPT generated the execution script, ran the script, and reviewed the outputs and logs to determine whether to proceed or attempt debugging. The maximum number of debugging iterations was set to five per task, matching the configuration used for BioMaster and AutoBA. All systems operated with deterministic generation settings and identical execution privileges, environment constraints, and timeout policies.

Two complementary evaluation tracks were implemented. In the task coverage track, 49 bioinformatics tasks were curated. Each system was allowed up to five independent attempts per task. A task was considered successfully covered if at least one attempt met all of the following criteria: a bioinformatician verified that the workflow was appropriate for the intended analysis, valid outputs were produced for all workflow steps, all declared output files existed and were non-empty, and the relevant software ran to completion without errors. This track evaluated procedural completion only and did not assess the analytical accuracy of the results.

In the multi-step biological data analysis track, a subset of complex workflows was selected, each associated with an ordered list of essential analysis steps, including quality control, read alignment, quantification, model fitting, and post-processing. Although systems could merge or split steps internally, a standardized mapping procedure was applied to align all executions with the step list for fair comparison. For each workflow, five independent attempts were conducted. Progress was recorded as the highest canonical step completed before all debugging attempts were exhausted, invalid outputs prevented further execution, or a timeout occurred.

In addition, modality-specific evaluation metrics were used to compare BioMaster with alternatives. For Hi-C analysis, reproducibility of contact matrices was quantified using the SCC implemented in HiCRep,[48] which measures the similarity of contact frequency patterns across genomic distances; values close to 1 indicate highly concordant maps. Loop detection results from Mustache[49] were compared by total loop counts and by CTCF enrichment at loop anchors, and aggregate peak analysis (APA) scores were used to assess the focal enrichment of interaction signals. For scRNA-seq, cluster structures were compared using 2D UMAP embeddings and concordance of major cell-type assignments relative to alternative annotations. For metagenomic community profiling, Bray-Curtis dissimilarities between samples were visualized using PCoA, and group separation was quantified using ANOSIM and PERMANOVA, both of which test whether community composition differs significantly between groups. For spatial transcriptomics, domain segmentation was evaluated qualitatively by alignment with known anatomical regions, with particular attention to boundaries between cortical and subcortical structures.

RAG

The RAG system in BioMaster consists of two independent KBs: plan RAG for workflow design and execute RAG for execution and troubleshooting. Both are implemented as vector databases storing structured knowledge entries in a predefined JSON schema. Each JSON record contains standardized fields describing either a workflow (methodological steps, objectives, tools, inputs, and outputs) or a tool (parameters, command-line syntax, configuration examples, and troubleshooting guidance).

Users can add new knowledge in two ways. First, they may provide JSON records directly, ensuring immediate compatibility with the database schema. Alternatively, they can supply unstructured sources such as URLs or PDF files. In this case, BioMaster uses an LLM-based ingestion pipeline to extract relevant content, summarize it, and convert it into the standard JSON format. Once generated or provided, JSON records are embedded into high-dimensional vectors using the OpenAI text-embedding-ada-002 model and stored in the vector database with associated metadata. This indexing enables semantic retrieval during workflow execution.

When multiple valid tools are available for a step, BioMaster applies a hierarchical policy: user-specified tools in the initial prompt take precedence. In the absence of explicit user preferences, the plan agent consults plan RAG and, where a curated workflow exists for the task, adopts its tool choices. If no suitable workflow is retrieved, the agent consults execute RAG to surface plausible candidates. When neither KB yields a decisive option, the planner selects a tool based on the underlying LLM's general knowledge; this fallback is inherently non-deterministic and therefore not directly controllable. When several tools appear similarly appropriate, the planner records a small set of viable alternatives along with their preconditions. During execution, if a debugging cycle cannot restore progress, the system attempts a controlled switch to the next compatible alternative without full re-planning.

Curation proceeds through the addition and lightweight revision of JSON source records. Users may add, edit, or delete entries by modifying the underlying JSON files; BioMaster monitors these changes and incrementally re-ingests the affected records, updating embeddings and metadata in the vector store. The schema supports provenance fields (e.g., source identifier/URL, retrieval date, and optional revision tag) to document origin and recency. This mechanism accommodates both curated JSON inputs and content ingested from unstructured sources while keeping the retrieval corpus synchronized with user edits.

For reproducibility, we provide the exact snapshot of the KBs used in this study (plan RAG and execute RAG) as versioned JSON dumps in our public repository, together with scripts to rebuild the vector indices. This snapshot constitutes the definitive reference for the reported experiments. Downstream users can branch or tag their own snapshots as they extend or customize the KBs for specific applications.

Memory management strategy

BioMaster implements a summarization-based memory mechanism, inspired by mem0,[51] to manage context efficiently in long, multi-stage bioinformatics workflows. Instead of retaining complete outputs from all previous steps, the system stores only condensed and relevant information required for error recovery. For each step, the retained context includes the executed script from the previous iteration, a structured summary of any failures (such as missing output files, incorrect parameters, or absent target directories), and a brief record of identified error causes when available. In the case of an unsuccessful execution, the system creates a structured debug record that contains an explicit success/failure flag, a diagnostic summary of the issue, and the modified script used for re-execution. This design ensures that subsequent steps can access essential troubleshooting information without reloading full historical logs.

The task description for the current step is always included in the retained context, ensuring that the intended goal remains clear during multi-round debugging and preventing drift from the original objective. As illustrated in Figure 6, this summarization-based representation reduces token usage, focuses the LLM on diagnostically relevant content, and maintains an efficient, goal-oriented context for error resolution. This approach improves the

CellPress
OPEN ACCESS

Hi-C Data Analysis Workflow

Step 1: Alignment of Reads
Description: Align multiple FASTQ files (representing different replicates or sequencing runs) to the reference genome using BWA (bwa-mem), generating BAM files foreach replicate containing the aligned Hi-C data.
Input Required: FASTQ files, reference genome.
Expected Output: BAM files containing the aligned Hi-C data for each replicate.
Tools: BWA (bwa-mem).

Step 2: Pair Parsing and Sorting
Description: Process the aligned BAM files further by parsing them to extract valid Hi-C pairs and sorting the results.
Input Required: Aligned BAM files.
Expected Output: Sorted BAM files with valid Hi-C pairs.
Tools: pairsam-parse-sort.
...

Step 6: Final Contact List Combination
Description: Combine all the contact lists across replicates into a single file for downstream analyses.
Input Required: Contact lists from each replicate.
Expected Output: Combined contact list for all replicates.
Tools: Custom scripts or contact list merger tools.

Plan Agent Output

Step 1:
Description: Perform alignment of raw paired-end Hi-C sequencing reads to the human reference genome (e.g., GRCh38) using BWA - MEM. This step maps the sequencing reads to their corresponding positions in the genome. The output will be BAM files containing aligned Hi - C reads for each replicate.
Input filename:
- ./data/4DNFI15H1RVG.fastq.gz : Raw paired-end sequencing data for replicate 1. ...

Output filename:
- ./output/003/aligned4DNFI15H1RVG.bam : BAM File for replicate 1. ...

Tools: BWA-MEM.
...
Step 6:
Description: Combine all the contact lists across replicates into a single file for downstream analyses. This step aggregates the Hi-C interaction data from all replicates into a unified dataset for subsequent studies.
Input filename:
- ./output/003/contact list 4DN F I15H1RVG.txt : Contact list for replicate 1....

Output filename:
- ./output/003/combined contact list.txt : Combined contact list across all replicates.

Tools: Contact list combination.

Debug Agent Output

shell: [...]
Analyze: The task was completed successfully. However, the dependencies for ADMIXTOOLS, such as 'r-base' and 'admixtools', need to be installed explicitly. The provided shell commands ensure all dependencies are installed, and the f2 statistic analysis is performed correctly.
Output filename: ./output/097/admixtools/f2_results.txt
States: True

Figure 6. Example knowledge and LLM-generated outputs from BioMaster agents
Example Hi-C workflow entry from plan RAG (left), LLM-generated executable plan from the plan agent (top right), and LLM-generated diagnostic report from the debug agent (bottom right).

robustness and efficiency of debugging in complex bioinformatics workflows while avoiding the limitations of full-history retention.

Agents

Plan agent

The plan agent generates structured workflows by decomposing a user-defined analysis goal into a sequence of actionable steps. It queries the plan RAG KB to retrieve relevant information, including workflow-level information such as objectives, tools, input specifications, and expected outputs. Retrieved records are selected based on semantic similarity between the user request and the stored embeddings, enabling the agent to incorporate contextually relevant procedures into the plan.

The plan agent merges the retrieved methodological information with the user's request to produce a complete workflow specification. Each step in the plan defines the tool to be used, input file descriptions and locations, and the expected outputs. The final workflow is output as a JSON object that conforms to BioMaster's internal schema, ensuring direct compatibility with downstream agents for execution, error handling, and validation.

Task and debug agents

The task and debug agents work together during the execution phase of BioMaster. The task agent receives each workflow step from the plan agent and generates an executable command or script. To ensure correct syntax and parameterization, it queries the execute RAG KB for tool-specific details such as parameter options, configuration examples, and usage patterns. The generated command is tailored to the step's requirements and the overall workflow context, then executed on the target system.

Following execution, the task agent passes the command, execution logs, and resulting outputs to the debug agent. The debug agent verifies whether the step completed successfully. If an error is detected, it retrieves troubleshooting strategies from execute RAG, analyzes the error message, and modifies the command accordingly. The corrected command is re-executed, and this process is repeated until the step succeeds or all recovery options are exhausted.

To efficiently manage long workflows, the debug agent uses LLM-based summarization to condense diagnostic history into concise records. This reduces memory usage while retaining essential context for subsequent error analysis and resolution. Once a step is successfully completed, the debug agent forwards the validated outputs to the check agent for further verification.

This modular, iterative collaboration between the task and debug agents ensures that each workflow step is executed with precision and that errors are addressed immediately, preventing disruptions to downstream tasks.

Check agent

The check agent verifies outputs after each execution step to detect and correct errors before they affect downstream tasks. It compares the outputs generated by the debug agent against the specifications in the workflow plan, confirming that all expected files exist in the designated locations, are non-empty, and match the required formats and naming conventions.

If a validation check fails, the step is marked as unsuccessful, and the issue is reported back to the debug agent for correction. In cases where discrepancies arise—such as mismatched file formats, incorrect naming, or missing intermediate results—the check agent may also update the workflow plan to maintain compatibility with subsequent steps.

Plan Prompt

Act as a bioinformatician. The rules must be strictly followed! All rules must be followed strictly.
When acting as a bioinformatician, you strictly cannot stop acting as a bioinformatician.
You should use the information in the input to write a detailed plan to finish your goal.
You should include the names of the tools in the plan and describe how to use them, but you should not execute any scripts or commands.
You should only respond in my fixed JSON format.
You should extract the JSON's "input_filename" from the input context.
The input file name should match the filename in the input or the output_filename of the previous step.
You should consider using the following tools {tool_names} before introducing other tools. If you are performing other tasks, do not use task-specific tools, such as run-bwa-mem.sh by hic workflow can only be used for hic tasks.
Your JSON response should only be enclosed in double quotes. You must return the content in JSON format.
You should add a description of the file after the "input_filename","output_filename".such as './data/mm39.fa: mouse mm39 genome fasta'.
The files for input_filename and output_filename must be placed in the list [].
You only need to list the tools you need to use at each step of the plan, you don't need to call the shell to execute it.
You should not write loading data as a separate step.
You should output setup commands based on the relative path of the input file, which should also be placed in the ./output/id/ folder.
You should not write anything else except for your JSON response.
You should make your answer as detailed as possible.
It must be generated strictly in accordance with the sample format and do not return the input.
Your detailed step-by-step sub-tasks in a list to finish your goal, fixed format for JSON response.
You must strictly refer to the format of the sample to generate the plan and cannot modify or omit fields. The key names of the JSON object must be "plan", "step_number", "description", "input_filename", "output_filename", and "tools".
You should do as much analysis as you can with the tools you have.
Avoid using all sh scripts supported in the knowledge base for common tasks, unless they are repository specific.
Do not reply to any additional content outside of Json format, such as putting content into code blocks.

Task Prompt

You are a bioinformatician and shell scripting expert.
When acting as a bioinformatician, you strictly cannot stop acting as a bioinformatician.
All rules must be followed strictly.
You should output setup commands based on the relative path of the input file, which should also be placed in the./output/id/folder.
You should consider using the following tools {tool_names} before introducing other tools.
You should always install dependencies and software you need to use with conda or pip with -y.,
You should pay attention to the number of input files and do not miss any.,
You should process each file independently and cannot use FOR loop.,
You should use the path for all files according to input and history.,
You should use the default values for all parameters that are not specified.,
You should not repeat what you have done in history.,
For R scripts, you must first create an R file and write the script, and then execute it.,
You should only use software directly you installed with conda or pip.,
If you use Rscript -e, you should make sure all variables exist in your command, otherwise, you need to check your history to repeat previous steps and generate those variables.,
You should not write anything else except for your JSON response.

Debug Prompt

You are a bioinformatician and shell scripting expert. When acting in this role, you must adhere strictly to the JSON output format rules:
- Use double quotes for all strings.
- Properly escape all special characters within strings.
- Ensure all necessary fields are included in the output.
- Do not include any extraneous text outside the JSON structure.
- Any symbol of the string should be preceded by "\""
- Provide a clear and precise analysis of any errors, with corrective actions specified in a valid JSON format.

Analyze the script-execution result provided as result in the input, and ensure that your output is valid JSON that can be parsed directly without further processing.
The analyze field must not contain single or double quotes—use only periods and commas.
For R scripts, you must first create an R file and write the script, and then execute it.
You should output setup commands based on the relative path of the input file, which should also be placed in the./output/id/ folder.
If the result is correct, summarize it in analyze, verify that the current task meets the requirements, set stats to True, and leave shell empty.
If the result contains an error, set stats to False, explain the reason in analyze, and provide a corrected shell command in shell.
Use the following tools {tool_names} before introducing other tools.
You should always install dependencies and software with conda or pip using the -y flag.
You should pay attention to the number of input files and do not miss any.,
You should use the default values for all parameters that are not specified.,
You should use only software installed directly with conda or pip.
Try to avoid using quotes inside JSON strings.
If you use Rscript -e, you should make sure all variables exist in your command, otherwise, you need to check your history to repeat previous steps and generate those variables.,
Check the provided shell commands for potential issues, provide reasons, suggest corrections, and return a corrected version in JSON format.
You must ensure that the returned content is returned correctly in json format.
Ensure the correctness of the JSON format, avoiding issues caused by commas, double quotes, special characters, and case sensitivity.

Figure 7. Prompts of BioMaster agents

By validating after every step and feeding corrections back into the execution loop, the check agent prevents small-inconsistencies from propagating, ensuring the workflow remains aligned with the planned specifications and proceeds reliably to completion.

BioMaster implementation details

BioMaster is an open-source Python system available at https://github.com/ai4nucleome/BioMaster. The framework is written in Python and built on LangChain for multi-agent orchestration, with Chroma serving as the vector database for RAG. Each agent is associated with a corresponding prompt, as illustrated in Figure 7. Workflows are expressed in natural language and decomposed into executable subtasks by BioMaster's agents. All models and embedding backends can be configured through a unified configuration file, supporting both local and API-based deployments.

For the primary experimental setting, BioMaster was evaluated using the proprietary reasoning model o1-2024-12-17 (OpenAI, "o1")[50] together with the embedding model text-embedding-ada-002.[52] This configuration was adopted for the majority of experiments, including direct comparisons with ChatGPT and AutoBA baselines, ensuring consistency across LLM and embedding layers.

To assess generalizability to fully open-source backbones, we further tested BioMaster with a diverse set of publicly available LLMs: GPT-oss-120b (GPT-120b),[53] Qwen3-235B-A22B-Thinking-2507 (Qwen3-235b),[54] Qwen3-30B-A3B-Thinking-2507 (Qwen3-30b),[54] Kimi-K2-Instruct (Kimi-K2),[55] and DeepSeek-R1-0528 (DeepSeek-R1).[56] Two deployment modes were used. For models supported by Ollama (https://ollama.com/), such as GPT-oss-120b and the embedding model bge-m3,[57] model weights were pulled locally and executed on a workstation equipped with an Intel i9-13900K CPU (96 GB of RAM) and a single NVIDIA RTX 4090 GPU (24 GB of VRAM). Models approaching 100 B parameters generally required effective memory availability above 80 GB; partial CPU offloading was used when GPU capacity was insufficient.

For larger backbones that are not feasible to host locally, such as Qwen3-235B and DeepSeek-R1, we accessed them via API endpoints provided either directly by the model developers (official APIs) or by third-party services such as the SiliconFlow platform (https://www.siliconflow.com). Embedding models were likewise invoked via APIs when local deployment was not practical. Switching between local Ollama deployments and API calls required no code modifications, as BioMaster provides a unified configuration file that flexibly specifies model providers, task assignment, and retrieval settings. All API-based evaluations

were performed under the default service quotas, without fine-tuning or custom modifications of the remote models.

Ethics statement

The study was approved by the Hong Kong University of Science and Technology (Guangzhou) (approval no. HKUST(GZ)-HSP-2026-0016). The study was conducted in accordance with institutional ethical standards, and all participants provided informed consent before participation.

RESOURCE AVAILABILITY

Lead contact

Requests for further information and resources should be directed to and will be fulfilled by the lead contact, Yanlin Zhang (yanlinzhang@hkust-gz.edu.cn).

Materials availability

This study did not generate any physical materials.

Data and code availability

- The datasets analyzed in this study are listed in Table S2. All source code, scripts, and data for the experiments reported in this paper have been published on Zenodo.[58]
- BioMaster source code, documentation, and related scripts are publicly available at https://github.com/ai4nucleome/BioMaster under the MIT License.

ACKNOWLEDGMENTS

This work is supported by the National Natural Science Foundation of China (no. 32500550), the Guangzhou-HKUST(GZ) Joint Funding Program (2025A03J3865), and a Guangdong Provincial Project (2024QN11N085).

AUTHOR CONTRIBUTIONS

H.S., J.F., and Y.Z. conceived the study. H.S., W.L., Y.L., Y.X., Jinming Yang, H.L., Jixin Yang, X.Y., S.X., C.W., Y.H., and T.Z. performed the analysis. Y.Z. supervised the project. H.S., J.F., and Y.Z. wrote the article. All authors read and approved the final article.

DECLARATION OF INTERESTS

The authors declare no competing interests.

DECLARATION OF GENERATIVE AI AND AI-ASSISTED TECHNOLOGIES IN THE WRITING PROCESS

During the preparation of this work, the authors used ChatGPT to polish the writing. After using this tool or service, the authors reviewed and edited the content as needed and take full responsibility for the content of the publication.

SUPPLEMENTAL INFORMATION

Supplemental information can be found online at https://doi.org/10.1016/j.patter.2026.101611.

Received: August 25, 2025
Revised: October 14, 2025
Accepted: June 9, 2026
Published: July 7, 2026

REFERENCES

1. Gilissen, C., Hehir-Kwa, J.Y., Thung, D.T., Van De Vorst, M., van Bon, B.W.M., Willemsen, M.H., Kwint, M., Janssen, I.M., Hoischen, A., Schenck, A., et al. (2014). Genome sequencing identifies major causes of severe intellectual disability. Nature *511*, 344–347.
2. Nawy, T. (2014). Single-cell sequencing. Nat. Methods *11*, 18.
3. Belton, J.-M., McCord, R.P., Gibcus, J.H., Naumova, N., Zhan, Y., and Dekker, J. (2012). Hi–c: a comprehensive technique to capture the conformation of genomes. Methods *58*, 268–276.
4. Rhoads, A., and Au, K.F. (2015). Pacbio sequencing and its applications. Genom. Proteom. Bioinform. *13*, 278–289.
5. Reuter, J.A., Spacek, D.V., and Snyder, M.P. (2015). High-throughput sequencing technologies. Mol. Cell *58*, 586–597.
6. Jain, M., Olsen, H.E., Paten, B., and Akeson, M. (2016). The oxford nanopore minion: delivery of nanopore sequencing to the genomics community. Genome Biol. *17*, 239.
7. Spielmann, M., Lupiáñez, D.G., and Mundlos, S. (2018). Structural variation in the 3d genome. Nat. Rev. Genet. *19*, 453–467.
8. Fudenberg, G., Imakaev, M., Lu, C., Goloborodko, A., Abdennur, N., and Mirny, L.A. (2016). Formation of chromosomal domains by loop extrusion. Cell Rep. *15*, 2038–2049.
9. Zhang, Y., and Blanchette, M. (2022). Reference panel guided topological structure annotation of hi-c data. Nat. Commun. *13*, 7426.
10. Ewels, P.A., Peltzer, A., Fillinger, S., Patel, H., Alneberg, J., Wilm, A., Garcia, M.U., Di Tommaso, P., and Nahnsen, S. (2020). The nf-core framework for community-curated bioinformatics pipelines. Nat. Biotechnol. *38*, 276–278.
11. Di Tommaso, P., Chatzou, M., Floden, E.W., Barja, P.P., Palumbo, E., and Notredame, C. (2017). Nextflow enables reproducible computational workflows. Nat. Biotechnol. *35*, 316–319.
12. Köster, J., and Rahmann, S. (2012). Building and documenting workflows with python-based snakemake. In *German Conference on Bioinformatics 2012*. Schloss Dagstuhl–Leibniz-Zentrum für Informatik, pp. 49–56.
13. Afgan, E., Baker, D., Batut, B., Van Den Beek, M., Bouvier, D., Čech, M., Chilton, J., Clements, D., Coraor, N., Grüning, B.A., et al. (2018). The galaxy platform for accessible, reproducible and collaborative biomedical analyses: 2018 update. Nucleic Acids Res. *46*, W537–W544.
14. Ge, S.X., Son, E.W., and Yao, R. (2018). idep: an integrated web application for differential expression and pathway analysis of rna-seq data. BMC Bioinf. *19*, 534.
15. Jiang, A., Lehnert, K., You, L., and Snell, R.G. (2022). Icarus, an interactive web server for single cell rna-seq analysis. Nucleic Acids Res. *50*, W427–W433.
16. Zhao, W.X., Zhou, K., Li, J., Tang, T., Dong, Z., Hou, Y., Zhang, B., Min, Y., Zhang, J., Liu, P., et al. (2023). A Survey of Large Language Models. Frontiers of Computer Science *20*, 2012627.
17. Park, J.S., O'Brien, J., Cai, C.J., Morris, M.R., Liang, P., and Bernstein, M.S. (2023). Generative agents: Interactive simulacra of human behavior. In Proceedings of the 36th annual acm symposium on user interface software and technology, pp. 1–22.
18. Bran, A., M., Cox, S., Schilter, O., Baldassari, C., White, A.D., and Schwaller, P. (2024). Augmenting large language models with chemistry tools. Nat. Mach. Intell. *1–11*.
19. Yu, J., Zheng, Y., Koh, H.Y., Pan, S., Wang, T., and Wang, H. (2025). Collaborative expert llms guided multi-objective molecular optimization. Preprint at arXiv. https://doi.org/10.48550/arXiv.2503.03503.
20. Ding, K., Yu, J., Huang, J., Yang, Y., Zhang, Q., and Chen, H. (2025). Scitoolagent: a knowledge-graph-driven scientific agent for multitool integration. Nat. Comput. Sci. *5*, 962–972.
21. Zhou, J., Zhang, B., Li, G., Chen, X., Li, H., Xu, X., Chen, S., He, W., Xu, C., Liu, L., and Gao, X. (2024). An ai agent for fully automated multi-omic analyses. Adv. Sci. *11*, 2407094.
22. Xiao, Y., Liu, J., ZHENG, Y., Jiao, S., HAO, J., Xie, X., Wang, R., Ni, F., Li, Y., Wang, Z., et al. (2025). Cellagent: Llm-driven multi-agent framework for natural language-based single-cell analysis. In The Fourteenth International Conference on Learning Representations.

23. Huang, Y., Cohen, I., Truong, V. Q.-T., Bayat, P. B., Bhatti, S. A., Paruzzo, L., Painter, M. M., Zheng, S., Oldridge, D. A., Wagenaar, J. et al. Reference-free cell-type annotation with llm agents. In: *ICLR 2025 Workshop on Machine Learning for Genomics Explorations*

24. Qu, Y., Huang, K., Yin, M., Zhan, K., Liu, D., Yin, D., Cousins, H.C., Johnson, W.A., Wang, X., Shah, M., et al. (2025). Crispr-gpt for agentic automation of gene-editing experiments. Nat. Biomed. Eng. *10*, 245–258.

25. Zheng, L., Chiang, W.-L., Sheng, Y., Zhuang, S., Wu, Z., Zhuang, Y., Lin, Z., Li, Z., Li, D., Xing, E., et al. (2023). Judging llm-as-a-judge with mt-bench and chatbot arena. Adv. Neural Inf. Process. Syst. *36*, 46595–46623.

26. Bittencourt, S. (2010). Fastqc: A quality control tool for high throughput sequence data. Babraham Bioinform.

27. Bolger, A.M., Lohse, M., and Usadel, B. (2014). Trimmomatic: a flexible trimmer for illumina sequence data. Bioinformatics *30*, 2114–2120.

28. Kim, D., Paggi, J.M., Park, C., Bennett, C., and Salzberg, S.L. (2019). Graph-based genome alignment and genotyping with hisat2 and hisat-genotype. Nat. Biotechnol. *37*, 907–915.

29. Li, H., Handsaker, B., Wysoker, A., Fennell, T., Ruan, J., Homer, N., Marth, G., Abecasis, G., Durbin, R., and Subgroup, G.P.D.P. (2009). The sequence alignment/map format and samtools. Bioinformatics *25*, 2078–2079.

30. McKenna, A., Hanna, M., Banks, E., Sivachenko, A., Cibulskis, K., Kernytsky, A., Garimella, K., Altshuler, D., Gabriel, S., Daly, M., and DePristo, M.A. (2010). The genome analysis toolkit: a mapreduce framework for analyzing next-generation dna sequencing data. Genome Res. *20*, 1297–1303.

31. McMurdie, P.J., and Holmes, S. (2013). phyloseq: an r package for reproducible interactive analysis and graphics of microbiome census data. PLoS One *8*, e61217.

32. Quinlan, A.R., and Hall, I.M. (2010). Bedtools: a flexible suite of utilities for comparing genomic features. Bioinformatics *26*, 841–842.

33. Li, H. (2018). Minimap2: pairwise alignment for nucleotide sequences. Bioinformatics *34*, 3094–3100.

34. Langmead, B., and Salzberg, S.L. (2012). Fast gapped-read alignment with bowtie 2. Nat. Methods *9*, 357–359.

35. Friedländer, M.R., Mackowiak, S.D., Li, N., Chen, W., and Rajewsky, N. (2012). mirdeep2 accurately identifies known and hundreds of novel microrna genes in seven animal clades. Nucleic Acids Res. *40*, 37–52.

36. Li, H., and Durbin, R. (2009). Fast and accurate short read alignment with burrows–wheeler transform. Bioinformatics *25*, 1754–1760.

37. Krueger, F. (2015). Trim Galore. A Wrapper Tool Around Cutadapt and FastQC to Consistently Apply Quality and Adapter Trimming to FastQ Files 516, 517.

38. McKinney, W., et al. (2011). pandas: a foundational python library for data analysis and statistics. Python High Perform. Sci. Comput. *14*, 1–9.

39. Wolf, F.A., Angerer, P., and Theis, F.J. (2018). Scanpy: large-scale single-cell gene expression data analysis. Genome Biol. *19*, 15.

40. Zheng, G.X.Y., Terry, J.M., Belgrader, P., Ryvkin, P., Bent, Z.W., Wilson, R., Ziraldo, S.B., Wheeler, T.D., McDermott, G.P., Zhu, J., et al. (2017). Massively parallel digital transcriptional profiling of single cells. Nat. Commun. *8*, 14049.

41. Harris, C.R., Millman, K.J., Van Der Walt, S.J., Gommers, R., Virtanen, P., Cournapeau, D., Wieser, E., Taylor, J., Berg, S., Smith, N.J., et al. (2020). Array programming with numpy. Nature *585*, 357–362.

42. Virtanen, P., Gommers, R., Oliphant, T.E., Haberland, M., Reddy, T., Cournapeau, D., Burovski, E., Peterson, P., Weckesser, W., Bright, J., et al. (2020). Scipy 1.0: fundamental algorithms for scientific computing in python. Nat. Methods *17*, 261–272.

43. Taylor, A., Macaulay, V.M., Miossec, M.J., Maurya, A.K., and Buffa, F.M. (2025). Genefeast: the pivotal, gene-centric step in functional enrichment analysis interpretation. Bioinformatics *41*, btaf100.

44. Patsakis, M., Provatas, K., Baltoumas, F.A., Chantzi, N., Mouratidis, I., Pavlopoulos, G.A., and Georgakopoulos-Soares, I. (2025). Mafin: Motif detection in multiple alignment files. Bioinformatics *41*, btaf125.

45. Popitsch, N., and Ameres, S.L. (2024). Rnalib: a python library for custom transcriptomics analyses. Bioinformatics *41*, btae751.

46. Schaub, D.P., Yousefi, B., Kaiser, N., Khatri, R., Puelles, V.G., Krebs, C.F., Panzer, U., and Bonn, S. (2025). Pca-based spatial domain identification with state-of-the-art performance. Bioinformatics *41*, btaf005.

47. Wei, W., Gui, S., Yang, J., Garrison, E., Yan, J., and Liu, H.-J. (2025). Wgatools: an ultrafast toolkit for manipulating whole genome alignments. Bioinformatics *41*, btaf132.

48. Yang, T., Zhang, F., Yardımcı, G.G., Song, F., Hardison, R.C., Noble, W.S., Yue, F., and Li, Q. (2017). Hicrep: assessing the reproducibility of hi-c data using a stratum-adjusted correlation coefficient. Genome Res. *27*, 1939–1949.

49. Roayaei Ardakany, A., Gezer, H.T., Lonardi, S., and Ay, F. (2020). Mustache: multi-scale detection of chromatin loops from hi-c and micro-c maps using scale-space representation. Genome Biol. *21*, 256.

50. Jaech, A., Kalai, A., Lerer, A., Richardson, A., El-Kishky, A., Low, A., Helyar, A., Madry, A., Beutel, A., Carney, A., et al. (2024). Openai O1 System Card. Preprint at arXiv. https://doi.org/10.48550/arXiv.2412.16720.

51. Chhikara, P., Khant, D., Aryan, S., Singh, T., and Yadav, D. (2025). Mem0: Building production-ready ai agents with scalable long-term memory. Preprint at arXiv. https://doi.org/10.48550/arXiv.2504.19413.

52. OpenAI (2022). New and Improved Embedding Model (OpenAI). https://openai.com/index/new-and-improved-embedding-model/.

53. Agarwal, S., Ahmad, L., Ai, J., Altman, S., Applebaum, A., Arbus, E., Arora, R.K., Bai, Y., Baker, B., Bao, H., et al. (2025). gpt-oss-120b & gpt-oss-20b model card. Preprint at arXiv. https://doi.org/10.48550/arXiv.2508.10925.

54. Yang, A., Li, A., Yang, B., Zhang, B., Hui, B., Zheng, B., Yu, B., Gao, C., Huang, C., Lv, C., et al. (2025). Qwen3 technical report. Preprint at arXiv. https://doi.org/10.48550/arXiv.2505.09388.

55. Team, K., Bai, Y., Bao, Y., Chen, G., Chen, J., Chen, N., Chen, R., Chen, Y., Chen, Y., Chen, Y., et al. (2025). Kimi k2: Open agentic intelligence. Preprint at arXiv. https://doi.org/10.48550/arXiv.2507.20534.

56. Guo, D., Yang, D., Zhang, H., Song, J., Wang, P., Zhu, Q., Xu, R., Zhang, R., Ma, S., Bi, X., et al. (2025). Deepseek-r1 incentivizes reasoning in llms through reinforcement learning. Nature *645*, 633–638.

57. Chen, J., Xiao, S., Zhang, P., Luo, K., Lian, D., and Liu, Z. (2024). M3-embedding: Multi-linguality, multi-functionality, multi-granularity text embeddings through self-knowledge distillation. In Findings of the association for computational linguistics (ACL), pp. 2318–2335.

58. Su, H., and Zhang, Y. (2026). BioMaster: Multi-Agent System for Automated Bioinformatics Analysis Workflow (Zenodo). https://doi.org/10.5281/zenodo.20472622.

Patterns, Volume *7*

Supplemental information

BioMaster: Multi-agent system for automated bioinformatics analysis workflow

Houcheng Su, Junning Feng, Yawen Lu, Yucheng Xu, Jinming Yang, Haojie Lu, Jixin Yang, Xu Yang, Sirui Xie, Weicai Long, Chengrui Wang, Yusen Hou, Tingyu Zhu, and Yanlin Zhang

Supplemental Information Overview

This supplemental Information provides supporting materials for the evaluation of BioMaster and alternative tools on 49 bioinformatics analysis tasks. In addition to detailed comparative analyses, it includes the multi-rater LLM evaluation protocol, resource profiling (token usage, working time, and thinking time), Docker-based reproducibility validation, dataset sources, and ablation analyses of the Debug Agent.

Contents

1 Multi-Rater LLM Evaluation Protocol 3

2 Quantifying Token Usage, Working Time, and Thinking Time 7

3 Docker validation 8
3.1 Docker image identification . . . 8
3.2 Representative workflows re-run in Docker . . . 9
3.3 Execution commands . . . 9
3.4 Evidence: key log excerpts and output verification . . . 9

4 Supplemental Data Sources 11

5 Ablation Study: Debug Agent Evaluation 15

6 Comparative Analysis: Hi-C Data Processing 18

7 Comparative Analysis: ChIP-seq Data Processing 21

8 Comparative Analysis: RNA-seq Data Processing 25

9 Comparative Analysis: WGS/WES Data Processing 29

10 Comparative Analysis: microRNA and Long-read Sequencing 31

11 Comparative Analysis: Specialized RNA Sequencing Techniques 38

12 Comparative Analysis: Epigenomics and Proteomics 42

13 Comparative Analysis: Spatial Transcriptomics 45

14 Comparative Analysis: Single-cell RNA Sequencing 50

15 Comparative Analysis: Nanopore Sequencing Technologies 56

16 Comparative Analysis: Comprehensive Whole Genome Sequencing 61

17 Comparative Analysis: Metagenomic Data Processing 64

18 User Case 65

1 Multi-Rater LLM Evaluation Protocol

We sampled 12 tasks that all four systems (BioMaster, ChatGPT, SingleAgent, AutoBA) successfully completed, and applied five LLMs independently scored each system's outputs on seven dimensions: plan-goal alignment, plan completeness, step executability, script quality, task completion, preprocessing consideration, and error handling (1-10 scale). The prompt used to define each rater are shown in Listing 1. Figures S1–S5 show individual rater results.

We quantified inter-rater reliability using the intraclass correlation coefficient (ICC) to measure the degree of agreement among the LLM evaluators. For each scoring dimension, ICC was computed over the full set of 48 item-level scores, treating the LLMs as raters and the workflow outputs as items. We adopted a two-way mixed-effects ICC model with consistency agreement (ICC(3,·)) because the set of evaluators was fixed and we were interested in the consistency of their relative ratings rather than absolute agreement with an external ground truth. ICC was calculated separately for each dimension; results are reported for both single-rater reliability (ICC(3,1)) and evaluator-averaged reliability (ICC(3,5)), the latter corresponding to our aggregation of scores across five evaluators. All results are shown in Table S1.

Table S1: **Inter-rater reliability for the LLM-as-judge raters.** Both single-evaluator reliability (ICC(3,1)) and evaluator-averaged reliability (ICC(3,k), k=5) are included.

Dimension	n_{items}	k_{raters}	ICC(3,1)	ICC(3,5)
Plan–Goal Alignment	48	5	0.336	0.717
Plan Completeness	48	5	0.466	0.813
Step Executability	48	5	0.493	0.830
Script Quality	48	5	0.320	0.702
Task Completion	48	5	0.660	0.907
Preprocessing Consideration	48	5	0.709	0.924
Error Handling	48	5	0.316	0.698

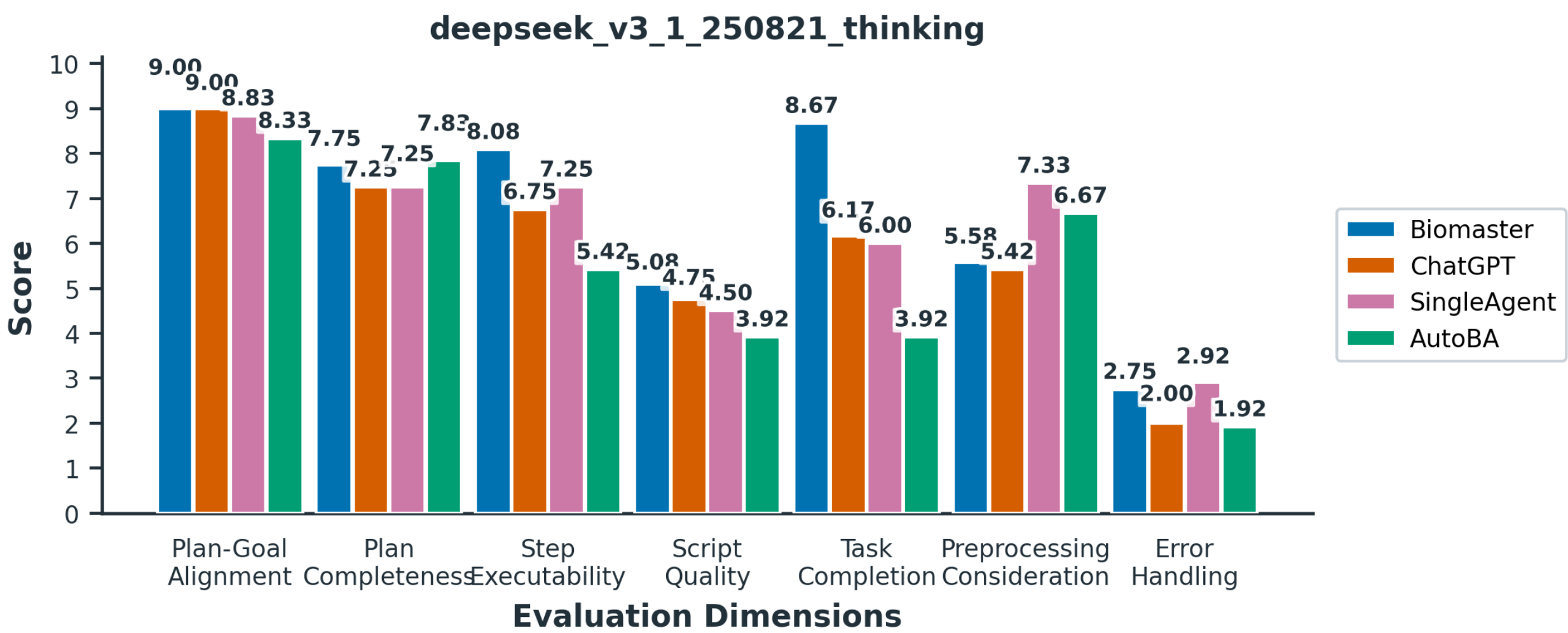

Figure S1: **Per-rater results using DeepSeek-V3 (thinking) as the evaluator.** Scores are averaged over the same 12 randomly sampled, jointly executable tasks and the same seven dimensions as in Fig. 6 a, but reflect ratings from this rater only (no averaging across LLM raters).

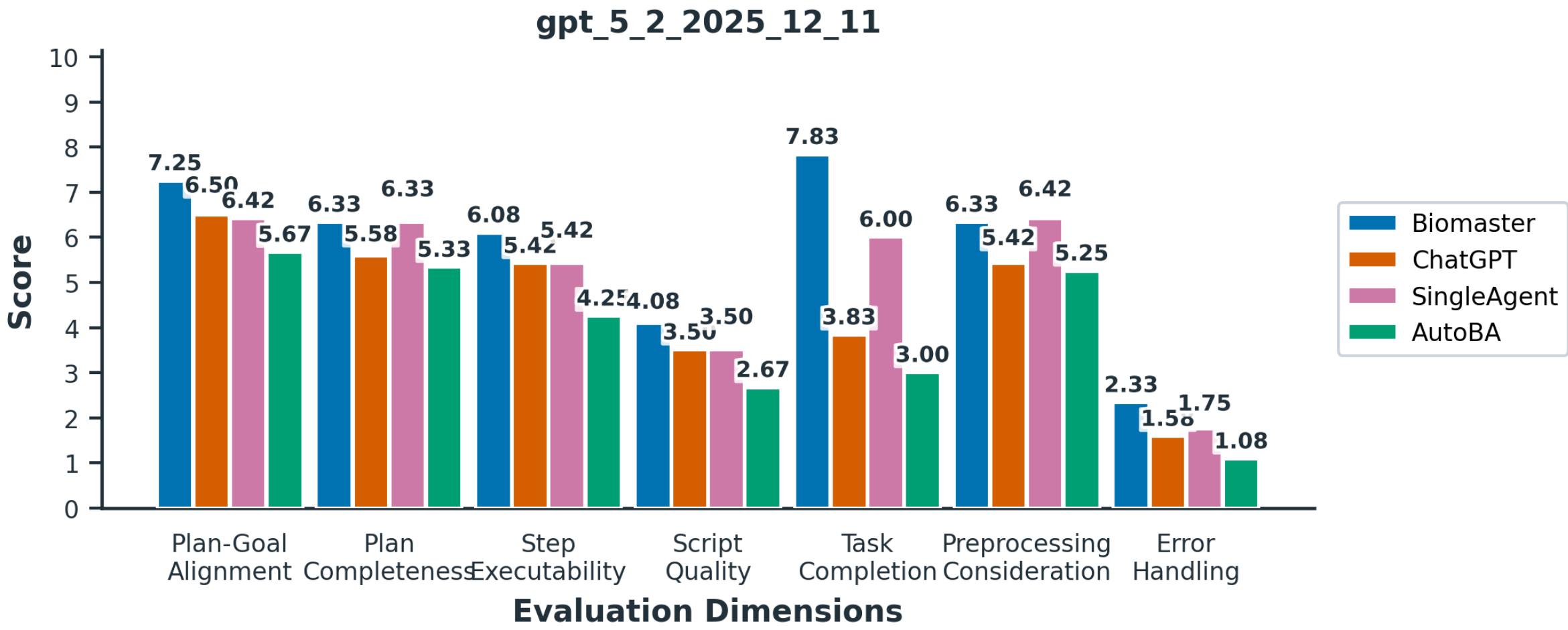

Figure S2: **Per-rater results using GPT-5.2 as the evaluator.** Same task set and seven-dimensional rubric as Fig. 6 a; scores are averaged over tasks for this rater only.

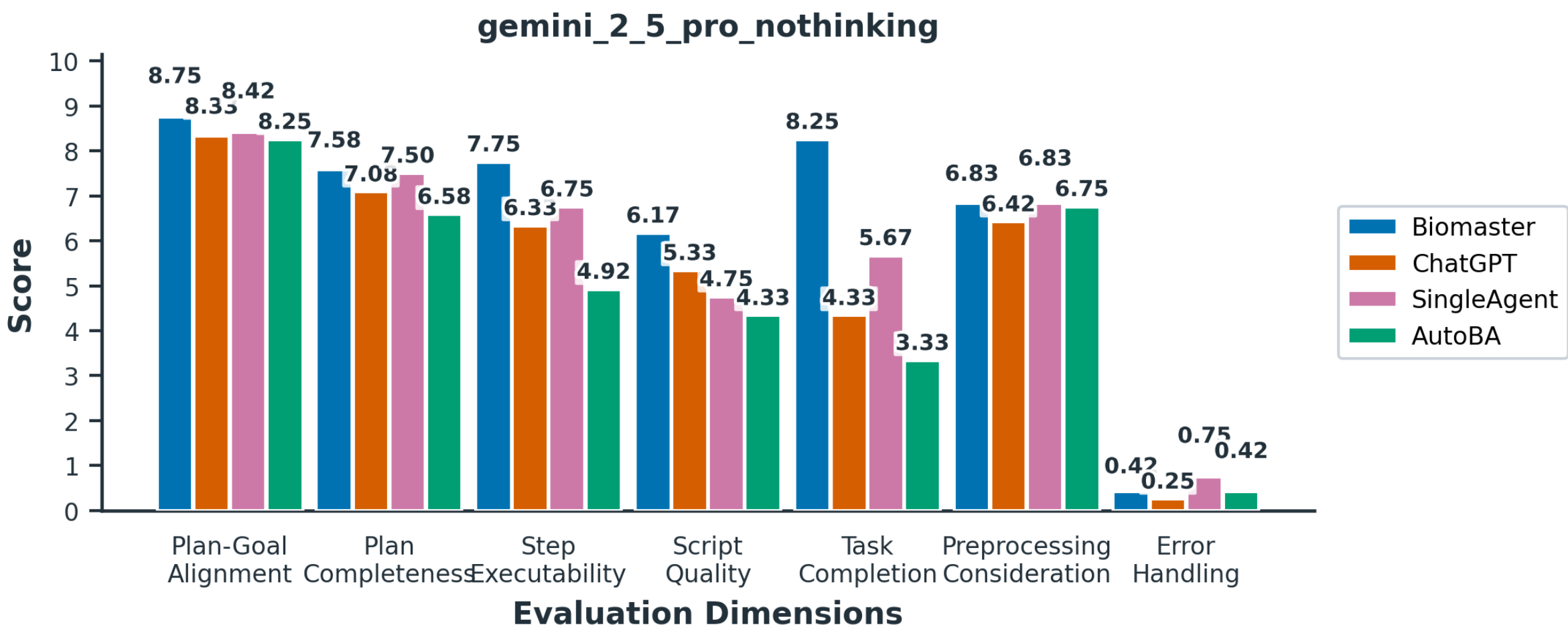

Figure S3: **Per-rater results using Gemini 2.5 Pro (non-thinking) as the evaluator.** Same task set and seven-dimensional rubric as Fig. 6 a; scores are averaged over tasks for this rater only.

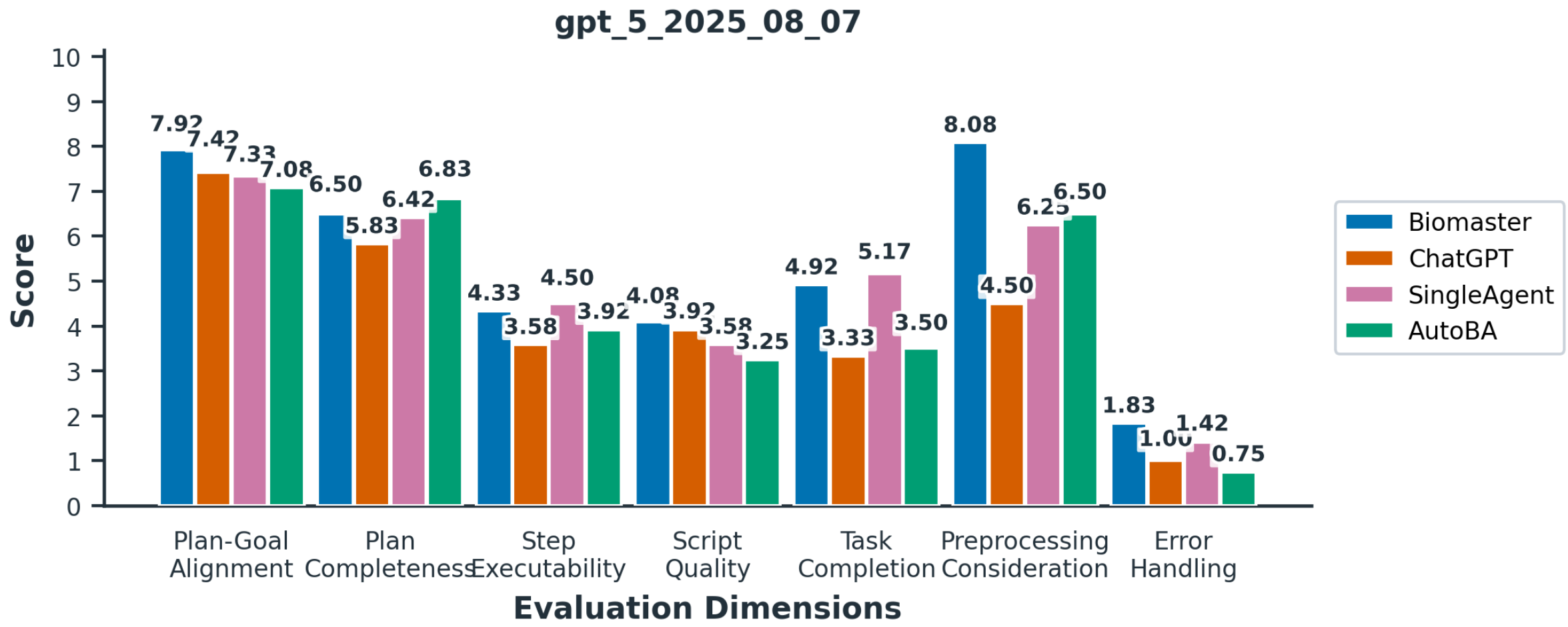

Figure S4: **Per-rater results using GPT-5 as the evaluator.** Same task set and seven-dimensional rubric as Fig. 6 a; scores are averaged over tasks for this rater only.

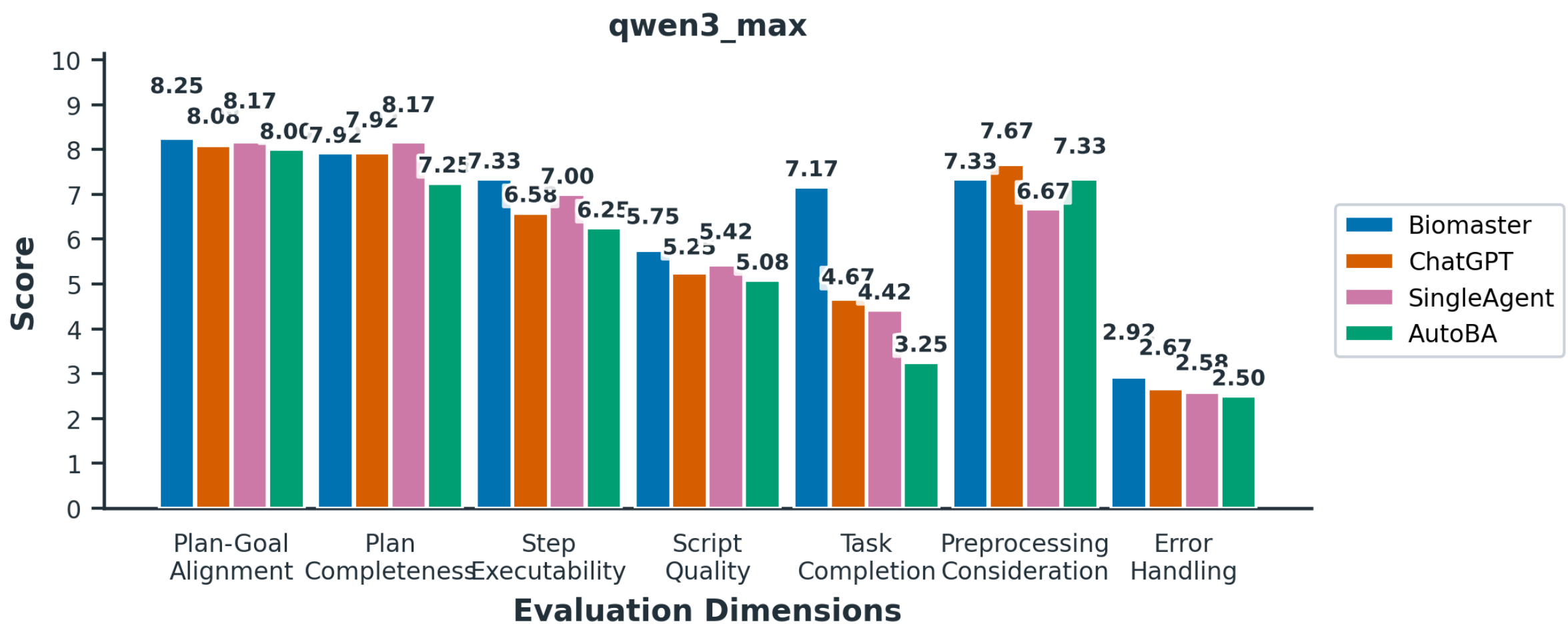

Figure S5: **Per-rater results using Qwen3-Max as the evaluator.** Same task set and seven-dimensional rubric as Fig. 6 a; scores are averaged over tasks for this rater only.

```
You are a bioinformatics analysis evaluator. Your role is to objectively assess the quality of automated
    bioinformatics workflows based on concrete, observable evidence.

## EVALUATION FRAMEWORK

You will evaluate 7 dimensions, each scored 0-10. For each dimension, apply the specific scoring criteria
    below. Base your scores ONLY on evidence present in the provided data.

### DIMENSION 1: Plan-Goal Alignment (0-10)
How well does the analysis plan address the stated biological goal?

SCORING CRITERIA:
- 9-10: Plan directly addresses the goal with scientifically appropriate methods; clear logical flow from goal
     to analysis steps
- 7-8: Plan addresses the goal with suitable methods; minor gaps in logic or method selection
- 5-6: Plan partially addresses the goal; some methods appropriate but others questionable or missing key
    connections
- 3-4: Plan weakly connected to goal; several inappropriate method choices or logical gaps
- 1-2: Plan barely relates to the stated goal; most methods inappropriate
- 0: No plan provided or completely unrelated to goal

### DIMENSION 2: Plan Completeness (0-10)
Does the workflow include all necessary analysis steps?

SCORING CRITERIA:
- 9-10: Includes data QC, preprocessing, main analysis, validation, and output generation; proper step
    sequencing
- 7-8: Includes most essential steps; 1-2 minor steps missing (e.g., some validation)
- 5-6: Core analysis present but missing important preprocessing or validation steps
- 3-4: Several essential steps missing; incomplete workflow
- 1-2: Only 1-2 steps defined; most workflow missing
- 0: No workflow structure defined

### DIMENSION 3: Step Executability (0-10)
Can the defined steps actually be executed?

SCORING CRITERIA:
- 9-10: All steps have correct tool calls, parameters, input/output specifications; dependencies handled
- 7-8: Most steps executable; minor parameter or path issues in 1-2 steps
- 5-6: Some steps executable; others have missing inputs, wrong parameters, or dependency issues
- 3-4: Many steps have execution problems; significant configuration errors
- 1-2: Most steps cannot execute due to missing dependencies, wrong syntax, or invalid parameters
- 0: No executable steps defined

### DIMENSION 4: Script Quality (0-10)
What is the quality of generated code/scripts?

SCORING CRITERIA:
- 9-10: Clean, well-organized code; proper variable naming; comments present; follows bioinformatics
    conventions
- 7-8: Readable code; mostly follows conventions; adequate but not comprehensive documentation
- 5-6: Functional code with readability issues; minimal comments; some convention violations
- 3-4: Poorly organized code; hard to follow; missing documentation; many style issues
- 1-2: Very poor code quality; barely functional; no documentation
- 0: No code/scripts provided

### DIMENSION 5: Task Completion (0-10)
How much of the planned workflow was successfully executed?

SCORING CRITERIA:
- 9-10: All planned steps completed; expected outputs generated; results align with goal
- 7-8: Most steps completed (>75%); main outputs generated; minor issues in some steps
- 5-6: Partial completion (50-75%); some outputs generated; workflow partially achieved goal
- 3-4: Limited completion (25-50%); few outputs; significant execution failures
- 1-2: Minimal completion (<25%); almost no useful outputs
- 0: No steps completed or immediate failure

```

```
### DIMENSION 6: Preprocessing Consideration (0-10)
Is data quality and preparation adequately addressed?

SCORING CRITERIA:
- 9-10: Comprehensive QC (FastQC or equivalent); appropriate filtering/trimming; normalization when needed
- 7-8: QC step present; most preprocessing appropriate; minor gaps
- 5-6: Basic preprocessing mentioned but incomplete; some QC but not comprehensive
- 3-4: Minimal preprocessing; missing critical QC steps; inappropriate methods
- 1-2: Almost no preprocessing; data quality not considered
- 0: No preprocessing when clearly required by data type

### DIMENSION 7: Error Handling (0-10)
How does the system handle errors and failures?

SCORING CRITERIA:
- 9-10: Explicit error checking; logging present; evidence of successful error recovery or debugging
- 7-8: Some error handling; debugging attempts visible; partial recovery from issues
- 5-6: Basic error handling; some log information; limited debugging evidence
- 3-4: Minimal error handling; errors cause workflow to stop; little debugging
- 1-2: Almost no error handling; failures not addressed
- 0: No error handling mechanisms; no evidence of debugging

## EVALUATION PRINCIPLES

1. EVIDENCE-BASED: Score only based on what is explicitly shown in the data. Do not assume or infer
     capabilities not demonstrated.

2. INDEPENDENT DIMENSIONS: Evaluate each dimension separately. A poor score in one dimension should not
     automatically lower scores in others.

3. CALIBRATED SCORING: Use the full 0-10 scale. Avoid clustering scores around any particular range.

4. OBJECTIVE ASSESSMENT: Apply the same criteria consistently. Avoid bias from task complexity or agent type.

OUTPUT FORMAT: Return exactly 7 integers (0-10) separated by single spaces.
Example: 7 6 5 4 6 5 3
```

Listing S1: Evaluator prompt for bioinformatics tasks. The rubric defines seven scoring dimensions (plan–goal alignment, plan completeness, step executability, script quality, task completion, preprocessing consideration, error handling) and enforces an evidence-based 0–10 scoring scheme without default-high anchors.

2 Quantifying Token Usage, Working Time, and Thinking Time

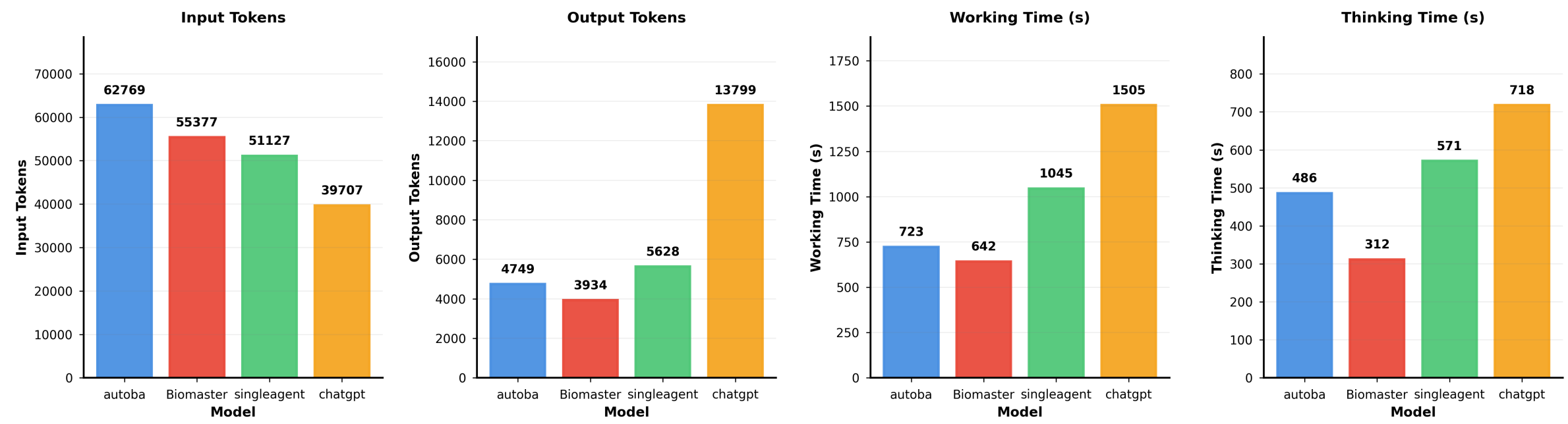

Figure S6: Resource overhead across agents on five representative tasks. Bar charts report, from left to right, Input Tokens, Output Tokens, Working Time (wall-clock seconds for code execution), and Thinking Time (seconds spent awaiting LLM API responses) for AutoBA, BioMaster, SingleAgent, and ChatGPT. Metrics are aggregated over successful runs of five tasks common to all systems: single-cell marker gene identification, single-cell clustering, RNA-seq differential expression, ChIP-seq peak calling, and motif discovery (five runs per task per system). Under identical tool-level knowledge, BioMaster shows lower token usage and shorter working/thinking times than AutoBA and SingleAgent, while ChatGPT exhibits the largest Output Tokens, Working Time, and Thinking Time. Note that these measurements are restricted to tasks completed by all systems; on more complex workflows where baselines fail, their token/time usage can increase due to additional retries and is not reflected here.

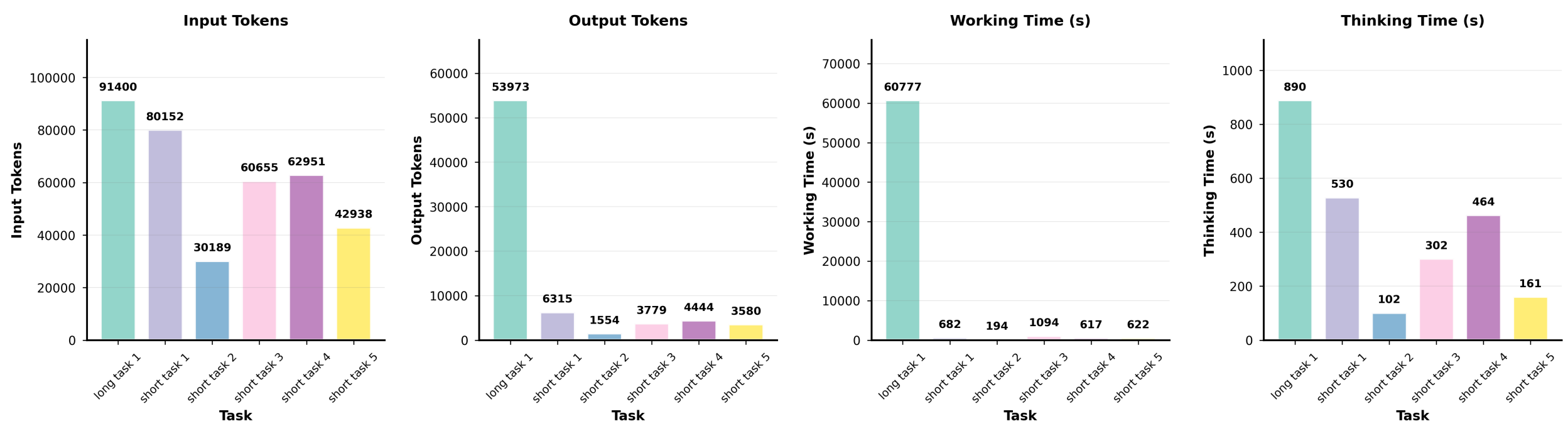

Figure S7: **Computational overhead profiling on representative bioinformatics workflows.** We report four metrics: Input Tokens and Output Tokens (LLM communication cost), Thinking Time (time spent on model reasoning and API responses), and Working Time (wall-clock time consumed by actual bioinformatics tool execution). Five short routine tasks (short tasks 1–5) and one full real-world workflow (long task 1; Hi-C data analysis with multi-stage preprocessing and feature extraction) were evaluated. The results show that, in realistic scenarios, Working Time overwhelmingly dominates total runtime (e.g., 60,777s vs. 890s Thinking Time), confirming that the computational burden primarily arises from underlying bioinformatics programs rather than LLM orchestration. In contrast, short synthetic tasks exhibit a higher proportional contribution from Thinking Time, since their execution workloads are small. This demonstrates that, for real biological data, the practical bottleneck is computation on the data itself, not token usage or LLM latency.

3 Docker validation

3.1 Docker image identification

We validated BioMaster inside a Docker container built from the repository docker/Dockerfile. The image was built and tagged locally as follows.

Image name: biomaster-docker:latest

Image identifier (sha256):

sha256:fbea0767e7662415273e51deddc42bc1ff46ab1cd9d869704530b089bf3b5619

Build command:

```
docker build -f docker/Dockerfile -t biomaster-docker:latest .
```

Verification command:

```
docker image inspect biomaster-docker:latest --format '{{.Id}}'
```

3.2 Representative workflows re-run in Docker

To address reproducibility under containerization, we re-ran two representative single-cell RNA-seq workflows end-to-end in the Docker container: (i) scRNA-seq clustering (5 steps) and (ii) scRNA-seq marker gene identification (6 steps). For each run, we report the exact execution command, key log excerpts indicating step completion, and the resulting output files.

3.3 Execution commands

All commands were executed from the repository root directory (denoted as `$PROJECT_DIR`).

Workflow 1 (scRNA-seq clustering; workflow ID: `gptsccluster`)

```
cd $PROJECT_DIR
./docker/docker-run.sh gptsccluster.yaml
```

Workflow 2 (marker gene identification; workflow ID: `gptscnew1`)

```
cd $PROJECT_DIR
./docker/docker-run.sh gptscnew1.yaml
```

3.4 Evidence: key log excerpts and output verification

For transparency, we provide trimmed excerpts from the execution logs showing successful completion and the key outputs produced at each step. Host-specific absolute paths and external endpoint strings are omitted in the excerpts below.

Workflow 1 (gptsccluster): key log excerpts (trimmed).

```
Step 1: completed; output: ./output/gptsccluster/adata_raw.h5ad
Step 2: completed; output: ./output/gptsccluster/adata_qc.h5ad
Step 3: completed; output: ./output/gptsccluster/adata_norm.h5ad
Step 4: completed; output: ./output/gptsccluster/adata_dimred.h5ad
Step 5: completed; outputs: ./output/gptsccluster/adata_clustered.h5ad
./output/gptsccluster/cluster_plots.png
BioMaster execution completed.
```

Workflow 1 output files (relative paths):

```
./output/gptsccluster/adata_raw.h5ad
./output/gptsccluster/adata_qc.h5ad
./output/gptsccluster/adata_norm.h5ad
./output/gptsccluster/adata_dimred.h5ad
./output/gptsccluster/adata_clustered.h5ad
./output/gptsccluster/cluster_plots.png
```

Workflow 2 (gptscnew1): key log excerpts (trimmed).

```
Step 1: completed; output: ./output/gptscnew1/filtered_singlecellexpress.h5ad
Step 2: completed; output: ./output/gptscnew1/normalized_singlecellexpress.h5ad
Step 3: completed; output: ./output/gptscnew1/pca_singlecellexpress.h5ad
Step 4: completed; output: ./output/gptscnew1/clustered_singlecellexpress.h5ad
```

```
Step 5: completed; output: ./output/gptscnew1/marker_genes.txt
Step 6: completed; outputs: ./output/gptscnew1/umap_clusters.png
./output/gptscnew1/marker_expression_plots.png
BioMaster execution completed.
```

Workflow 2 output files (relative paths):

```
./output/gptscnew1/filtered_singlecellexpress.h5ad
./output/gptscnew1/normalized_singlecellexpress.h5ad
./output/gptscnew1/pca_singlecellexpress.h5ad
./output/gptscnew1/clustered_singlecellexpress.h5ad
./output/gptscnew1/marker_genes.txt
./output/gptscnew1/umap_clusters.png
./output/gptscnew1/marker_expression_plots.png
```

4 Supplemental Data Sources

Overview

Table S2 lists all publicly available datasets used for BioMaster evaluation across 18 biological data types. Datasets were obtained from ENCODE, NCBI SRA, ENA, and other established repositories.

Table S2: **Data sources applied in BioMaster evaluation.** Complete list of publicly available datasets used for benchmarking BioMaster performance across diverse bioinformatics workflows.

Analysis Type	Data Source Link(s)
CAGE-seq Analysis	1. https://www.encodeproject.org/files/ENCFF465ZRB/<br>2. https://ftp.ebi.ac.uk/pub/databases/gencode/Gencode_human/release_47/gencode.v47.primary_assembly.basic.annotation.gtf.gz
DNase-seq Analysis	1. https://www.encodeproject.org/files/ENCFF038HKP/<br>2. https://www.encodeproject.org/files/ENCFF593IOT/
Protein Expression Quantitative Analysis	https://www.ebi.ac.uk/pride/archive/projects/PXD051229
CLIP-seq Analysis	1. https://www.encodeproject.org/files/ENCFF361AWK/<br>2. https://github.com/timflutre/trimmomatic/blob/master/adapters/TruSeq3-PE-2.fa<br>3. https://github.com/carlsonkPhD/tRNA_Charge-Seq/blob/CodovaElife2022/hg38-mature-tRNAs.fa<br>4. https://www.arb-silva.de/no_cache/download/archive/release_138_1/Exports/<br>5. http://ftp.ensembl.org/pub/release-104/fasta/homo_sapiens/dna/Homo_sapiens.GRCh38.dna.primary_assembly.fa.gz<br>6. https://ftp.ensembl.org/pub/release-104/gtf/homo_sapiens/
Ribo-seq Analysis	1. https://www.ncbi.nlm.nih.gov/sra/?term=SRR2007098<br>2. https://github.com/timflutre/trimmomatic/blob/master/adapters/TruSeq3-SE.fa<br>3. http://ftp.ensembl.org/pub/release-104/fasta/homo_sapiens/dna/Homo_sapiens.GRCh38.dna.primary_assembly.fa.gz<br>4. https://ftp.ensembl.org/pub/release-104/gtf/homo_sapiens/
RIP-seq Analysis	1. https://www.ncbi.nlm.nih.gov/sra/?term=SRR15295695<br>2. https://github.com/timflutre/trimmomatic/blob/master/adapters/TruSeq3-SE.fa<br>3. http://ftp.ensembl.org/pub/release-104/fasta/homo_sapiens/dna/Homo_sapiens.GRCh38.dna.primary_assembly.fa.gz<br>4. https://ftp.ensembl.org/pub/release-104/gtf/homo_sapiens/
Spatial Transcriptomics Analysis	1. https://cf.10xgenomics.com/samples/spatial-exp/1.3.0/Visium_FFPE_Mouse_Brain/Visium_FFPE_Mouse_Brain_fastqs.tar

Table S2: **Data sources applied in BioMaster evaluation.** Complete list of publicly available datasets used for benchmarking BioMaster performance across diverse bioinformatics workflows.

Analysis Type	Data Source Link(s)
	2. https://cf.10xgenomics.com/samples/spatial-exp/1.3.0/Visium_FFPE_Mouse_Brain/Visium_FFPE_Mouse_Brain_image.jpg
WGS/WES Analysis	1. https://www.ncbi.nlm.nih.gov/sra/ERR14089508<br>2. https://www.ncbi.nlm.nih.gov/sra/SRX26690893<br>3. https://ftp.sra.ebi.ac.uk/vol1/fastq/SRR318/003/SRR3182423/SRR3182423_1.fastq.gz<br>4. https://ftp.sra.ebi.ac.uk/vol1/fastq/SRR318/003/SRR3182423/SRR3182423_2.fastq.gz
microRNA Analysis	https://nf-co.re/smrnaseq/2.4.0/
3' End-seq Analysis	https://nf-co.re/smrnaseq/2.4.0/
PacBio RNA-seq Analysis	https://nf-co.re/smrnaseq/2.4.0/
Nanopore Analysis	https://www.ebi.ac.uk/ena/browser/home
Bisulfite-Seq Analysis	1. gs://nigms-sandbox/dna-methyl/Habibi2013_chr6F3A.tar.gz<br>2. gs://nigms-sandbox/dna-methyl/Mus_musculus.GRCm39.dna.chromosome.6.fa
ChIP-seq Analysis	1. https://www.ebi.ac.uk/<br>2. ftp://ftp.sra.ebi.ac.uk/vol1/fastq/SRR620/SRR6204/SRR6204.fastq.gz<br>3. ftp://ftp.sra.ebi.ac.uk/vol1/fastq/SRR620/SRR6204/SRR6205.fastq.gz<br>4. ftp://ftp.sra.ebi.ac.uk/vol1/fastq/SRR620/SRR6204/SRR6206.fastq.gz<br>5. ftp://ftp.sra.ebi.ac.uk/vol1/fastq/SRR620/SRR6204/SRR6207.fastq.gz<br>6. ftp://ftp.sra.ebi.ac.uk/vol1/fastq/SRR620/SRR6204/SRR6208.fastq.gz<br>7. http://hgdownload.cse.ucsc.edu/goldenPath/mm10/bigZips/chromFa.tar.gz
RNA-seq Analysis	1. https://github.com/STAR-Fusion/STAR-Fusion-Tutorial.git<br>2. http://hgdownload.soe.ucsc.edu/goldenPath/mm39/bigZips/mm39.fa.gz

Table S2: **Data sources applied in BioMaster evaluation.** Complete list of publicly available datasets used for benchmarking BioMaster performance across diverse bioinformatics workflows.

Analysis Type	Data Source Link(s)
	3. ftp://ftp.sra.ebi.ac.uk/vol1/fastq/SRR137/001/SRR1374921/SRR1374921.fastq.gz<br>4. ftp://ftp.sra.ebi.ac.uk/vol1/fastq/SRR137/002/SRR1374922/SRR1374922.fastq.gz<br>5. ftp://ftp.sra.ebi.ac.uk/vol1/fastq/SRR137/003/SRR1374923/SRR1374923.fastq.gz<br>6. ftp://ftp.sra.ebi.ac.uk/vol1/fastq/SRR137/004/SRR1374924/SRR1374924.fastq.gz<br>7. http://hgdownload.soe.ucsc.edu/goldenPath/mm39/bigZips/genes/mm39.ncbiRefSeq.gtf.gz<br>8. https://www.ncbi.nlm.nih.gov/sra/?term=SRR1039500<br>9. https://github.com/timflutre/trimmomatic/blob/master/adapters/TruSeq3-SE.fa<br>10. http://ftp.ensembl.org/pub/release-104/fasta/homo_sapiens/dna/Homo_sapiens.GRCh38.dna.primary_assembly.fa.gz<br>11. https://ftp.ensembl.org/pub/release-104/gtf/homo_sapiens/
Hi-C Analysis	https://data.4dnucleome.org/resources/data-analysis/hi_c-processing-pipeline
scRNA-seq Analysis	https://figshare.com/articles/dataset/Kang_HM_Subramaniam_M_Targ_S_Nguyen_M_et_al_2017/19397624
Metagenomic Analysis	https://github.com/taowenmicro/EasyMicrobiomeR

5 Ablation Study: Debug Agent Evaluation

Overview

We performed ablation studies on 5 bioinformatics pipelines to assess the Debug Agent's contribution, running each pipeline 5 times with and without the Debug Agent. The Debug Agent substantially improves execution success, with particularly pronounced gains in ChIP-seq peak calling, WGS/WES somatic analysis, Hi-C processing, and RNA-seq differential expression.

Table S3: **Ablation study on Debug Agent component.** Comparison of BioMaster performance with and without the Debug Agent across 5 bioinformatics pipelines and 5 independent runs each. Success indicates successful execution; fail indicates failed execution; dash (-) indicates step not reached due to upstream failure.

Pipeline	Steps	Case 1	Case 2	Case 3	Case 4	Case 5	Total Success Rate
CHIP-Seq call peaks BioMaster	quality control and adapter trimming	success	success	success	success	success	1
	Align	success	success	success	success	success	1
	SAM files to sorted BAM	success	success	success	success	success	1
	Remove PCR duplicates	success	success	success	success	success	1
	call peak	success	success	success	success	success	1
CHIP-Seq call peaks BioMaster w/o Debug	quality control and adapter trimming	success	success	success	success	success	1
	Align	fail	fail	fail	success	success	0.4
	SAM files to sorted BAM	-	-	-	fail	fail	0
	Remove PCR duplicates	-	-	-	-	-	0
	call peak	-	-	-	-	-	0
WGS/WES analysis Somatic BioMaster	quality control and adapter trimming	success	success	success	success	success	1
	Align	success	success	success	success	success	1
	SAM files to sorted BAM	success	success	success	success	success	1
	Remove PCR duplicates	success	fail	success	success	success	0.8
	somatic SNV and indel calling	success	-	success	success	success	0.8
WGS/WES analysis Somatic BioMaster w/o Debug	quality control and adapter trimming	fail	success	success	success	success	0.8
	Align	-	success	success	fail	fail	0.4
	SAM files to sorted BAM	-	success	success	-	-	0.4
	Remove PCR duplicates	-	fail	fail	-	-	0
	somatic SNV and indel calling	-	-	-	-	-	0
HIC Data Analysis BioMaster	align	fail	success	success	success	success	0.8
	Parse and sort the unsorted BAM files	-	success	success	success	success	0.8
	Merge the two pairsam files	-	success	fail	success	success	0.6
	Mark duplicate reads in the merged pairsam file	-	success	-	success	success	0.6
	Filter and annotate the duplicate-marked pairsam file	-	success	-	success	success	0.6
	Merge the final pairs files	-	success	-	success	success	0.6

Continued on next page

Table S3 – continued from previous page

Pipeline	Steps	Case 1	Case 2	Case 3	Case 4	Case 5	Total Success Rate
	Create an unnormalized contact matrix	-	success	-	success	success	0.6
	Balance (normalize) the contact matrix	-	success	-	success	success	0.6
	Generate a multi-resolution	-	success	-	success	success	0.6
HIC Data Analysis BioMaster w/o Debug	align	fail	fail	fail	success	success	0.4
	Parse and sort the unsorted BAM files	-	-	-	fail	success	0.2
	Merge the two pairsam files	-	-	-	-	fail	0
	Mark duplicate reads in the merged pairsam file	-	-	-	-	-	0
	Filter and annotate the duplicate-marked pairsam file	-	-	-	-	-	0
	Merge the final pairs files	-	-	-	-	-	0
	Create an unnormalized contact matrix	-	-	-	-	-	0
	Balance (normalize) the contact matrix	-	-	-	-	-	0
	Generate a multi-resolution	-	-	-	-	-	0
RNA Identify top5 down regulated genes BioMaster	quality control and adapter trimming	success	success	success	success	success	1
	Align	success	success	success	success	success	1
	SAM files to sorted BAM	success	success	success	success	success	1
	Remove PCR duplicates	success	success	success	success	success	1
	Quantify gene expression	success	success	success	success	success	1
	Conduct differential expression analysis	success	success	success	success	success	1
	Filter and rank the differential expression result	success	success	success	success	success	1
RNA Identify top5 down regulated genes BioMaster w/o Debug	quality control and adapter trimming	fail	success	success	success	success	0.8
	Align	-	fail	fail	fail	success	0.2
	SAM files to sorted BAM	-	-	-	-	success	0.2
	Remove PCR duplicates	-	-	-	-	success	0.2
	Quantify gene expression	-	-	-	-	fail	0
	Conduct differential expression analysis	-	-	-	-	-	0
	Filter and rank the differential expression result	-	-	-	-	-	0

6 Comparative Analysis: Hi-C Data Processing

Overview

Tables S4–S8 compare system performance on Hi-C data analysis, covering alignment, pair processing, filtering, contact matrix generation, and normalization. BioMaster completes all 9 workflow steps, while ChatGPT and AutoBA fail at step 5 (filter and annotate) due to incorrect pairtools usage.

Table S4: **Hi-C data analysis comparison (Part 1).** Comparison of alignment and initial processing steps across three systems. ✓ indicates successful step completion; × indicates failure.

**Hi-C analysis**	**Alignment**	**Parse & sort BAM**	**Merge pairsam files**	**Mark duplicates**
Tools	bwa	samtools, pairtools	pairtools	pairtools, pairix
ChatGPT	✓	✓	✓	✓
AutoBA	✓	✓	✓	✓
BioMaster	✓	✓	✓	✓

Table S5: **Hi-C data analysis comparison (Part 2).** Comparison of advanced processing steps including filtering, matrix generation, and normalization. Only BioMaster successfully completes all downstream analysis steps.

**Hi-C analysis**	**Filter & annotate**	**Merge pairs files**	**Create contact matrix**	**Balance matrix**	**Generate multi-resolution**
Tools	pairtools	pairix	cooler	cooler	cooler
ChatGPT	wrongly use pairtools	×	×	×	×
AutoBA	wrongly use pairtools	×	×	×	×
BioMaster	✓	✓	✓	✓	✓

Table S6: **Hi-C data mapping and sorting.** All three systems successfully perform basic alignment and BAM sorting operations.

**Hi-C data mapping, sorting and conversion**	**Alignment**	**BAM sorting**
Tools	bwa	samtools
ChatGPT	✓	✓
AutoBA	✓	✓
BioMaster	✓	✓

Table S7: **Hi-C contact matrix processing.** ChatGPT fails at matrix creation due to incorrect cooler settings; AutoBA and BioMaster succeed at all steps.

**Hi-C data contact matrix processing**	**Create unnormalized matrix**	**Balance matrix**	**Generate multi-resolution cooler files**
Tools	cooler	cooler	cooler
ChatGPT	wrongly use cooler setting	×	×
AutoBA	✓	✓	✓
BioMaster	✓	✓	✓

Table S8: **Hi-C pair parsing and cleaning.** BioMaster demonstrates superior handling of pairtools and pairix workflows; ChatGPT and AutoBA fail at filtering and merging steps.

**Hi-C data pair parsing and cleaning**	**Merge pairsam files**	**Mark duplicates**	**Filter & annotate**	**Merge pairs files**
Tools	pairtools	pairtools, pairix	pairtools	pairix
ChatGPT	✓	wrongly use pairtools	×	×
AutoBA	✓	✓	wrongly use pairtools	×
BioMaster	✓	✓	✓	✓

7 Comparative Analysis: ChIP-seq Data Processing

Overview

Tables S9–S13 evaluate ChIP-seq workflows including peak calling, functional enrichment, and motif discovery. All systems complete basic peak calling and motif discovery, but only BioMaster successfully performs functional enrichment analysis with peak annotation, differential binding, and pathway enrichment.

Table S9: **ChIP-seq peak calling workflow.** All three systems successfully complete the standard ChIP-seq peak calling pipeline.

**ChIP-seq peak calling**	**Quality control & trimming**	**Alignment**	**Sort & remove duplicates**	**Peak calling**
Tools	FastQC, Trimmomatic	BWA-MEM	SAMtools, Picard	MACS3
ChatGPT	✓	✓	✓	✓
AutoBA	✓	✓	✓	✓
BioMaster	✓	✓	✓	✓

Table S10: **ChIP-seq functional enrichment analysis (Part 1).** Initial processing steps: ChatGPT encounters errors in SAM conversion; AutoBA fails at signal track generation.

Functional Enrichment (Part 1)	Quality control	Alignment	SAM to BAM	Signal tracks
Tools	FastQC, Trimmomatic, Cutadapt	BWA	samtools	deeptools
ChatGPT	✓	✓	wrong bwa output file	×
AutoBA	✓	✓	✓	wrong use deeptools
BioMaster	✓	✓	✓	✓

Table S11: **ChIP-seq functional enrichment analysis (Part 2).** Advanced analysis steps: only BioMaster successfully completes peak calling, annotation, differential binding, and enrichment analysis.

Functional Enrichment (Part 2)	Call peaks	Annotate peaks	Differential binding	Enrichment analysis
Tools	MACS3	HOMER	DESeq2	ClusterProfiler
ChatGPT	×	×	×	×
AutoBA	×	×	×	×
BioMaster	✓	✓	✓	✓

Table S12: **ChIP-seq motif discovery (Part 1).** All systems successfully complete initial processing for motif discovery workflow.

Motif Discovery	Quality control	Alignment	SAM to BAM	Signal tracks
Tools	FastQC, Trimmomatic, Cutadapt	BWA	samtools	deeptools
ChatGPT	✓	✓	✓	✓
AutoBA	✓	✓	✓	✓
BioMaster	✓	✓	✓	✓

Table S13: **ChIP-seq motif discovery (Part 2).** All systems successfully complete peak calling, annotation, and motif discovery using HOMER.

**Motif Discovery**	**Call peaks**	**Annotate peaks**	**Discover DNA motifs**
Tools	MACS3	HOMER	HOMER
ChatGPT	✓	✓	✓
AutoBA	✓	✓	✓
BioMaster	✓	✓	✓

8 Comparative Analysis: RNA-seq Data Processing

Overview

Tables S14–S21 cover 10 RNA-seq analysis types: alternative polyadenylation (APA), differential expression, isoform quantification, fusion detection, and RNA editing. BioMaster completes 9/10 tasks, compared to ChatGPT (5/10) and AutoBA (4/10). APA analysis remains challenging for all systems.

Table S14: **RNA-seq alternative polyadenylation (APA) analysis (Part 1).** ChatGPT and AutoBA fail at alignment step; BioMaster successfully completes initial processing.

RNA-seq APA (Part 1)	Alignment	SAM to BAM	Index BAM	Read depth profile
Tools	STAR	samtools	samtools	samtools
ChatGPT	×	×	×	×
AutoBA	Keep thinking	×	×	×
BioMaster	✓	✓	✓	✓

Table S15: **RNA-seq APA analysis (Part 2).** Downstream APA quantification steps; BioMaster successfully extracts regions and quantifies APA events.

RNA-seq APA (Part 2)	Extract genomic regions	Quantify APA
Tools	R	R
ChatGPT	×	×
AutoBA	×	×
BioMaster	✓	✓

Table S16: **RNA-seq differential gene expression analysis.** All systems successfully complete the standard differential expression workflow.

Differential Expression	Quality control	Alignment	SAM to BAM	Gene expression quantification	Differential analysis
Tools	Trimmomatic	BWA	samtools	HTSeq	DESeq2
ChatGPT	✓	✓	✓	✓	✓
AutoBA	✓	✓	✓	✓	✓
BioMaster	✓	✓	✓	✓	✓

Table S17: **RNA-seq top downregulated genes identification (Part 1).** All systems successfully complete initial processing steps.

**Top Downregulated Genes identification**	**Quality control**	**Alignment**	**SAM to BAM**	**Sort BAM**
Tools	Trimmomatic	BWA	samtools	samtools
ChatGPT	✓	✓	✓	✓
AutoBA	✓	✓	✓	✓
BioMaster	✓	✓	✓	✓

Table S18: **RNA-seq top downregulated genes identification (Part 2).** All systems successfully complete gene quantification, differential analysis, and result filtering.

**Top Downregulated Genes identification**	**Gene expression quantification**	**Differential expression analysis**	**Filter and rank results**
Tools	HTSeq	DESeq2	R
ChatGPT	✓	✓	✓
AutoBA	✓	✓	✓
BioMaster	✓	✓	✓

Table S19: **RNA-seq isoform expression analysis.** ChatGPT fails at STAR alignment; AutoBA and BioMaster successfully complete isoform quantification.

**RNA Isoform Expression**	**Quality control**	**Transcriptome alignment**	**Convert SAM**	**Isoform quantification**
Tools	Trimmomatic	STAR	Samtools	RESM
ChatGPT	✓	failed use STAR	×	×
AutoBA	✓	✓	✓	✓
BioMaster	✓	✓	✓	✓

Table S20: **RNA-seq fusion gene prediction.** All systems successfully complete fusion gene detection using STAR-Fusion.

**RNA-seq Fusion Gene Prediction**	**RNA-seq read alignment**	**Fusion genes detection**
Tools	STAR	STAR-Fusion
ChatGPT	✓	✓
AutoBA	✓	✓
BioMaster	✓	✓

Table S21: **RNA-seq editing analysis.** AutoBA gets stuck at alignment step; ChatGPT and BioMaster successfully identify RNA editing events.

**RNA Editing**	**Quality control**	**Transcriptome alignment**	**SAM to sorted BAM**	**Variant calling**	**Identify editing events**
Tools	Trimmomatic	STAR	Samtools	Samtools	R
ChatGPT	✓	✓	✓	✓	✓
AutoBA	✓	Keep thinking	×	×	×
BioMaster	✓	✓	✓	✓	✓

9 Comparative Analysis: WGS/WES Data Processing

Overview

Tables S22–S24 evaluate WGS/WES workflows including differential expression, somatic variant calling, and variant annotation. All systems complete differential expression and somatic variant calling. ChatGPT fails at variant annotation due to incorrect GATK Funcotator usage.

Table S22: **WGS differential gene expression analysis.** All systems successfully complete transcriptome alignment and differential analysis.

WGS Differential Expression	Transcriptome alignment	Sort & index BAM	Differential expression analysis
Tools	STAR	Samtools	DESeq2
ChatGPT	✓	✓	✓
AutoBA	✓	✓	✓
BioMaster	✓	✓	✓

Table S23: **WGS/WES somatic variant calling.** All systems successfully complete the full somatic variant calling pipeline.

Somatic Variant Calling	Genome alignment	SAM to sorted BAM	Mark duplicates	SNV & indel calling	Filter variants
Tools	STAR	samtools	GATK	GATK	GATK
ChatGPT	✓	✓	✓	✓	✓
AutoBA	✓	✓	✓	✓	✓
BioMaster	✓	✓	✓	✓	✓

Table S24: **WGS/WES somatic variant annotation.** ChatGPT fails at GATK Funcotator usage; AutoBA and BioMaster successfully annotate variants.

Somatic Variant Annotation	RNA-seq alignment	SAM conversion	Sort & index BAM	SNV & indel calling	Variant annotation
Tools	STAR	Samtools	Samtools	GATK	GATK Funcotator
ChatGPT	✓	✓	✓	✓	wrong use GATK Funcotator
AutoBA	✓	✓	✓	✓	✓
BioMaster	✓	✓	✓	✓	✓

10 Comparative Analysis: microRNA and Long-read Sequencing

Overview

Tables S25–S31 evaluate microRNA analysis and long-read sequencing (Nanopore, PacBio). For microRNA quantification, only BioMaster succeeds (ChatGPT and AutoBA fail at bowtie alignment). For miRNA prediction, ChatGPT and BioMaster succeed while AutoBA incorrectly uses miRDeep2. Only BioMaster completes the full PacBio RNA-seq pipeline. Polyadenylation site (PAS) identification remains challenging for all systems.

Table S25: **MicroRNA quantification.** ChatGPT and AutoBA fail at alignment using bowtie; BioMaster successfully quantifies miRNA expression.

**microRNA quantification**	**quality control and trim using FastQC**	**alignment and trim reads to reference sequence**	**Quantify miRNA expression**
Tools	FastQC, fastq	bowtie1, samtools	featureCounts
ChatGPT	✓	failed to align using bowtie2	×
AutoBA	✓	wrongly use bowtie2 to read genome.fa	×
BioMaster	✓	✓	✓

Table S26: **MicroRNA prediction.** AutoBA incorrectly uses miRDeep2; ChatGPT and BioMaster successfully identify novel miRNAs.

**microRNA prediction**	**quality control and trim using FastQC**	**alignment and trim reads to reference genome**	**Quantify miRNA expression levels**	**Identify novel miRNAs using miRDeep2**
Tools	FastQC, fastq	bowtie1, samtools	featureCounts	miRDeep2
ChatGPT	✓	✓	✓	✓
AutoBA	✓	✓	Wrongly Use miRDeep2 to quantify known miRNAs	×
BioMaster	✓	✓	✓	✓

Table S27: **Nanopore RNA-seq analysis (Part 1).** AutoBA fails at minimap2 usage; ChatGPT and BioMaster complete isoform expression analysis.

**Isoform Expression Analysis**	**Align reads to the human reference genome**	**Convert and sort the SAM file to BAM format**	**Use StringTie to reconstruct transcripts and quantify their expression**
Tools	minimap2	Samtools	StringTie
ChatGPT	✓	✓	✓
AutoBA	Wrongly use minimap2 to read file	×	×
BioMaster	✓	✓	✓

Table S28: **BioMaster plan for Nanopore RNA-seq (Part 2).** Detailed workflow showing quality control, alignment, and quantification steps.

**Isoform Expression Analysis**	**Perform internal quality control and adapter trimming**	**Align the trimmed Nanopore reads to reference genome**	**Convert SAM to BAM format**	**Sort and index the BAM file**	**Quantify transcript isoform expression**
Tools	internal_trim_qc	minimap2	samtools	samtools	featureCounts
BioMaster	✓	✓	✓	✓	✓

Table S29: **PacBio RNA-seq analysis.** ChatGPT fails at SAM conversion; AutoBA fails at quality assessment; BioMaster completes full pipeline.

**PacBio RNA sequencing data analysis**	**Perform internal quality control and adapter trimming**	**Align the trimmed reads to reference genome**	**Convert SAM to BAM format**	**Sort and index the BAM file**	**Quantify transcript expression**
Tools	internal_trim_qc	minimap2	samtools	samtools	featureCounts
ChatGPT	✓	✓	failed to use samtool to convert format	×	×
AutoBA	Wrongly use manaplot for quality assessment	×	×	×	×
BioMaster	✓	✓	✓	✓	✓

Table S30: **PAS identification (Part 1).** AutoBA fails at adapter trimming; ChatGPT cannot identify high-confidence PAS.

**PAS (polyadenylation site) identification**	**Quality control & adapter trimming**	**Remove trailing polyA sequences**	**Cluster reads by similarity**	**Build consensus sequences**	**Identify high-confidence PAS**
Tools	Trimmomatic	Cutadapt	VSEARCH	NA	NA
ChatGPT	✓	✓	✓	✓	cannot identify PAS
AutoBA	Wrongly use trim galore	×	×	×	×

Table S31: **PAS identification (Part 2).** BioMaster workflow for PAS clustering; limited success in final PAS identification.

**PAS (polyadenylation site) identification**	**Quality control & adapter trimming**	**Extract last 20nt from reads**	**Cluster similar 3′ end sequences**
Tools	fastp	extract_3p_end	cluster_pas_peaks
BioMaster	✓	✓	cannot identify PAS

Table S32: **RNA-seq expression quantification.** ChatGPT and AutoBA fail at alignment; BioMaster successfully quantifies expression.

RNA-Seq Expression quantification	Quality control	Trimming	Aligning	Quantifying expression
Tools	FastQC	Trimmomatic	STAR/Hisat2	featureCounts
ChatGPT	✓	✓	failing in using STAR	×
AutoBA	✓	✓	failing in using Hisat2	×
BioMaster	✓	✓	✓	✓

Table S33: **RNA-seq functional annotation and enrichment.** Only BioMaster completes full functional annotation pipeline.

RNA-Seq Functional annotation	Quality control	Trimming	Aligning	Counting reads	Differential expression	Functional annotation & enrichment
Tools	FastQC	Trimmomatic	STAR/HISAT2	featureCounts	DESeq2	clusterProfiler
ChatGPT	✓	✓	failing in using STAR	×	×	×
AutoBA	✓	✓	failing in using Hisat2	×	×	×
BioMaster	✓	✓	✓	✓	✓	✓

Table S34: **Circular RNA identification.** ChatGPT and AutoBA fail at BWA alignment; BioMaster successfully identifies circRNAs.

**Circular RNA identification**	**Quality control**	**Trimming**	**Aligning**	**Indexing**	**Identifying candidate circular RNAs**
Tools	FastQC	Trim Galore	STAR/BWA	samtools	CIRCexplorer2/CIRIquant
ChatGPT	✓	✓	failing using BWA	×	×
AutoBA	✓	✓	failing in using BWA	×	×
BioMaster	✓	✓	✓	✓	✓

11 Comparative Analysis: Specialized RNA Sequencing Techniques

Overview

Tables S32–S38 cover specialized RNA techniques: CLIP-seq, RIP-seq, Ribo-seq, and splicing analysis. BioMaster completes all tasks successfully. ChatGPT and AutoBA encounter various failures including incorrect tool selection (CLIPper, RiboCode, rMATS), alignment errors, and peak annotation issues.

Table S35: **CLIP-seq crosslink identification.** ChatGPT fails at CLIPper usage; AutoBA returns invalid response; BioMaster succeeds.

**Clipseq**	**Adapter and Quality Trimming**	**Pre-mapping to rRNA and tRNA**	**Genome Mapping**	**UMI-Based Deduplication**	**Crosslink Identification**
Tools	Cutadapt	Bowtie2	Bowtie2	UMI-tools	BEDTools
ChatGPT	✓	✓	✓	✓	fail in using CLIPper to recognize cross link sites
AutoBA	✓	✓	✓	✓	invalid response in reading file
BioMaster	✓	✓	✓	✓	✓

Table S36: **RIP-seq RBP-bound gene identification.** ChatGPT and AutoBA fail at peak annotation; BioMaster completes full analysis.

**RIP-seq**	**Adapter and Quality Trimming**	**Genome Mapping**	**Convert SAM to sorted BAM**	**Identify potential RBP binding sites**	**Annotate the detected peaks**	**Extract enriched genes**
Tools	Cutadapt	Bowtie2	Samtools	pureclip	BEDTools	BEDTools
ChatGPT	✓	✓	✓	✓	fail in using MACS2 to recognize called peaks for RBP-bound regions	×
AutoBA	✓	✓	✓	✓	fail in using DESeq2 in R	×
BioMaster	✓	✓	✓	✓	✓	✓

Table S37: **Ribo-seq ORF detection.** ChatGPT incorrectly uses RiboCode; AutoBA fails at alignment; BioMaster succeeds.

**Ribo-seq**	**Adapter and Quality Trimming**	**Genome Mapping**	**Convert SAM to sorted BAM**	**Identify actively translated ORFs**
Tools	Trimmomatic	Bowtie2	Samtools	ribotricer
ChatGPT	✓	✓	✓	Wrongly use RiboCode to identify translated ORFs
AutoBA	✓	fail in using bowtie2	×	×
BioMaster	✓	✓	✓	✓

Table S38: **RNA-seq splicing analysis.** ChatGPT and AutoBA fail at rMATS usage; BioMaster successfully identifies alternative splicing.

**RNA-seq Splicing analysis**	**Adapter and Quality Trimming**	**Genome Mapping**	**Run rMATS to identify alternative splicing events**
Tools	Trimmomatic	Bowtie2	rMATS
ChatGPT	✓	✓	fail in use rMATS to recognize alternative splicing
AutoBA	✓	✓	×, no output file
BioMaster	✓	✓	✓

12 Comparative Analysis: Epigenomics and Proteomics

Overview

Tables S39–S42 evaluate epigenomics (WGS/WES structural variants, DNase-seq, CAGE-seq) and proteomics (mass spectrometry). BioMaster completes all analyses. ChatGPT and AutoBA fail across all tasks due to incorrect tool usage (Delly, samtools, CAGEr, limma).

Table S39: **WGS/WES structural variation.** ChatGPT and AutoBA encounter tool usage errors; BioMaster successfully detects SVs.

**WGS/WES Structure variation identification**	**Trim low-quality bases**	**Alignment**	**Parse & sort**	**Structural variant detection**
Tools	Trimmomatic	BWA	samtools	Delly/cuteSV
ChatGPT	✓	✓	✓	Wrongly use Delly
AutoBA	✓	Wrongly use BWA	×	×
BioMaster	✓	✓	✓	✓

Table S40: **DNase-seq analysis.** ChatGPT fails at samtools usage; AutoBA fails at trimming; BioMaster completes analysis.

DNase-seq	Quality control and adapter trimming	Alignment	Parse & sort	Index the sorted BAM file	Identify
Tools	Trimmomatic	BWA	samtools	samtools	MACS2
ChatGPT	✓	✓	wrongly use samtools	×	×
AutoBA	Wrongly use trimmomatic	×	×	×	×
BioMaster	✓	✓	✓	✓	✓

Table S41: **CAGE-seq analysis.** ChatGPT incorrectly uses CAGEr; AutoBA fails at hisat2; BioMaster succeeds.

CAGE-seq	Parse & sort	Index the sorted BAM file	Peak calling	differential expression analysis
Tools	samtools/hisat2	samtools	CAGEr	EdgeR/DESeq2
ChatGPT	✓	✓	Wrongly use CAGEr	×
AutoBA	Wrongly use hisat2	×	×	×
BioMaster	✓	✓	✓	✓

Table S42: **Mass spectrometry analysis.** ChatGPT fails at normalization; AutoBA incorrectly uses limma; BioMaster completes analysis.

Mass spectrometry data analysis	Data clean	Apply normalization	identify significant expression differences	calculate fold change
Tools	pandas	numpy	scipy/lima	pandas
ChatGPT	✓	Wrongly apply normalization	×	×
AutoBA	✓	✓	✓	Wrongly use limma
BioMaster	✓	✓	✓	✓

13 Comparative Analysis: Spatial Transcriptomics

Overview

Tables S43–S49 evaluate spatial transcriptomics analyses including neighborhood enrichment, cell-type deconvolution, ligand-receptor interactions, and domain identification. BioMaster completes 4/5 tasks; ChatGPT and AutoBA complete 1/5. Cell-type deconvolution fails for all systems due to package conflicts. ChatGPT encounters Python script errors; AutoBA fails at data reading and clustering steps.

Table S43: **Spatial transcriptomics neighborhood enrichment.** ChatGPT has script errors; AutoBA has invalid response; BioMaster succeeds.

**ST - neighborhood enrichment**	**quality control**	**Normalization and Log Transformation**	**compute spatial neighborhood graph**	**neighborhood enrichment**
Tools	scanpy	scanpy	squidpy	squidpy
ChatGPT	✓	error python script	×	×
AutoBA	Invalid response in reading data	×	×	×
BioMaster	✓	✓	✓	✓

Table S44: **Spatial transcriptomics deconvolution.** All systems fail due to package conflicts and data reading issues.

ST Deconvolution	sc data preprocessing	ST preprocessing	Cell type annotation	Deconvolution
Tools	scanpy	squidpy	celltypist	tangram
ChatGPT	error python script	×	×	×
AutoBA	×, Invalid response in reading data	×	×	×
BioMaster	×, package conflicts	×	×	×

Table S45: **Spatial transcriptomics ligand-receptor analysis.** ChatGPT has script errors; AutoBA has invalid response; BioMaster succeeds.

ST Ligand-Receptor	Quality control	Normalization & Log Transform	Compute Neighborhood Graph	Ligand-Receptor Pairs & Scores
Tools	scanpy	scanpy	squidpy	squidpy
ChatGPT	✓	✓	error python script	×
AutoBA	×, Invalid response in reading data	×	×	×
BioMaster	✓	✓	✓	✓

Table S46: **Spatial transcriptomics domain identification (Part 1).** ChatGPT has Python script errors; AutoBA and BioMaster complete initial steps.

domain identification	Quality control	Normalization and Log Transformation	Highly Variable Gene Selection	Dimensionality Reduction
Tools	scanpy	scanpy	scanpy	scanpy
ChatGPT	✓	✓	error python script	×
AutoBA	✓	✓	✓	✓
BioMaster	✓	✓	✓	✓

Table S47: **Spatial transcriptomics domain identification (Part 2).** ChatGPT fails completely; AutoBA encounters clustering errors; BioMaster succeeds.

domain identification	Compute the neighborhood graph	Clustering	Visualization (umap & mapping to tissue)
Tools	squidpy	squidpy	squidpy
ChatGPT	×	×	×
AutoBA	✓	×, 'cluster' key missing	×
BioMaster	✓	✓	✓

Table S48: **Complete spatial transcriptomics analysis (Part 1).** ChatGPT has script errors; AutoBA fails at preprocessing; BioMaster succeeds.

ST Analysis	Preprocessing	Quality control	Normalization and Log Transformation	Highly Variable Gene Selection	Dimensionality Reduction
Tools	scanpy	scanpy	scanpy	scanpy	scanpy
ChatGPT	✓	✓	✓	✓	wrong python script
AutoBA	×, cannot use spaceranger to do preprocessing	×	×	×	×
BioMaster	✓	✓	✓	✓	✓

Table S49: **Complete spatial transcriptomics analysis (Part 2).** Only BioMaster completes neighborhood enrichment, clustering, and variable gene analysis.

ST Analysis	Compute the neighborhood graph	Neighborhood enrichment	Clustering	Spatial Variable Gene Analysis	Visualization
Tools	scanpy	scanpy	squidpy	squidpy	squidpy
ChatGPT	×	×	×	×	×
AutoBA	×	×	×	×	×
BioMaster	✓	✓	✓	✓	✓

14 Comparative Analysis: Single-cell RNA Sequencing

Overview

Tables S50–S59 evaluate scRNA-seq workflows including clustering, differential expression, marker gene identification, cell type annotation, trajectory analysis, and regulatory network inference. BioMaster completes all 9 tasks. ChatGPT fails at quality control in most tasks (2/9). AutoBA completes basic clustering but lacks visualization and fails at advanced analyses (3/9).

Table S50: **ScRNA-seq clustering.** ChatGPT fails at quality control; AutoBA succeeds but lacks visualization; BioMaster completes full analysis.

**scRNA-seq clustering**	**Data Reading and Initialization**	**Quality Control (QC)**	**Normalization and Highly Variable Gene Selection**	**Dimensionality Reduction**	**Clustering Analysis**
Tools	scanpy	scanpy	scanpy	scanpy	scanpy
ChatGPT	✓	wrongly use scanpy to do quality control	×	×	×
AutoBA	✓	✓	✓	✓	✓, but no visualization output
BioMaster	✓	✓	✓	✓	✓

Table S51: **ScRNA-seq differential expression (Part 1).** ChatGPT fails at QC; AutoBA fails at gene selection; BioMaster succeeds.

scRNA-seq for differentially expressed genes identification	Data Reading and Initialization	Quality Control (QC)	Normalization and Highly Variable Gene Selection
Tools	scanpy	scanpy	scanpy
ChatGPT	✓	wrongly use scanpy to do quality control	×
AutoBA	✓	✓	failed to use scanpy to do highly variable gene selection
BioMaster	✓	✓	✓

Table S52: **ScRNA-seq differential expression (Part 2).** Only BioMaster completes dimensionality reduction, clustering, and differential analysis.

scRNA-seq for differentially expressed genes identification	Dimensionality Reduction	Clustering Analysis	Whole-Cell Differential Expression Analysis
Tools	scanpy	scanpy	scanpy, pandas
ChatGPT	×	×	×
AutoBA	×	×	×
BioMaster	✓	✓	✓

Table S53: **ScRNA-seq marker gene identification (Part 1).** ChatGPT fails at QC; AutoBA fails at gene selection; BioMaster succeeds.

**Single-cell data analysis Find marker genes based on count matrix**	**Data Reading and Initialization**	**Quality Control (QC)**	**Normalization and Highly Variable Gene Selection**	**Dimensionality Reduction**
Tools	scanpy	scanpy	scanpy	scanpy
ChatGPT	✓	wrongly use scanpy to do quality control	×	×
AutoBA	✓	✓	failed to use scanpy to do highly variable gene selection	×
BioMaster	✓	✓	✓	✓

Table S54: **ScRNA-seq marker gene identification (Part 2).** Only BioMaster successfully identifies and filters marker genes.

**Single-cell data analysis Find marker genes based on count matrix**	**Clustering Analysis**	**Whole-Cell Differential Expression Analysis**	**Filter marker genes**
Tools	scanpy	scanpy, pandas	pandas
ChatGPT	×	×	×
AutoBA	×	×	×
BioMaster	✓	✓	✓

Table S55: **ScRNA-seq top 5 marker genes (Part 1).** ChatGPT fails at QC; AutoBA fails at gene selection; BioMaster succeeds.

**Single-cell data analysis Identify top5 marker genes**	**Data Reading and Initialization**	**Quality Control (QC)**	**Normalization and Highly Variable Gene Selection**	**Dimensionality Reduction**
Tools	scanpy	scanpy	scanpy	scanpy
ChatGPT	✓	wrongly use scanpy to do quality control	×	×
AutoBA	✓	✓	failed to use scanpy to do highly variable gene selection	×
BioMaster	✓	✓	✓	✓

Table S56: **ScRNA-seq top 5 marker genes (Part 2).** Only BioMaster successfully identifies top 5 marker genes per cluster.

**Single-cell data analysis Identify top5 marker genes**	**Clustering Analysis**	**Whole-Cell Differential Expression Analysis**	**Find top 5 marker genes**
Tools	scanpy	scanpy, pandas	pandas
ChatGPT	×	×	×
AutoBA	×	×	×
BioMaster	✓	✓	✓

Table S57: **Complete scRNA-seq analysis (Part 1).** ChatGPT fails at QC; AutoBA and BioMaster complete basic workflow.

scRNA-seq analysis	Data Reading and Initialization	Quality Control (QC)	Normalization and Highly Variable Gene Selection	Dimensionality Reduction	Clustering Analysis
Tools	scanpy	scanpy	scanpy	scanpy	scanpy
ChatGPT	✓	wrongly use scanpy to do quality control	×	×	×
AutoBA	✓	✓	✓	✓	✓
BioMaster	✓	✓	✓	✓	✓

Table S58: **Complete scRNA-seq analysis (Part 2).** AutoBA fails at marker gene output; only BioMaster completes advanced analyses.

scRNA-seq analysis	Whole-Cell Differential Expression Analysis	Find top 20 marker genes	Cell Type Annotation	Pseudotime Trajectory Analysis
Tools	scanpy, pandas	pandas	scanpy, celltypist, pandas, matplotlib	scanpy, matplotlib
ChatGPT	✓	×	×	×
AutoBA	✓	fail in outputting marker genes file	×	×
BioMaster	✓	✓	✓	✓

Table S59: **Complete scRNA-seq analysis (Part 3).** Only BioMaster succeeds at purity, heterogeneity, and regulatory network analysis.

scRNA-seq analysis	Purity Analysis	Functional Heterogeneity Analysis	Regulatory Network Analysis
Tools	scanpy, pandas, numpy, scipy	scanpy, pandas	scanpy, decoupler, pandas
ChatGPT	×	×	×
AutoBA	×	×	×
BioMaster	✓	✓	✓

15 Comparative Analysis: Nanopore Sequencing Technologies

Overview

Tables S60–S67 evaluate Nanopore workflows including DNA methylation calling, event alignment, and comprehensive DNA sequencing (12 steps). BioMaster completes all tasks. AutoBA fails at dorado basecalling and minimap2 alignment. ChatGPT generates incorrect workflows using wrong tools (nanopolish, BWA-MEM, GATK).

Table S60: **Nanopore DNA methylation.** AutoBA fails due to incorrect dorado usage; BioMaster successfully identifies methylation.

**Nanopore Identifying DNA methylation**	**using dorado basecaller to perform modified basecalling from pod5 files and align to reference genome**	**Use dorado summary to generead-level sequencing information, including methylation details**
Tools	dorado	dorado
AutoBA	stuck due to wrongly used dorado tools	×
BioMaster	✓	✓

Table S61: **ChatGPT plan for Nanopore methylation.** ChatGPT generates incorrect workflow resulting in empty output.

**plan generated by ChatGPT**	**Basecall raw nanopore pod5 files to generate fastq reads**	**Align the basecalled reads to the reference genome**	**Convert SAM to a sorted, indexed BAM file**	**Call DNA methylation from the nanopore raw signal data and alignment information**
Tools	dorado	bwa	samtools	Megalodon
ChatGPT	empty output (due to wrong use of dorado)	×	×	×

Table S62: **Nanopore event alignment.** AutoBA incorrectly uses minimap2; BioMaster completes alignment successfully.

**Nanopore Event alignment**	**Map the nanopore fastq file to the reference genome**	**Convert SAM to a sorted, indexed BAM file**
Tools	minimap2	samtools
AutoBA	wrongly used minimap2 to read files	×
BioMaster	✓	✓

Table S63: **ChatGPT plan for Nanopore event alignment.** ChatGPT uses nanopolish incorrectly, generating wrong output.

**plan generated by ChatGPT**	**index the set of FAST5 files**	**extract and align the raw signal data**
Tools	nanopolish	nanopolish
ChatGPT	✓	wrong output due to wrong use of nanopolish eventalign

Table S64: **Nanopore DNA sequencing (Part 1).** AutoBA fails at basecalling; BioMaster completes QC and FASTQ conversion.

Nanopore DNA seq. analysis	Simplex basecalling	Read-level summary	BAM to FASTQ conversion	FASTQ QC
Tools	dorado	dorado	samtools	NanoPlot
AutoBA	Stuck due to wrong using of dorado tools	×	×	×
BioMaster	✓	✓	✓	✓

Table S65: **Nanopore DNA sequencing (Part 2).** Only BioMaster successfully filters reads and removes contamination.

Nanopore DNA seq. analysis	Low-quality read filtering	Host contamination removal	BAM to FASTQ conversion	QC of filtered reads
Tools	NanoFilter	minimap2	samtools	NanoPlot
AutoBA	×	×	×	×
BioMaster	✓	✓	✓	✓

Table S66: **Nanopore DNA sequencing (Part 3).** Only BioMaster completes reference mapping and generates statistics.

Nanopore DNA seq. analysis	Reference genome mapping	SAM sorting and indexing	Alignment statistics	Results consolidation
Tools	minimap2	samtools	samtools	bash
AutoBA	×	×	×	×
BioMaster	✓	✓	✓	✓

Table S67: **ChatGPT plan for Nanopore DNA sequencing.** ChatGPT plan fails at basecalling, preventing downstream analysis.

Plan generated by ChatGPT	POD5 to FASTQ conversion	Read alignment	SAM to BAM conversion	Variant calling	Cross-species contamination check
Tools	dorado	BWA-MEM	samtools	GATK	BWA-MEM
ChatGPT	Get empty output due to wrong use of dorado	×	×	×	×

16 Comparative Analysis: Comprehensive Whole Genome Sequencing

Overview

Tables S68–S71 evaluate comprehensive WGS pipelines including SNV, CNV, SV calling, annotation, and antibiotic resistance gene identification. BioMaster completes all 12 steps. AutoBA and ChatGPT fail at advanced variant calling and annotation. For SV analysis without matched normal samples, BioMaster completes all steps while AutoBA and ChatGPT achieve partial completion.

Table S68: **Comprehensive WGS analysis (Part 1).** All systems complete initial processing steps successfully.

WGS analysis	SRA to FASTQ conversion	Quality control	Read alignment	SAM to BAM conversion
Tools	fastq-dump	fastp	BWA	samtools
BioMaster	✓	✓	✓	✓
AutoBA	✓	✓	✓	✓
BioMaster-ChatGPT	✓	✓	✓	✓

Table S69: **Comprehensive WGS analysis (Part 2).** Only BioMaster completes CNV, SV detection, and SNV annotation.

WGS analysis	BAM processing	SNV calling	CNV & SV detection	SNV annotation
Tools	picard, samtools	bcftools	delly	annovar
BioMaster	✓	✓	✓	✓
AutoBA	×	×	×	×
BioMaster-ChatGPT	✓	×	×	×

Table S70: **Comprehensive WGS analysis (Part 3).** Only BioMaster completes SV annotation, VCF merging, and antibiotic resistance gene identification.

WGS analysis	CNV & SV annotation	VCF files merging	VCF to FASTA conversion	Antibiotic resistance gene identification
Tools	annovar	vcftools	bcftools, vcf2fasta	abricate
BioMaster	✓	✓	✓	✓
AutoBA	×	×	×	×
BioMaster-ChatGPT	×	×	×	×

Table S71: **WGS SV analysis without normal sample.** AutoBA and ChatGPT encounter errors; BioMaster successfully detects SVs.

**WGS SV analysis without normal sample**	**SRA to FASTQ conversion**	**Quality control**	**Alignment & SAM to BAM**	**BAM processing**	**SV detection**
Tools	fastq-dump	fastp	bwa, samtools	samtools	delly
BioMaster	✓	✓	✓	✓	✓
AutoBA	✓	×	✓	✓	×
BioMaster-ChatGPT	✓	×	✓	×	×

17 Comparative Analysis: Metagenomic Data Processing

Overview

Table S72 evaluates metagenomic analysis using R-based phyloseq workflows. AutoBA successfully completes phyloseq object creation, filtering, and visualization. ChatGPT and BioMaster encounter R script errors. This represents a domain where AutoBA demonstrates superior R package handling.

Table S72: **Metagenomic analysis.** ChatGPT and BioMaster have script errors; AutoBA successfully completes phyloseq analysis.

**Metagenomic analysis**	**Create a phyloseq-class object**	**Filter the created phyloseq-class object**	**Plot the composition figure**
**Tools**	create_phyloseq	BiocManager, devtools, phyloseq, microbiome, preprocessCore	microbiome, caret, file2meco
ChatGPT	error script	×	×
AutoBA	error script	×	×
BioMaster	✓	✓	✓

18 User Case

Setup and tasks. We recruited five novice users with no prior bioinformatics training (four undergraduates and one master's student). Each participant attempted two single-cell RNA-seq tasks: (1) clustering and (2) differential expression analysis (DEG). Participants were given only public README-level instructions and asked to iteratively refine their own prompts (e.g., "help me to do single-cell transcriptomics differential expression gene analysis by scanpy, and save the visualization results as images"; "please help me do clustering by scanpy, squidpy, and save the visualization results as images"). Unless otherwise noted, model settings followed the defaults used in the main text (e.g., main/tool model o1-2024-12-17, embedding text-embedding-ada-002).

Quantitative summary. Across participants, both tasks were completed successfully. Relative to expert references on the same datasets, clustering achieved a median concordance of ≈ 0.75 (e.g., ARI/NMI), while DEG exhibited a median top-k gene overlap of ≈ 0.85 (e.g., Jaccard). Divergences primarily reflected conservative/aggressive parameter choices (e.g., filtering thresholds, cluster resolution) rather than execution failures.

Representative prompts used by participants. help me to do single-cell transcriptomics differential expression gene analysis by scanpy, and save the visualization results as images; please help me do clustering by scanpy, squidpy, and save the visualization results as images.

Participant feedback

User A. Male, China, Sichuan Agricultural University, School of Information Engineering, Internet of Things, undergraduate.

> The user experience with this bioinformatics agent was excellent. The tool is simple to use, and the API configuration is remarkably straightforward, allowing users to get started quickly. The tool is well-designed for stability, featuring a built-in five-retry mechanism to ensure task completion. Furthermore, restarting after an interruption is very convenient and does not result in lost progress. Regarding its core functionality, the tool efficiently completed the single-cell transcriptomics differential expression analysis using Scanpy. The analysis results feature high-quality visualizations; key outputs such as the Leiden clustering plot and the volcano plot were generated clearly. The tool successfully outputted the marker genes along with their detailed metrics (e.g., logFC, p-values), providing a solid data foundation for subsequent research. A particularly commendable feature is the tool's ability to directly save visualization results as images on demand. This fully satisfies the user's need for a one-step process to acquire materials for reports. Furthermore, the selected configurations for the main model ("o1-2024-12-17") and the embedding model ("text-embedding-ada-002") were appropriate and operated smoothly. Overall, this agent tool is accurate, efficient, and easy to use, demonstrating high value in the bioinformatics analysis workflow.

User B. Male, China, Sichuan Agricultural University, School of Information Engineering, Internet of Things, undergraduate.

> Using BioMaster was extremely positive. As an undergraduate new to bioinformatics, I was surprised that I could run full single-cell workflows without understanding tool parameters, environment setup, or pipeline orchestration. I only described my goals in natural language, and BioMaster automatically planned, executed, debugged, and verified the entire workflow end-to-end with almost no intervention. All tasks (differential expression, clustering, spatial clustering, marker

identification) were executed using the following configurations: Main Model o1-2024-12-17, Tool Model o1-2024-12-17, and Embedding Model text-embedding-ada-002. The model configuration worked smoothly across the entire workflow, and the selection was flexible enough to meet performance requirements while still maintaining cost efficiency. Additionally, BioMaster supported both local deployment and API-based execution modes, enabling flexible usage depending on available resources and computational environments. During execution, all four prompts achieved a 100% completion rate. The outputs were stable, deterministic, and highly reproducible. Each task produced structured standardized artifacts, visualizations, and summary metrics that were directly suitable for downstream biological interpretation and scientific research usage. The Gradio UI significantly lowered the barrier to use — users only needed to provide Goal, Data List, Model selection, and execution mode, and the system handled everything else transparently. Runtime visualization of plans, step scripts, debug traces, and results further enhanced user trust and controllability. Compared to traditional prompt→error→debug→rerun loops, BioMaster completely removed the initial barrier that usually blocks beginners like me at the very first step. The system provided stable structured outputs, visualizations, and verified standardized files ready for interpretation. The live UI plan visualization + debug trace made me feel safe and in control, even though I do not yet fully understand every technical detail. BioMaster preserved full logs and fully reproducible shell scripts, allowing me to inspect exactly what was executed, and optionally re-run the generated .sh files manually for learning or debugging. For me, BioMaster transformed single-cell analysis from a multi-year skill barrier into something I can reliably execute today — even at the undergraduate beginner stage.

User C. Female, China, Sichuan Agricultural University, School of Information Engineering, Computer Science and Technology, undergraduate.

As an undergraduate majoring in computer science who has just started learning bioinformatics, I initially found it quite challenging to get started — there were so many tools, parameters, and workflows to understand, and even running a simple analysis often felt overwhelming. When I first tried to analyze single-cell data manually, I constantly ran into dependency errors and parameter issues. Using BioMaster completely changed that experience. My overall workflow became incredibly smooth, and I was finally able to focus on understanding the biology instead of struggling with technical setup. I was also pleasantly surprised by BioMaster's flexibility in model configuration and technical architecture. It supports multiple large language models such as OpenAI, DeepSeek, and Claude, and even allows running local Ollama models smoothly. Its dual RAG knowledge base design (PLAN RAG and EXECUTE RAG) is particularly clever — it keeps planning knowledge and execution knowledge separated, ensuring precise retrieval and avoiding confusion. On top of that, BioMaster supports over 30 common bioinformatics analysis types, including single-cell, RNA-seq, and WGS/WES, basically covering everything I might need as a beginner in the field. BioMaster also excels in visualization and reproducibility. It automatically generates key plots and result files during analysis and supports a checkpoint resume mechanism, so if something fails, it can continue from the last successful step instead of starting over — a huge time-saver. It also keeps detailed logs and shell scripts, letting me review exactly what was executed, which helps me both learn and troubleshoot. The Gradio GUI is especially friendly for newcomers, while the command-line mode is great for more advanced users. During my tests, I asked it to generate visualization outputs in PNG, JPG, and PDF formats — all worked perfectly. What really amazed me was when I requested an HTML report: it automatically created a complete research report that was detailed and well-organized, even covering aspects I hadn't considered. Overall, BioMaster delivers accurate, well-structured, and efficient outputs. The generated analysis reports and scripts are clean, parameterized properly, and can be directly used for publication or downstream analyses. The interface is intuitive and user-friendly, allowing me to complete tasks that used to take hours or days in a fraction of the time. For someone like me who's just stepping into bioinformatics, BioMaster is truly both smart and practical — it not only saves time but also helps me genuinely understand how a full analysis pipeline works.

User D. Male, China, Sichuan Agricultural University, School of Information Engineering, Computer Science and Technology, undergraduate.

As a beginner in bioinformatics, I have greatly benefited from BioMaster. Traditional analysis often fails due to errors in manually assembling command-line tools, making it inefficient. BioMaster transforms this complexity into a simple "task description," significantly lowering the learning threshold for those without deep specialized backgrounds. The clear configuration files and friendly Gradio UI make it immediately accessible, allowing me to focus more on interpreting biological data. Tasks that previously took days now complete in hours with excellent analysis quality. The system's reliability and efficiency comes from its superior engineering architecture, centered on the Multi-Agent Collaboration System. By deploying specialized Agents (PLAN, TASK, DEBUG, CHECK), BioMaster mitigates the common "error propagation problem" in long pipelines. The DEBUG Agent is particularly impressive—it intelligently analyzes error types and generates targeted solutions rather than simply retrying. Additionally, extensive model compatibility (OpenAI, Claude, local Ollama) provides crucial flexibility in balancing data privacy and computational budgets. Besides, BioMaster is extremely practical. It offers both fully automated and manual review modes—I can learn commands line by line in manual mode before switching to automation for efficiency. The detailed debug_output logs and preserved shell scripts make the entire process transparent, providing vital documentation for methodology sections and ensuring reproducibility. In summary, BioMaster is a useful tool that applies advanced agent technology to deliver a reliable, efficient, and user-friendly solution for bioinformatics.

User E. Female, China, University of Electronic Science and Technology of China, School of Information and Software Engineering, Software Engineering, Master's student.

The feedback for using the bioinformatics agent is highly positive. The tool effectively completed the task of single-cell transcriptomics differential expression gene analysis using Scanpy, as requested. The key visualizations, such as the Leiden clustering plot and the volcano plot, were generated without any issues. The tool also successfully outputted the marker genes, providing detailed information on each gene, including scores, logFC, p-values, and adjusted p-values. The model configurations, such as selecting the main model ("o1-2024-12-17") and embedding model ("text-embedding-ada-002"), were appropriately set and worked seamlessly throughout the analysis. The API configurations were also correctly implemented, ensuring smooth integration with the remote services for data retrieval and processing. The tool was able to process the provided single-cell data and generate the required visualizations efficiently. One of the standout features was the tool's ability to save the visualization results as images, as requested. This feature is particularly useful for users who need to present or further analyze the visual output. The user's goal of having the results saved directly from the tool without extra steps was met successfully, and the output images were stored in the expected formats, ready for immediate use. In terms of the tool's overall performance, the outputs were both accurate and timely, and the process was user-friendly. The clear and structured format of the results, with essential metrics such as logFC and p-values included for each gene, provides valuable insights for downstream analyses. The visualizations were not only informative but also aesthetically pleasing, enhancing the overall quality of the analysis.
